# Hierarchical Bayesian Integrated Modeling of Age- and Sex-Structured Wildlife Population Dynamics

**DOI:** 10.1007/s13253-024-00634-w

**Published:** 2024-07-02

**Authors:** Sabyasachi Mukhopadhyay, Hans-Peter Piepho, Sourabh Bhattacharya, Holly T. Dublin, Joseph O. Ogutu

**Affiliations:** 1 Biostatistics Unit (340C), Institute of Crop Science, University of Hohenheim, Fruwirthstrasse 23, 70599 Stuttgart, Germany; 2 Indian Institute of Management, Udaipur, India; 3 ISRU, Indian Statistical Institute, 203, B.T. Road, Kolkata, -108 India; 4 IUCN ESARO, Wasaa Conservation Centre, P.O. Box 68200, 00200 Nairobi, Kenya

**Keywords:** Animal population dynamics, Bayesian modeling, Birth and juvenile recruitment rates, Integrated state-space model, Markov chain Monte Carlo, Survival rates and sex ratio

## Abstract

Biodiversity of large wild mammals is declining at alarming rates worldwide. It is therefore imperative to develop effective population conservation and recovery strategies. Population dynamics models can provide insights into processes driving declines of particular populations of a species and their relative importance. But there are insufficient tools, namely population dynamics models for wild herbivores, for characterizing their decline and for guiding conservation and management actions. Therefore, we have developed a model which can serve as a tool to fill that void. Specifically, we develop an integrated Bayesian state-space population dynamics model for wildlife populations and illustrate it using a topi population inhabiting the Greater Mara-Serengeti Ecosystem in Kenya and Tanzania. The model integrates ground demographic survey with aerial survey monitoring data. It incorporates population age and sex structure and life history traits and strategies and relates birth rates, age-specific survival rates and sex ratios with meteorological covariates, prior population density, environmental seasonality and predation risk. It runs on a monthly time step, enabling accurate characterization of reproductive seasonality, phenology, synchrony and prolificacy of births, juvenile and adult recruitments. Model performance is evaluated using balanced bootstrap sampling and by comparing model predictions with empirical aerial population size estimates. The hierarchical Bayesian model is implemented using MCMC methods for parameter estimation, prediction and inference and reproduces several well-known features of the Mara topi population, including striking and persistent population decline, seasonality of births, juvenile and adult recruitments. It is general and can be readily adapted for other wildlife species and extended to incorporate several additional useful features. Supplementary materials accompanying this paper appear on-line.

**Supplementary Information:**

The online version contains supplementary material available at 10.1007/s13253-024-00634-w.

## Introduction

Biodiversity is declining worldwide at such an alarming rate that biologists have christened the contemporary biodiversity loss as the sixth mass extinction (McCallum 2015; Ceballos et al. 2017). Large mammal populations are particularly at risk in many ecosystems. Across continental Africa, many populations of large mammal species are undergoing disturbing declines (Craigie et al. 2010; Chase et al. 2016). In Kenya, for example, large herbivore populations declined by 70% on average between 1977 and 2013 (Ogutu et al. 2011, 2016). But, thus far, integrated state-space population dynamics models for wildlife populations that incorporate realistic life history traits and strategies and fine temporal frames, such as monthly temporal resolution, have not been developed (but see e.g., Newman et al. 2006; Iijima et al. 2013; Nadeem et al. 2016; Polansky et al. 2021 for models using annual time frames). Such models are crucial for understanding and quantifying how life history traits and strategies shape population responses to trophic interactions, natural environmental and anthropogenic stressors.

Therefore, it is imperative to advance our understanding of large herbivore population dynamics as a basis for developing species conservation and management and population recovery strategies. We can do this by building reliable models of large herbivore population dynamics that incorporate realistic life history traits and strategies and fine temporal frames. Reliable population dynamics models can help quantify and evaluate the relative importance of multiple processes driving declines of particular populations of a species.

For populations inhabiting seasonally variable environments and reproducing seasonally, such models can help quantify shifts in seasonality, phenology, synchrony and prolificity (proportion of breeding females that actually give birth per month) of births, juvenile recruitment and sex ratio in response to climate and other changes. The models can also be used to estimate population trajectories and assess likely population responses to conservation and management interventions, projected future scenarios of climate change, human population growth, socio-economic development, land use and other changes (Zhao et al. 2019a, b).

Animal population dynamics models often use independent data collected using various methods, such as ground demographic surveys and aerial surveys. Population dynamics models are also increasingly using information from multiple sources to make inferences on various features of populations. Notably, integrated population models are becoming widely used to combine different types of data from disparate sources to make joint inferences on animal population dynamics (Trenkel et al. 2000; Besbeas et al. 2005; Rhodes et al. 2011; Maunder et al. 2015; Mosnier et al. 2015; Schaub and Kéry 2022).

Here, we develop an integrated population dynamics model for large wild herbivores that integrates aerial survey data with fine-resolution ground demographic survey data. The model can be used to predict large herbivore population dynamics and evaluate the relative importance of various factors driving population dynamics. It accounts for influences on large herbivore population dynamics of variation in climatic components, notably rainfall, temperature and their interactions; predation, density dependence, population age and sex (adult sex ratio) structure, gestation length, weaning period, adult female pregnancy status, adult females available to conceive, females reaching the age of first-time conception, birth, juvenile and adult recruitment, age- and sex-specific survival rates and environmental seasonality. The model runs on a discrete monthly time step to reliably track temporal variation in female pregnancy status, birth, juvenile and adult recruitment, age- and sex-specific survival rates, adult sex ratio, population size and inter-annual variation in reproductive seasonality, phenology, synchrony and prolificity of births. The model is illustrated using the topi (*Damaliscus lunatus jimela*) population inhabiting the Masai Mara Ecosystem of south-western Kenya but is general and applicable to populations of other large herbivore species.

Our integrated state-space modeling approach has several attractive and desirable properties. (1) It integrates various data types from multiple sources, notably aerial surveys and demographic ground counts. (2) It incorporates realistic population life history traits and strategies, survival rates, sex ratios and trophic interactions, namely predation and competition. (3) It accommodates and permits straightforward representation of possibly complex nonlinear relationships between birth and adult recruitments, age-specific survival rates, adult sex ratio and multiple covariates. (4) It predicts the ecosystem-wide population size and distributes this among the ecosystem’s subregions, including those covered by the aerial surveys only. (5) The model uses a monthly time step, allowing accurate characterization of reproductive seasonality, phenology, synchrony and prolificity of birth and juvenile recruitment. (6) The model allows efficient computation of posterior distributions of many parameters and uses a flexible Transformation MCMC (TMCMC) technique to enhance computational efficiency and accelerate convergence of iterations. (7) The model is validated using balanced bootstrap sampling to generate multiple population trajectories and assess robustness. (8) It uses the Importance Resampling MCMC (IRMCMC) technique for the first time to accelerate MCMC iterations for multiple data sets each of which involves high computational costs. (9) Lastly, the model is robust and reliably reproduces well-known features of animal population dynamics and can be easily adapted for other species.

The rest of the paper is organized as follows. We describe the data in Sect. 2. The Bayesian state-space model is described in Sect. 3 along with an evaluation of its performance using balanced bootstrap samples. In Sect. 3.5, we formulate the birth and adult recruitment, age-specific survival and adult sex ratio models and other model components. In Sect. 4.1, prior distributions of the model are described. In Sect. 4.2, we discuss the full conditional distributions, full likelihood, convergence of the MCMC chain and model validation. In Sect. 5, we present results of applying the state-space model to the Mara-Serengeti topi population. Finally, in Sect. 6 we discuss the results and extensions of the model.

## The Data

We provide brief overviews of the data and the study population in Sects. 2.1 to 2.4 and supply additional details on the ground vehicle sample counts, aerial sample surveys, the Mara-Serengeti topi population, rainfall and temperature in Supplementary Sect. S.1. The full data dictionary providing definitions of all the variables and their transformations is also provided in Supplementary Sect. S.1.

### Ground Vehicle Age and Sex Composition Sample Surveys

Ground vehicle age and sex composition sample surveys of seven ungulate species, including topi (Fig. S2) were carried out monthly in the Maasai Mara National Reserve (Fig. S1b) and its adjoining pastoral lands for 174 months from July 1989 to December 2003. Approximate ages of topi in each size class are newborn (Age < 1 month), quarter size class (1 month $\le$ Age < 6 months), half-yearling class (6 months $\le$ Age < 19 months) and adults (19 months $\le$ Age). Adult topi were not aged but were sexed using the presence, size and shape of horns, dimorphic morphology of the external genitalia and other secondary sexual characters (Fig. S3). During the entire monitoring period, 91,582 topi were aged and 78,738 were sexed (Ogutu et al. 2008a, b). The age- and sex-structured topi ground counts are provided in Supplementary Sect. S.1 and described in Table S1.

### Aerial Sample Surveys

These data are independent of the ground vehicle sample age- and sex-structured counts. The Directorate of Resource Surveys and Remote Sensing of Kenya (DRSRS) monitored wildlife population size and distribution in the Maasai Mara Ecosystem (6665.6, Fig. S1b) using systematic reconnaissance flights from 1977 (Fig. S4). A total of 75 surveys were carried out in the ecosystem from 1977 to 2018 using 662 flights. But only the counts for July 1989 to December 2003 that overlapped the ground vehicle counts were used in the model. The total topi population size estimated from the aerial survey data is provided in Supplementary Sect. S.1 and described in Table S2.

### The Mara–Serengeti Topi Population

Topi is a resident grazer in the Mara–Serengeti ecosystem. There, births are seasonal, start in July and peak at the onset of the early rains in October–November, whereas conceptions peak at the start of the long rains in February–March. Births occur in all months but are rare from January to July (Ogutu et al. 2010, 2014a; Sinclair et al. 2000). The gestation period of 8 months is followed by a lactation period of 3 months. Consequently, topi young are weaned after 3–4 months and nursing ceases before conceptions. Topi thus take about 11 months from one conception cycle to the next and give birth to one young per year. The young go through a hiding stage before following their mothers (Estes 1991; Skinner and Chimimba 2005). The young, although initially unsteady on their legs for a few hours after birth, are able to keep up remarkably well with the swift pace of their parents within a day or two (Vesey-Fitz-Gerald 1955). Females attain sexual maturity after about 18 months. Topi pregnancy rate in Mara-Serengeti is 100% for adult females breeding for the second time or more but 86% for 2-year-old females breeding for the first time (Duncan 1975).

### Rainfall and Temperature

In African savannas, vegetation production, quantity and quality are controlled by rainfall (Boutton et al. 1988a; Deshmukh 1984). Rainfall seasonality generates and sustains seasonality in food availability and quality for large herbivores (Boutton et al. 1988b). Accordingly, rainfall indexes food availability and quality for savanna herbivores. Seasonal temperature fluctuations additionally affect food quality for herbivores by governing the retention period of green plant leaf through the dry season. Monthly rainfall was averaged over a network of 15 monitoring gauges spread over the Mara to account for spatial variation (Bartzke et al. 2018; Mukhopadhyay et al. 2019). The monthly averages of blended satellite station maximum and minimum temperatures data for each 5 km $\times$ 5 km grid cell in the Mara Ecosystem were also extracted from the Chirps data (Funk et al. 2015) and used as covariates. The rainfall and temperature components, the months covered by each component, moving averages, lags and lagged moving averages computed for each component and used as covariates of birth and recruitments, survival probabilities and adult sex ratio are described in Table 1.

**Table 1:** Rainfall and temperature components and the total topi population size, the months covered by each component, moving averages, lags and lagged moving averages computed for each component and used as covariates of recruitment rates, survival probabilities and adult sex ratio

Rainfall components				
Covariate	Months covered	Moving averages	Lags	Lagged moving averages
Monthly rainfall	Each month	Mavrain1–Mavrain5	Rain1–Rain5	Mavrain6-9, Mavrain6-10, Mavrain7-10, Mavrain7-11
Early wet season	Nov–Feb	Mavearlywet1–Mavearlywet3	Earlywet1	
Late wet season	Mar–Jun	Mavlatewet1–Mavlatewet3	Latewet1	
Early dry season	Jul–Aug	Mavearlydry1–Mavearlydry3	Earlydry1	
Late dry season	Sep–Oct	Mavlatedry1–Mavlatedry3	Latedry1	
Wet season	Nov–Jun	Mavdry1–Mavdry3	Wet1	
Dry season	Jul–Oct	Mavlatewet1–Mavlatewet3	Dry1	
Annual	Nov–Oct	Mavannual1–Mavannual3	Annual1	
Temperature Components				
Covariate	Months covered	Moving averages	Lags	
Min	Nov–Oct	Mavmin1–Mavmin5	Min1–Min5	
Max	Nov–Oct	Mavmax1–Mavmax5	Max1-Max5	
Population component (derived from the aerial survey data)				
Covariate	Months covered	Moving averages	Lags	
Population size	Each month		Pop1-Pop8	

## The Integrated Population Dynamics State-Space Model

We construct a general age- and sex-structured population dynamics model for large wild herbivores. The model uses the number of animals observed in the ground and aerial surveys (Sects. 2.1 and 2.2) that are only samples from the unobserved (latent or hidden) true population about which we wish to make inferences. Thus, the population dynamics model entails two parallel but connected processes. The first is the unobserved true population that evolves over time (called state process), and the second is the variation in the observed counts over time (called observation process). A common approach to modeling both processes simultaneously is to use state-space models (Buckland et al. 2007; Maunder et al. 2015; Newman et al. 2009; Thomas et al. 2005).

### Methods

We develop an integrated population dynamics state-space model which couples a hypothetical (or latent) mechanistic model of large herbivore population dynamics (state process model), with a statistical observation model of aerial survey and ground demographic data (observation process model). The approach of integrating two time series is similar to those reviewed in Auger-Méthé et al. (2021). In the state-space model, the state process model predicts the true but unknown future state of the large herbivore population given its current state. The observation model weights the predictions by the likelihood of the data and thus links the process model to the observations. Consequently, the model integrates the aerial survey monitoring data with the contemporaneous but independent ground demographic survey data.

The state-space model involves quantifying birth recruitment and survival rates of various age and sex classes of the population and sex ratio as functions of climatic factors (e.g., rainfall and temperature), intraspecific competition, population density, predation and seasonality. Our model shares similarities with the general approaches proposed by Buckland et al. (2004) and Newman et al. (2006, 2014) and uses multiple data sets similar to Newman and Lindley (2006) but also has some notable differences. In particular, we make different distributional assumptions for the initial states and the other components of the state and observation processes. Most crucially, our approach differs from theirs with respect to several structural assumptions and our proposal to model transition probabilities of the state process using log-linear models in which covariates such as rainfall, temperature and population density are used as covariates of birth recruitment and survival probabilities and adult sex ratio, similar to the Bayesian approach of Brooks et al. (2004) and Polansky et al. (2021). Also, unlike Newman et al. (2006), we illustrate our model using a non-migratory species, but the model can be easily adapted for migratory species. Lastly, our model incorporates several key life history traits and strategies crucial to understanding large herbivore population biology and dynamics and uses rare long-term, fine-resolution ground demographic and aerial survey monitoring data.

In Sects. 3.2 to 3.6, we describe the state process and observation models and the associated notations. In particular, we describe parameters of the state process model and how they link birth and adult recruitments and age- and sex-specific survival probabilities and adult sex ratio with covariates in Sects. 3.5 to 3.6. Accurate estimation of birth and adult recruitments and age- and sex-specific survival probabilities and sex ratio is therefore a crucial step in developing the state-space model. As a result, the model explicitly allows for the dependence of birth and adult recruitments and age- and sex-specific survival probabilities and sex ratio on food availability and quality, density-dependent intraspecific competition for food and large carnivore predation. Influences of these factors are indexed by past rainfall, minimum and maximum temperatures and their interactions, prior total population size, indexing density dependence, and environmental seasonality.

The relatively large number of states and parameters considerably complicates their estimation using classical techniques. We overcome this difficulty using a flexible Bayesian state-space model and present forms of the prior, the full likelihood and full conditional distributions in Sects. 4.1 and 4.2 and in Supplementary Sect. S.2.

### State and Observation Vector Components or Structure

In this section, we define the state and observation vector components. To construct the age- and sex-structured state-space model, we first introduce notations for different topi age and sex classes at time *t*, expressed in units of months, and for the observation process as follows. We assume throughout that all births or recruitments occur at the end of each month. The components of the vectors of observations, $z_{t}$, are then defined as follows. *n*(*t*) = observed number of newborns, *q*(*t*) = observed number of quarter-sized animals, *h*(*t*) = observed number of half-yearlings, *f*(*t*) = observed number of adult females, *m*(*t*) = observed number of adult males. The state process involves the same age and sex classes. The true but unknown numbers of animals in each age and sex class constitute the components of vectors for states, $Y_{t}$, and are denoted by *N*(*t*) = actual number of newborns, *Q*(*t*) = actual number of quarters, *H*(*t*) = actual number of half-yearlings, *F*(*t*) = actual number of adult females, comprising surviving adult females and new recruits that join the adult female class in month *t*, $F_a(t)$; *M*(*t*) = actual number of adult males, consisting of new recruits that join the adult male class in month *t*, $M_a(t)$, and surviving adult males, $M_b(t)$. State-space models for the state and observation vectors are detailed in Table 2.

### Filling Components of the State Vector, $Y_{t}$

In this section, we discuss how the components of the state vectors introduced in Sect. 3.2 are defined and calculated. Since the exact age of topi in months is hard to determine through visual field observation, animals were only assigned to age and sex classes. However, the probability of survival likely varies with age and other temporally varying covariates, such as rainfall that governs food availability and quality in African savannas. For example, a newborn topi must survive the first month of its life to join the quarter size class. Likewise, a quarter size topi must survive through five consecutive months before graduating to the half-yearling class. So, we assign topi in the quarter and half-yearling age classes to actual ages in months as follows. *G*(*t*, *k*) = Number of age *k* individuals in month *t* in the population with actual ages lying between $k-1$ and *k* ($k-1$-th month included but *k*-th month excluded, i.e., in the half-open interval [*k*-1, *k*)), $k = 2, \ldots , 19$ months. Note, for example, therefore that $Q(t) = \sum _{k=2}^{6} G(t, k)$, where *k*=2 denotes the half-open interval [1,2), and that $H(t) = \sum _{k=7}^{19}G(t, k)$. Also, *G*(*t*, 1) and *N*(*t*) are actually equivalent and indicate the newborn at time *t*.

For adult females, tracking the reproductive cycle is essential for understanding population dynamics. Young topi start reproducing at about 19 months of age. The topi reproductive cycle spans 11 months, including 8 months for gestation and 3 months for lactation. We assume that a female cannot conceive during this 11 month period. If pregnancy is prematurely terminated, however, then a fresh conception may occur within the 11 month period. But we do not have data to estimate the probability that a pregnant female topi fails to carry pregnancy to term and so do not consider it in the model. We track the pregnancy status of adult females in each of the 11 months spanned by the reproductive cycle as follows: $P(t, \ell )$ = Number of adult females at time *t* that gave birth exactly $\ell$ months ago, $\ell = 1, \ldots , 11$; *P*(*t*, 12) = Number of adult females at time *t* that gave birth at least 12 months ago.

**Fig. 1:**
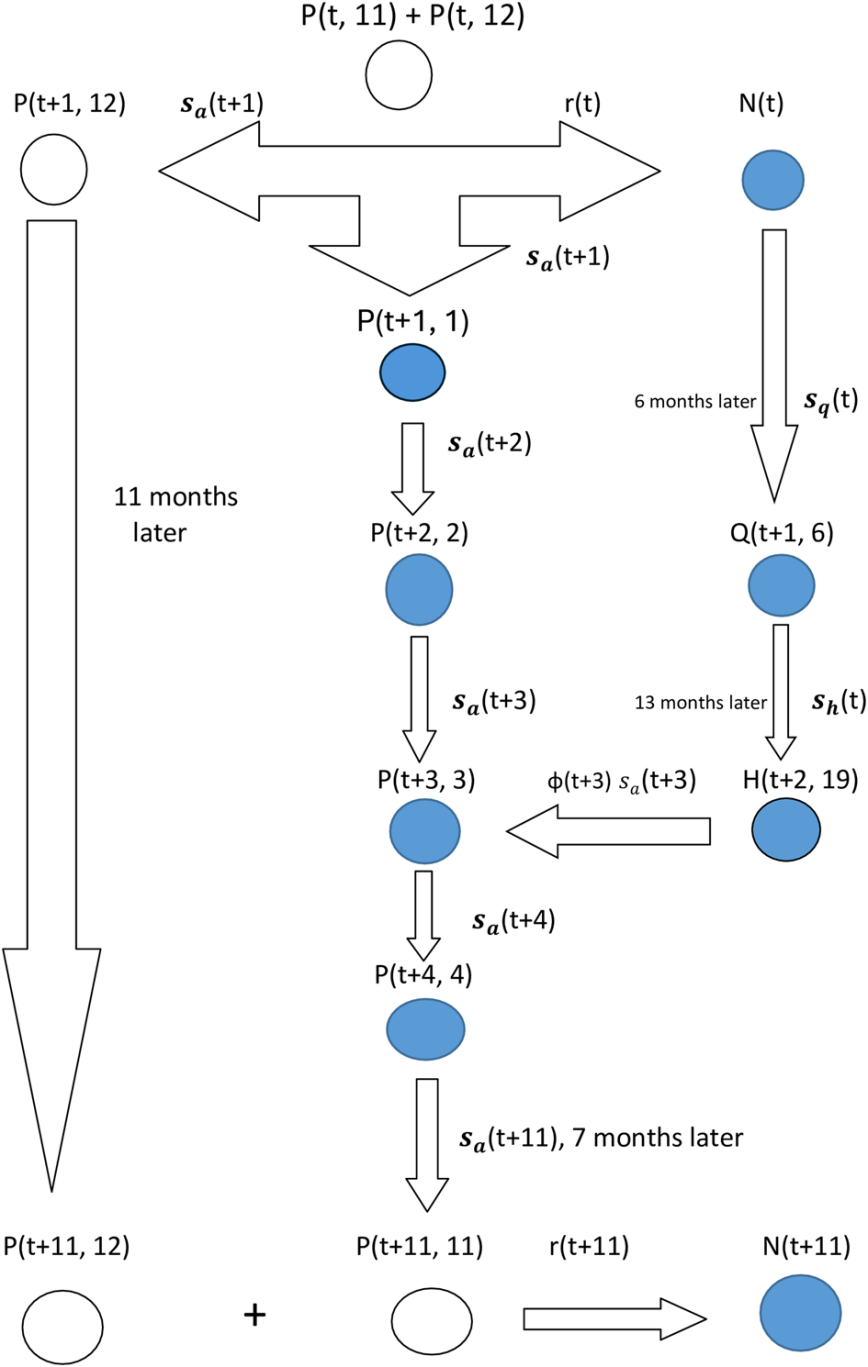
Schematic flow chart showing the reproduction and recruitment processes in topi. The notations are defined in the text. This schematic is complemented by the Leslie matrix formulation of the topi population dynamics model in Sect. S.5

The recruitment of half-yearlings into the adult class and the transition of adults from time *t*-1 to *t* are represented as follows. Male and female half-yearlings at time $t-1$, denoted by $G(t-1, 19)$, that join the adult class at time *t* are denoted by $M_{a}(t)$ and $F_{a}(t)$, respectively. Similarly, adult males at time $t-1$, denoted by $M(t-1)$, that remain in the adult male class at time *t*, are denoted by $M_{b}(t)$. Mathematically, we can write $M(t) = M_{a}(t) + M_{b}(t)$. We denote by $\phi (t)$ the probability that an individual graduating from the half-yearling class to the adult class is a female at time *t* or the probability of being female (henceforward referred to as sex ratio). $F_{a}(t)$ is therefore the number of new adult female recruits that can conceive at time *t* and give birth 8 months later. So, we add the new adult female recruits to *P*(*t*, 3), the number of adult females that gave birth exactly 3 months ago and are alive at time *t*, and track future changes in the resulting total number. Note that $F(t) = \sum _{\ell =1}^{12}P(t, \ell )$. From the preceding definitions, it follows that a total of only $P(t, 11)+P(t, 12)$ females can conceive at time *t*. All these processes are illustrated diagrammatically in Fig. 1.

The time *t* = 0 for our data corresponds to June 1989, one month before the start of the ground sample surveys in July 1989. To model population size at time $t=1$, we need to know the initial population size at time *t* = 0. We denote the initial population distribution by *N*(0) = Number of newborns at time *t* = 0; *G*(0, *k*) = Number of animals with actual ages lying between $k-1$ and *k* ($k-1$ included but *k* excluded) months at time *t* = 0, $2\le k \le$19; $P(0, \ell )$ = Number of adult females that gave birth $\ell$ months before ($1\le \ell \le 12$) time *t* = 0; *M*(0) = Number of adult males at time *t* = 0. Note that for $2\le k \le 6$, *G*(0, *k*) denotes the number of quarters with ages ranging between $k-1$ and *k* months and for $7\le k \le 19$, *G*(0, *k*) represents the number of half-yearlings with actual ages lying between $k-1$ and *k*.

### The State Process Transitions

This section describes how the state process or the state vector ($Y_{t}$) defined in Sects. 3.2 and 3.3 transitions or changes to $Y_{t+1}$. The stochastic topi population dynamics model for time $t = 1, \ldots , T$ can then be cast as in Table 2. The birth recruitment and the survival rates of different age and sex classes in Table 2 are defined as follows.

*r*(*t*) is the birth recruitment rate for breeding females at time *t* = $0, 1, \ldots , T$; $s_q(t)$ is the survival rate for quarter age groups at time *t* = $0, 1, \ldots , T$; $s_h(t)$ is the survival rate for half-yearling age groups at time *t* = $0, 1, \ldots , T$; $s_a(t)$ is the survival rate for adult age groups at time *t* = $0, 1, \ldots , T$ and $\phi (t)$ is the ratio of females that achieved adulthood from half-yearling age group at time *t* = $0, 1, \ldots , T$. The parameters *r*(*t*), $s_q(t)$, $s_h(t)$, $s_a(t)$ and $\phi (t)$ are all modeled as functions of covariates, with details provided later in Sect. 3.7.

The causes of large herbivore mortality can be classified into two broad categories—a) natural causes such as senescence (aging), starvation due to drought, competition for limiting food or disease and b) predation. Mortality due to such natural causes as starvation and competition can be explained by meteorological covariates such as rainfall and temperature, indexing food availability and quality, and density dependence, a proxy for competition. However, predation risk can be better explained by other factors, such as the density of large carnivores. So, for modeling the influences of these mortality sources we split the mortality probability into - P(mortality) = P(mortality due to natural causes)$\times$ P(mortality due to predation$\mid$ Survived mortality due to natural causes).

Let ${\tilde{r}}(t)$, ${\tilde{S}}_q(t)$, ${\tilde{S}}_h(t)$ and ${\tilde{S}}_a(t)$ denote the birth recruitment rate for newborns and survival rate of the other age classes in the absence of predation. We denote the predation risks for various age classes by ${\tilde{P}}_{r}(t)$ for newborns, ${\tilde{P}}_{q}(t)$ for quarter size class, ${\tilde{P}}_{h}(t)$ for half-yearling size class and ${\tilde{P}}_a(t)$ for adults.

**Table 2:** State-space and observation process model formulations

Model	Distributions
State process for	$N(t) \sim Binomial(P(t, 11)+P(t, 12), r(t))$
newborns (*N*(*t*)),	$G(t, 1) \sim Binomial(N(t-1), s_q(t))$
quarter size	$G(t, k) \sim Binomial(G(t-1, k-1), s_q(t)), k = 2, \ldots , 6$
(*G*(*t*, *k*), $1\le k \le 6$),	$G(t, k) \sim Binomial(G(t-1, k-1), s_h(t)), k = 7, \ldots , 19$
half-yearlings	$(F_{a}(t), M_{a}(t)) \sim Multinomial(G(t-1, 19), \phi (t)\times s_a(t), (1-\phi (t))\times s_a(t))$
(*G*(*t*, *k*), $7 \le k \le 19$),	$M(t) \sim Binomial(M_{a}(t-1)+M_{b}(t-1), s_a(t))$
adult females ($F_{a}(t)$) and	
adult males (*M*(*t*)), $1\le t\le T$	
State process	$P(t, 1) \sim Binomial(N(t-1), s_a(t))$
for breeding adult females	$P(t, k) \sim Binomial(P(t-1, k-1), s_a(t)), k = 2, 3$
for time $1\le t\le T$	$P(t, 4) \sim Binomial(P(t-1, 3)+F_{a}(t-1), s_a(t))$
	$$P(t, k) \sim Binomial(P(t-1, k-1), s_a(t)), k = 5, \ldots , 11$$
	$$P(t, 12) \sim Binomial(P(t-1, 11)+P(t-1, 12)-N(t-1), s_a(t))$$
Initial state process	$N(0) \sim Binomial(P(0, 11)+P(0, 12), r(0))$
for newborns at time *t* = 0	
Observation process	$n(t)\mid N(t) \sim Poisson(\lambda _{1}(t))$, $q(t)\mid Q(t) \sim Poisson(\lambda _{2}(t))$,
	$h(t)\mid H(t) \sim Poisson(\lambda _{3}(t))$, $f(t)\mid F(t) \sim Poisson(\lambda _{4}(t))$, $m(t)\mid M(t) \sim Poisson(\lambda _{5}(t))$,
	with parameters $\lambda _{i}$, $i = 1, \ldots , 5$, each assumed to follow a gamma distribution as $Gamma(\alpha _{i}(t), \beta _{i}(t))$, $i = 1, \ldots , 5$, where $\alpha _{1}(t) = \frac{N^2(t)}{\sigma _{1}^2}$ and $\beta _{1}(t) = \frac{N(t)}{\sigma _{1}^2}$. Similarly, we define $\alpha _{2}(t) = \frac{Q^2(t)}{\sigma _{2}^2}$, $\beta _{2}(t) = \frac{Q(t)}{\sigma _{2}^2}$, $\alpha _{3}(t) = \frac{H^2(t)}{\sigma _{3}^2}$, $\beta _{3}(t) = \frac{H(t)}{\sigma _{3}^2}$, $\alpha _{4}(t) = \frac{F^2(t)}{\sigma _{4}^2}$, $\beta _{4}(t) = \frac{F(t)}{\sigma _{4}^2}$, $\alpha _{5}(t) = \frac{M^2(t)}{\sigma _{5}^2}$ and $\beta _{5}(t) = \frac{M(t)}{\sigma _{5}^2}$.
Integration with aerial survey	$\lambda _t^{T} \sim Gamma(\frac{(K_t B_t)^2}{\sigma _T^2}, \frac{K_t B_t}{\sigma _T^2})$, $\mho _{t} \sim Poisson(\lambda _t^{T})$, $\Psi _{t} \sim N(\mho _{t}, \sigma _{\Psi }^2)$

All the notations are explained in Sects. 3.2, 3.3, 3.4, 3.5, 3.6 and 3.7 in the text

As defined in Sect. 3.3, *P*(*t*, 1) is the number of females that gave birth exactly one month ago. We assume that the number of females that gave birth at time $t-1$ is the same as the number of newborns recorded at time $t-1$. This is a slight underestimate because of unobservable calf mortalities. We expect some calf mortality to occur between consecutive sampling periods (1 month) due to predation and other processes but have no calf mortality data to model such calf losses. However, such calf losses should be minimal because of the short interval between consecutive sampling periods ($\le$ 4 weeks). The population dynamics of adult females is thus modeled as in Table 2.

Note that the newborns at time *t* = 0, *N*(0), have a birth recruitment rate *r*(0), incorporating birth rate, survival rate and the proportion of hidden and other missed newborns in the first month of life, and the corresponding stochastic equation is given in Table 2.

We provide expressions for the topi population dynamics model as a Linear Gaussian State-Space Model (SSM) as an approximation in Sect. S.5. The Leslie matrix formulation (Caswell 2000) clarifies the SSM’s process structure and, combined with the use of a normal approximation to the SSM, permits the use of the Kalman filter to calculate the likelihood and make either frequentist or Bayesian inference.

### Modeling of the State Processes

Here, we first introduce notations for covariates of birth recruitment and survival rates and then explain how we determine the birth recruitment, sex ratio and age-specific survival functions as well as predation risk. Denoting time by *t* and the 174 months covered by the ground vehicle counts by $t = 1, \ldots , 174$, we introduce a set of notations for the covariates used to predict topi birth and adult recruitments, age-specific survival rates and adult sex ratio. The notations and their descriptions are summarized in Table 3.

**Table 3:** Notations and definitions for the covariates used for building the logit models as explained in Sects. 3.7

Notation for covariate	Description of notation for covariate
*month*(*t*) =	Calender month corresponding to January-December and numbered as 1, 2, $\ldots$, 12
$rain(7-11, t)$ =	Average total monthly rainfall including lags 6 to 10 at time *t* (i.e., 7 to 11 months before the birth month)
*Npop*(*t*) =	Total topi population size at lag 7 at time *t* (i.e., around conception time 8 months ago)
*mintemp*(*t*) =	Minimum temperature at time *t*
*maxtemp*(*t*) =	Maximum temperature at time *t*
$lagmin(\ell , t)$ =	Minimum temperature at lag $\ell$ at time *t*, $\ell = 1, 2, \ldots$, 11 (i.e., up to 4 months pre-conception)
$lagmax(\ell , t)$ =	Maximum temperature at lag $\ell$ at time *t*, $\ell = 1, 2, \ldots$, 11
*Apop*(*t*) =	Total population size at lag 1 at time *t*
$lagrain(\ell , t)$ =	Total monthly rainfall at lag $\ell$ at time *t*, $\ell = 0, 1, 2, \ldots , 11$. Note that *lagrain*(0, *t*) stands for total monthly rainfall at time *t*
*wet*1(*t*) =	Total wet season rainfall at lag 1 (i.e., in the immediately preceding year) at time *t*
*earlywet*1(*t*) =	Total early wet season (November–February) rainfall at lag 1 (i.e., in the immediately preceding year) at time *t*
*dry*1(*t*) =	Total dry season rainfall at lag 1 (i.e., in the immediately preceding year) at time *t*
$mavrain(\ell -p, t)$ =	Moving average of rainfall between lags $\ell$ and *p* ($\ell < p$) at time *t*

All the process rates considered in this section are modeled as functions of covariates and estimated by the state-space model. The method used to determine the functional forms of the relationships between the birth and adult recruitments, sex ratio and age-specific survival rates (${\tilde{r}}(t)$, ${\tilde{S}}_q(t)$, ${\tilde{S}}_h(t)$ and ${\tilde{S}}_a(t)$) and various covariates is described more fully in Supplementary Sect. S.4.4. The functional forms of the logit regression models relating birth and adult recruitments and age-specific survival rates and adult sex ratio to covariates are given by the equations in Table 4. The estimation of the initial values of the parameters of the birth recruitment, sex ratio and age-specific survival functions (Table 4) is done outside of, but the final parameter estimates are obtained by, the state-space model fitting.

**Table 4:** Functional relationships between the logits ($logit(p) = \log (\frac{p}{1-p})$) of recruitment and survival rates (*p*) and covariates

Rate	Equation
Birth recruitment	$logit\left( {\tilde{r}}(t)\right) = \gamma ^{R}_1+\gamma ^{R}_2 month(t)+\gamma ^{R}_3 month^2(t)+ \gamma ^{R}_4 month^3(t)+ \gamma ^{R}_5 rain(7-11, t)+\gamma ^{R}_6 rain^2(7-11, t)+$ $\gamma ^{R}_7 Npop(t)+\gamma ^{R}_8 mintemp(t)+ \gamma ^{R}_9 maxtemp(t)$
Quarter size survival	$logit\left( {\tilde{S}}_q(t)\right) = \sum _{k=1}^{12}\gamma ^{Q}_k \delta _{k}(t)+ \gamma ^{Q}_{13} dry1(t)+\gamma ^{Q}_{14} mavrain(3-4, t)$
Half-yearling survival	$logit\left( {\tilde{S}}_h(t)\right) = \sum _{k=1}^{12}\gamma ^{H}_k \delta _{k}(t)+ \gamma ^{H}_{13} earlywet1(t)$
Adult survival	$logit\left( {\tilde{S}}_a(t)\right) = \sum _{k=1}^{2}\gamma ^{A}_k \delta _{k}(t)+\gamma ^{A}_3 Apop(t)+ \gamma ^{A}_4 lagrain(4, t)+ \gamma ^{A}_5 lagrain(5, t)+\gamma ^{A}_6 lagrain(6, t)+\gamma ^{A}_7 lagrain(7, t)+ \gamma ^{A}_8 wet1(t)$
Adult recruitment	$logit\left( \phi (t)\right) = \sum _{k=1}^{2}\gamma ^{S}_k \delta _{k}(t)+\gamma ^{S}_3 wet1(t)+ \gamma ^{S}_4 dry1(t)+ \gamma ^{S}_5 lagrain(0, t)+\gamma ^{S}_6 rain(7-11, t)+\gamma ^{S}_7 mintemp(t)+ \gamma ^{S}_8 lagmin(2, t)+\gamma ^{S}_9 lagmax(1, t)$

All the notations are explained in Sects. 3.2 –3.5 and in Table 3 in the text. The covariates are explained in Table 1. The models were fitted using the SAS Procedure GLIMMIX (Supplementary Sects. S.4.4 and S.6). In the expressions given below, $\delta _{k}(t)$ represents the Dirac mass, i.e, $\delta _{k}(t)$ = 1 when *k* = *t* and 0 otherwise

The predation rates considered in this section are based on parameter tuning and are fixed in advance and not estimated by the state-space model during model fitting. The birth and survival rates are adjusted for environmental seasonality and seasonality in predation risk. During the dry season (July–October), migratory herbivores from the adjoining Serengeti Ecosystem in Tanzania occupy the Mara, generating a superabundance of food for large carnivores thereby considerably reducing predation risk for resident large herbivores, such as topi. So, we assume, based on parameter tuning, that predation risk for topi during the dry season is about 70% of the risk during the wet season when the migrants are absent from the Mara. Also, adult male topi often fight each other and defend mating territories (leks), potentially elevating their susceptibility to predation (Gosling 1991; Bro-Jørgensen and Durant 2003). Thus, we assume, also based on parameter tuning, that the survival rate for adult males is 99.7% that for adult females. We did a sensitivity analysis of the influence of age-specific predation risk on topi population dynamics captured by the state-space model. Details of how we represented predation risk and the sensitivity analysis are presented in Supplementary Sect. S.3.4.

### Observation Vector Construction and Modeling

We integrate the total population size estimates for the entire Masai Mara Ecosystem derived from the aerial surveys with the monthly demographic ground vehicle counts obtained primarily from the Masai Mara Reserve. This enables us to predict the total monthly population size estimates for the entire Masai Mara Ecosystem and for each of its four constituent zones (Masai Mara Reserve, Mara Conservancies, Loita Plains and Siana). Let $B_t = N(t) + Q(t) + H(t) + F(t) + M(t)$ be the true total topi population size for the Mara Reserve and $\mho _{t}$ be the true total topi population for all the four zones that collectively constitute the Mara Ecosystem. We further assume that $E(\mho _{t}) = K_t E(B_t)$, where $K_t > 1$ is the ratio of $E(\mho _{t})$ and $E(B_t)$ ($\mho _{t}$ is the population size for the entire Masai Mara ecosystem and $B_t$ is the population size for the Mara Reserve, one of the four zones making up the Masai Mara Ecosystem). Hence, $\mho _{t} > B_t$ for time *t* and similarly $E(\mho _{t}) > E(B_t)$. We assume $K_t$ = $\frac{\mho _{t}}{B_t}$ at time *t*. We integrate $\mho _{t}$ with the model for the ground counts as follows.

1 $$\begin{aligned} \lambda _t^{T}\sim & {} Gamma\left( \frac{(K_t B_t)^2}{\sigma _T^2}, \frac{K_t B_t}{\sigma _T^2}\right) \nonumber \\ \mho _{t}\sim & {} Poisson(\lambda _t^{T}) \end{aligned}$$

Suppose $\Psi _{t}$ is the estimated total topi population size for the entire Mara Ecosystem from the aerial survey at time *t* and has variance $\sigma _{\psi }^2$. We assume this estimate to be related to the true topi population size for the Mara Ecosystem at time *t*, $\mho _{t}$, as follows.

2 $$\begin{aligned} \Psi _{t}\mid \mho _{t} \sim N(\mho _{t}, \sigma _{\Psi }^2) \end{aligned}$$

The hidden (latent or true) states (*N*(*t*), *Q*(*t*), *H*(*t*), *F*(*t*), *M*(*t*)) are linked to the observed counts (*n*(*t*), *q*(*t*), *h*(*t*), *f*(*t*), *m*(*t*)) assuming Poisson distributions as described by the equations in Table 2. Note that the expected values of the observation vector components (observed counts) equal the corresponding state vector components (hidden, or true states), thus unbiased, with the notable exception of *n*(*t*).

The parameters $\sigma _{1}^2$, $\sigma _{2}^2$, $\sigma _{3}^2$, $\sigma _{4}^2$ and $\sigma _{5}^2$ in Table 2 are assumed to be random and are simulated using the MCMC method.

Our specifications in Eq. (2) and in Table 2 and use of the Poisson distribution imply that *n*(*t*) has the expected value *N*(*t*), that is, *E*[*n*(*t*)] = *N*(*t*). But some newborn topi are almost certainly missed during the ground surveys because topi hide their young for some time soon after birth, large carnivores kill some newborns, whereas others may simply be missed due to visibility bias. To account for potential underestimation, we multiplied *n*(*t*) with a correction factor of 1.7, based on experimentation, before determining birth rates in Sect. 3.5. More generally, however, sightability bias in *n*(*t*) can be modeled by allowing *n*(*t*) to follow a Poisson distribution with parameter $\frac{\lambda _1(t)}{h(t)}$, where *h*(*t*) is a proportionality factor. We do not expect the choice of this particular correction factor to unduly influence our results because this factor is used only to derive the initial parameter estimates in the logit regression models at the screening stage and not in the actual MCMC model fitting stage in Sect. 3.5.

### Specifying the Initial State Vector

We first clarify how we predict the expected number of animals in each age and sex class using simultaneous linear equations. Next, we describe how we initialize the population size for topi age and sex classes. We used an interdependent system of linear regression equations (Theil 1971) to estimate the expected total number of animals in each age and sex class present in the ground sample in each month for use in determining initial population size estimates. The current month endogenous (dependent or response) variables appear as regressors (covariates) in equations for other age or sex classes in the system of simultaneous equations. The model accounts for potential correlation of errors for the set of related regression equations to improve the efficiency of parameter estimates (Theil 1971). The modeling framework uses estimation procedures that produce consistent and asymptotically efficient estimates for the system of linear regression equations. We imposed linear restrictions on some of the parameter estimates (see SAS codes in Supplementary Sect. S.6). This procedure was only used to obtain initial parameter estimates for the logistic regression models at the screening stage and was not used during the actual model implementation using the MCMC method.

The initial states for different age and sex classes at time *t* = 0 (namely, $P(0, \ell )$, $1\le \ell \le 12$; *M*(0); *G*(0, *k*), $2\le k \le 19$ and *N*(0)) are assumed to follow normal distributions with means determined by the estimated population age and sex structure at the initial time *t* = 0 and variances assumed to be all equal to 20,000.

Running the MCMC chains requires specifying the starting values for population size at time *t* = 0 for all the age and sex classes. The specific initial state values chosen should not impact the final parameter estimates according to the theory of MCMC chains provided the chain is run a large enough number of times. We thus describe the procedure we used to choose the initial values of population size at *t* = 0 for the sake of completeness only. We used the aerial and ground survey data to estimate the unknown age and sex structure of the initial population at time *t* = 0 for the MCMC chains. First, we selected the ground sample counts for the month of June. We then calculated the proportion of animals in all the different age and sex classes in the month of June in each of the 15 years spanning 1989 to 2003 and averaged the proportion for each age and sex class across all the 15 years. We used this average to represent the population proportion for each age and sex class at time *t* = 0 for the month of June. To derive the initial population size estimate for each age and sex class, we multiplied the total population size estimated from the aerial survey data at time *t* = 0 (June 1989) with the average proportion for each age and sex class for June from the ground count data for July 1989 to December 2003. Further details of this exercise and and the pertinent equations are provided in Sect. S.2.3 of supplementary materials. We use these initial population size estimates for each age and sex class as the initial values for the MCMC chain. Note that we executed multiple MCMC chains, each with distinct initial value choices and obtained consistently similar results. Note also that although the initial population size estimates should be calculated using data outside the period of interest (July 1989 to December 2003) to avoid double use of the same data (Schaub and Kéry 2022), we estimated the initial topi age and sex structure for June 1989 from the ground count data for June in the study period because no other earlier data existed with topi age and sex structure matching that used in the ground counts.

We also use the initial population size estimates for each age and sex class as the means of the normal distributions for the corresponding initial states, *N*(0), *M*(0), etc.

## Model Fitting, Diagnostics, and Validation

There are various ways to initialize integrated state-space models, and various issues may arise when doing this. Besbeas and Morgan (2012) discuss these for models fitted using the Kalman filter.

### Prior Distributions

The prior distributions for the regression coefficients $\varvec{\gamma }^{S} = (\gamma ^{S}_1, \ldots , \gamma ^{S}_9)$, $\varvec{\gamma }^{R} = (\gamma ^{R}_1, \ldots , \gamma ^{R}_9)$, $\varvec{\gamma }^{Q} = (\gamma ^{Q}_1, \ldots , \gamma ^{Q}_{14})$, $\varvec{\gamma }^{H} = (\gamma ^{H}_1, \gamma ^{H}_2, \gamma ^{H}_3)$ and $\varvec{\gamma }^{A} = (\gamma ^{A}_1, \ldots , \gamma ^{A}_9)$ of the logit regression models are assumed to be normal with means determined as averages of simulated values from a pilot run and variance taken as 10,000. Note that these priors have very high variance and hence can be taken as un-informative priors. The empirical choice of the priors ensures good mixing of the MCMC chains. Note that a large variance in the normal prior for the coefficients of a logit regression model can lead to induced priors for the “probability” that are bathtub shaped (see Seaman III et al. (2012) for the problem statement and a “solution” in Newman (2003), equation 3.9, with variance $\frac{\pi ^2}{3(p+1)}$). Sensitivity analysis for such priors may thus be recommended.

We assumed a non-informative prior on $(0, \infty )$ for $\sigma _{i}^2$ in Sect. 3.6. Prior distributions for the initial states (*N*(0), *Q*(0, *k*), etc.) are specified in Sect. 3.7. The prior on $1/K_t$ is taken to be Beta(1, 1), which is also a non-informative prior. $\sigma _T^2$ and $\sigma _{\Psi }^2$ in Sect. 3.6 are also assumed to have non-informative uniform priors on $(0, \infty )$.

### Full Conditional Distributions, Full Likelihood and Convergence of MCMC Chains

The forms of the full conditional distributions of *N*(*t*), *Q*(*t*, *k*), *H*(*t*, *k*), $F_{a}(t)$, $M_{a}(t)$ and other parameters are presented in Supplementary Sect. S.2. The functional forms of the full conditional distributions of $\varvec{\gamma }^S$, $\varvec{\gamma }^R$, $\varvec{\gamma }^Q$, $\varvec{\gamma }^H$ and $\varvec{\gamma }^A$ (collectively referred to as $\varvec{\gamma }$’s) and other parameters are also presented in Supplementary Sect. S.2.2. The full likelihood is also provided in Supplementary Sect. S.2.1.

The functional forms of the full conditional distributions for the $\varvec{\gamma }$’s are not conformable to Gibbs sampling. Moreover, the resulting Metropolis–Hastings chain for the $\varvec{\gamma }$’s converges quite slowly, making the algorithm highly inefficient. To accelerate the rate of convergence of the chain, we implement the Transformation Markov Chain Monte Carlo (TMCMC) at the Metropolis–Hastings step. A theoretical discussion of TMCMC can be found in Dutta and Bhattacharya (2014). Details on how the TMCMC was specialized for our chain are discussed in Supplementary Sect. S.3.1. The MCMC simulations were continued for 3,000,000 iterations after discarding the initial 1,000,000 iterations as burn-in. The convergence of the MCMC chains for each parameter was assessed informally using trace plots in Supplementary Sect. S.3.1.

### Model Validation Using Balanced Bootstrap Sampling

To assess the robustness of the model, we performed a model validation test using balanced bootstrap samples. We first drew 10 different samples from each of the 75 aerial surveys conducted between 1977 and 2018 using balanced bootstrap sampling. The balanced bootstrap selection was performed by using the algorithm of Gleason (1988) in SAS PROC SURVEYSELECT (SAS Institute 2023). The balanced bootstrap method was used to select 10 samples from each of the total of 75 aerial surveys with equal probability and with replacement, where each aerial survey had 232 to 705 sampling units each measuring 5 km $\times$ 5 km, 2.5 km $\times$ 5 km or 10 km $\times$ 5 km. Because the bootstrap selection is balanced, the overall total number of selections is the same for each sampling unit (Davison et al. 1986). We then estimated the total topi population size for each bootstrap sample using Jolly’s Method 2 (Jolly 1969) for transects of unequal lengths. Further details on the balanced bootstrap sampling are provided in Supplementary Sect. S.3.2 and the SAS code for implementing the bootstrap in Sect. S.6.

### Cross-Validation Results

Before running the model to produce the parameter estimates and interpreting their significance, we validated the model to establish its suitability for predicting population dynamics. The estimated population sizes from the bootstrap samples served as the actual population size of a hypothetical topi population. Using these estimates, we generated a set of 10 time series of hypothetical ground survey data each of length 174 months and having the same age and sex classes as the actual ground sample count data (further details in Supplementary Sect. S.3.2). We call these hypothetical ground data sets generated data and the corresponding population size estimates bootstrap population estimates. We then fit our model separately to each of the generated data and obtained estimates of total population size and the corresponding 95% credible limits of the estimates from the state process model in Sect. 3.4. We call these estimates generated estimates. Next, we compare the bootstrap population estimates with the generated estimates by observing whether the bootstrap estimates lie within the 95% credible limits of the generated estimates for each of the 10 bootstrap population time series. However, running the model separately for each time series is computationally very expensive. To reduce the computing time, we used the idea behind the Importance Resampling MCMC (IRMCMC) proposed by Bhattacharya and Haslett (2007). Though developed for inverse problems, this method can be generalized to tackle the current problem. The precise details of this generalization are discussed in Supplementary Sect. S.3.3. We ran the model using IRMCMC for each of the generated data sets (10 time series) and calculated the percentage coverage of the bootstrap populations at each of the 174 time points. The coverages for the 10 time series of bootstrap samples vary from 85 to 97%. The bespoke R code written in R software version 4.1.1 used to implement the population dynamics model is provided in Supplementary Sect. S.6. Also, the SAS codes used to fit the simultaneous linear equations and relate birth recruitment and survival rates and adult sex ratio to the covariates, perform balanced bootstraping and estimate total population size using Jolly’s Method are also provided in Supplementary Sect. S.6.

To assess the convergence of the MCMC chains and the influence of starting values, we ran multiple chains with varied initial values and calculated ${\hat{R}}$ values. Given the substantial storage requirements for running the chains multiple times and storing the necessary data for calculating ${\hat{R}}$, which led to memory issues on our computers, we limited each chain to 200,000 simulations, after discarding the first 200,000 samples as burn-in. We used three distinct starting values for each of these runs. For each combination of chain run and starting value, we computed ${\hat{R}}$ and consistently found it to be very close to 1. This consistency led us to conclude that the chains had successfully converged and that the starting values had no undue influence on convergence. To quantify the degree of autocorrelation of the chains, we calculated the effective sample sizes of the MCMC simulations for ten randomly selected parameters using a subsample of 30,000 and found the effective sample sizes for all the parameters to range between 1250 and 5000. This suggests satisfactorily effective sample size for each parameter for the full 3,000,000 samples (see Sect. S.3.1 in Supplementary materials). We have also adopted a pragmatic approach to assess the identifiability of our model. Typically, non-identifiable models are characterized by multimodal posterior densities. However, in our analysis, none of the posterior densities for the model parameters exhibited multimodality. Moreover, flat likelihood functions can indicate that the data has limited or no impact on the posterior, and hence non-identifiability. A prior that matches the posterior distribution typically indicates that the data had no influence on the parameter estimation and that the parameter is not estimable. To check for parameter estimability, we conducted Kolmogorov–Smirnov tests for the prior and posterior densities. These confirmed that the two densities were different for all the parameters. This suggests that the priors did not have strong influences on the posterior distributions and that the data had a significant influence and hence there is no indication of identifiability issues and that the parameters are identifiable and estimable (Sect. S.3.1 in Supplementary materials). High collinearity in a joint posterior, ridges in a 2-D contour of the likelihood, or relatively flat likelihood functions may reflect “weak” identifiability. So, we computed the degree of correlation among the marginal posterior distributions for the unobserved states (absolute value < 0.57) and the parameters (absolute value < 0.17 (except for birth recruitment for which three parameters had moderate correlation: 0.62$-$0.83) and found them to be relatively modest, providing further evidence of reasonable model fit. Lastly, we computed correlations between the parameters and the states. These showed that the correlations between the parameters and the states for time periods 1 to 174 lie between -$-$0.48 and 0.75 for newborns; -$-$0.26 and 0.49 for quarters; -$-$0.069 and 0.44 for half-yearlings; -$-$0.56 and 0.05 for adult females; and -$-$0.41 and 0.014 for adult males, implying that the parameter estimates are only weakly associated with the states and hence providing little evidence of non-identifiability.

## Results

### Topi Population Trajectory by Age and Sex Class

**Fig. 2:**
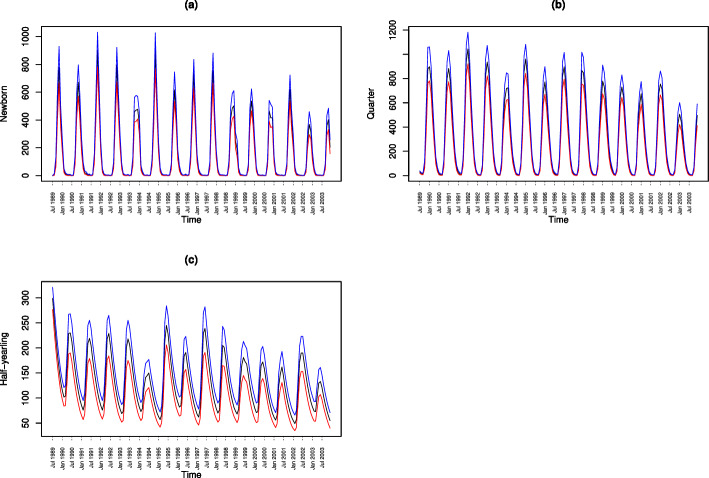
Modeled (black lines) population trajectories (posterior means) and the associated 95% lower (red lines) and upper (blue lines) credible limits for **a** newborn (*N*(*t*)), **b** quarter (*Q*(*t*)) and **c** half-yearling (*H*(*t*)) topi (Color figure online)

**Fig. 3:**
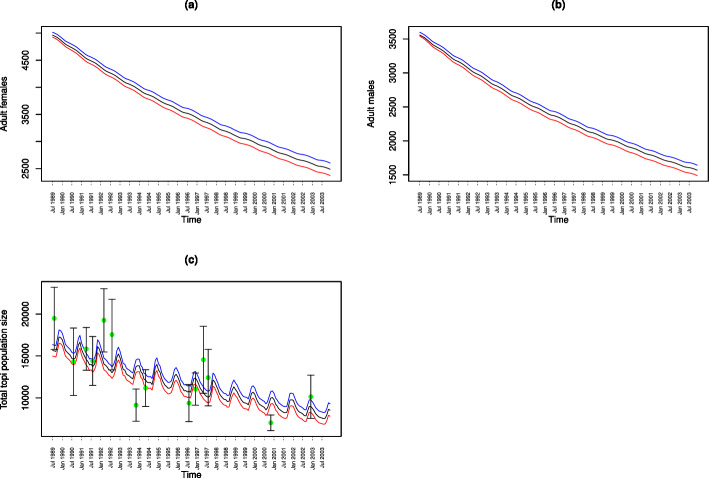
Modeled (black lines) population trajectories (posterior means) and the associated 95% lower (red line) and upper (blue line) credible limits for **a** adult female (*F*(*t*)), **b** adult male (*M*(*t*)) and **c** total topi population size (*N*(*t*)+*Q*(*t*)+*H*(*t*)+*F*(*t*)+*M*(*t*)) overlaid with the population size estimates (green filled circles) and their standard errors (vertical whiskers) based on the Directorate of Resource Surveys and Remote Sensing (DRSRS) aerial surveys. The DRSRS population size estimates and their standard errors were multiplied by 1.33 to correct for sightability bias (Stelfox et al. 1986) (Color figure online)

The model captures the essential and well-known features of the Mara topi population dynamics. First, it accurately captures the declining population trajectory of all the age and sex classes: adult female, adult male, half-yearling, quarter size and newborn topi in the Mara between 1989 and 2003 (Figs. 2 and 3). Second, it accurately tracks inter-annual variation in the reproductive seasonality, phenology, synchrony and prolificity of topi births and juvenile recruitment (Fig. 2; Sinclair et al. 2000; Ogutu et al. 2010, 2011). Figures 2 and 3 show the observed (only Fig. 3c) and predicted population sizes for each age and sex class for each of the 174 sampling points (July 1989–December 2003) and the associated 95% credible limits averaged across the 3,000,000 MCMC simulation samples. The 95% credible interval widths relative to the posterior mean values are 27.7–500% for newborns, 23.5–300% for quarters, 14.7-$-$63.3% for half-yearlings, 1.8-$-$9.3% for adult females, 1.6-$-$9.7% for adult males, and 8.7-$-$18.6% for the total population, indicating greater uncertainty in the predicted population sizes for juvenile topi. The birth recruitment rates (Figs. 2 and S18a) show strong seasonality consistent with the pronounced reproductive seasonality characteristic of the Mara-Serengeti topi. Further, the prolificacy of topi births is strongly time-varying, reflecting the controlling influence of the seasonally and inter-annually varying rainfall (Ogutu et al. 2010, 2014a). The pronounced seasonality in prolificacy of births and birth recruitment also carry over to the trajectories of the quarter and half-yearling size classes (Fig. 2b–c) but not to the adult age class (Fig. 2a–b). The persistent declines in the trajectories of topi birth recruitment, quarter and half-yearling classes, adult males and females and the overall topi population size are consistent with the overall topi population decline in the Mara Ecosystem from 1977 to 2018 (Fig. 3c; Ogutu et al. 2016; Veldhuis et al. 2019). Finally, there is evident seasonality in the overall topi population trajectory generated by the strong seasonality of births and juvenile recruitment in the ecosystem (Fig. 3c; Ogutu et al. 2011, 2014a).

Younger topi age classes show distinct seasonality in numbers because of strong birth seasonality. This ripples through the population to quarters and half-yearlings. However, adult topi show no seasonality in numbers, likely because only a very small proportion of newborns survive to adulthood (Fig. S24). Consequently, these survivors have minimal impact on the overall adult population size in any given month.

We also studied cohort-specific trajectories, ages 0 to 19 months, for multiple cohorts to see the impact of survival rates on decline of cohort numbers (see Sect. S.4.6 in Supplementary materials).

### Adult Female Recruitment and Females Available to Conceive

Adult female recruitment is strongly seasonal, consistent with the seasonality in births and juvenile recruitment. The expected number of new adult females recruited into the population per month peaked in 1989–1990, 1996–1997, 2002–2003 and were noticeably higher than other years (Fig. S16a in Supplementary Sect. S.4.1). Moreover, the number of adult females that gave birth exactly 8 months ago (Fig. S16b) and the number of adult females that were available to conceive (Fig. S16c) decreased persistently and markedly. The latter reduced from a maximum of nearly 4000 in 1989 to around 2000 animals by 2003 (Fig. S16c). Thus, the decline in newborns was associated with a persistent decline in the number of adult females. Notably also, the severe drought in 1993 resulted in evidently fewer adult females (in 1994) that gave birth in 1993 (Fig. S16b) or that were available to conceive in 1994–1995 (Fig. S16c).

### Temporal Variation in Age Structure and Adult Sex Ratio

The expected population proportions of newborns, quarter size, half-yearlings and adults all tended to remain stable on average as the topi population decreased. All the population components show sustained seasonal oscillations from 1989 to 2003 (Figs. S17a–d in Supplementary Sect. S.4.2). However, the lower limits of the oscillations show decreasing trend.

### Factors Influencing Birth Recruitment and Survival Rates and Adult Sex Ratio

The posterior means, standard deviations and 95% credible intervals for parameters of the models relating birth and adult recruitments, survival rates and adult sex ratio to various covariates are summarized in Tables S5–9 in Supplementary Sect. S.4.5. Similarly, posterior densities for a sample of the parameters are displayed in Figs. S20–23. There was evident seasonality in birth recruitment and survival of quarter size, half-yearling and adult topi (Figs. S18a-d in Supplementary Sect. S.4.3). Birth recruitment was negatively density-dependent for the declining topi population and was also influenced by prior rainfall and average temperatures (Table S5). Besides seasonality, quarter size survival was influenced only by prior rainfall (Table S6). Half-yearling survival fluctuated seasonally and was apparently influenced only by past rainfall (Table S7). Adult survival was negatively density dependent and also varied with past rainfall amounts (Table S8). Lastly, adult sex ratio varied seasonally and with rainfall, minimum and maximum temperatures but was apparently not density-dependent (Table S9). The simple and informal sensitivity assessment showed the total topi population size to be highly sensitive to changes in the predation risk for the adult age class but to be much less sensitive to the same amount of change in predation risk for the newborn, quarter or half-yearling age class (Fig. S15 in Supplementary Sect. S.3.4).

### Birth Recruitment Rates, Survival Rates and Sex Ratio

The estimated birth recruitment, survival rates and sex ratios are consistent with expectation and are displayed in Figs. S18–19 in Supplementary Sect. S.4.3. Topi birth recruitment was strongly seasonal, unusually low during the 1993–1994 drought and the highest during 1994–1995 with good rainfall (Fig. S18a). The survival rates for the quarter size (Fig. S18b), half-yearling (Fig. S18c) and adult (Fig. S13d) topi age classes showed strong and sustained seasonality. Adult survival was the lowest during 1999–2001, coincident with the extreme 1999–2000 drought (Fig. S18d). The proportion of adult females increased from a monthly minimum of 58 to 63%, whereas the proportion of adult males decreased correspondingly from a peak of about 42 to 37% between 1989 and 2003 (Fig. S19a–b).

Despite significant variation in rainfall during their birth year, seven hypothetical cohorts of 3500 topi each, born in normal, mild, severe and extreme drought years, and extremely wet years, contributed a comparable, strikingly low number of recruits to the adult age class 19 months later. This indicates, surprisingly, extremely low survival and recruitment rates across all conditions (Fig. S24).

### Comparing Predicted Topi Population Size with Aerial Survey Data

Fourteen of the 75 DRSRS aerial surveys for the Masai Mara Ecosystem for 1977–2018 (Sect. 2.2) fell within the period spanned by the ground survey data (July 1989–December 2003). The total population size estimates from these 14 aerial surveys were in reasonable agreement with the total topi population size predicted by the Bayesian state-space model. Notably, the estimated total topi population size was within the 95% confidence limits of most of the total population size estimates derived from the 14 DRSRS aerial surveys (Fig. 3). Moreover, trends in the estimated topi population size for each of the four zones constituting the Mara ecosystem (Fig. S25a–d in Supplementary Sect. S.4.7) are also in good agreement with the corresponding aerial total population size estimates and show that topi numbers worryingly declined persistently throughout the Mara Ecosystem regardless of the degree of protection—the highest in the Mara Reserve, intermediate in the Mara conservancies and the lowest in the unprotected agro-pastoral lands of Siana and the Loita Plains (also see Figs. S11 and S12). However, topi were substantially more abundant in the protected reserve and semi-protected conservancies than in the unprotected agro-pastoral lands. This model can therefore be used to rigorously assess management effectiveness, in particular the effects of protection on populations.

## Discussion

We develop a flexible and general, integrated state-space model in a Bayesian framework for estimating large herbivore population demographic parameters, dynamics and the associated uncertainties. The model is illustrated using the Mara-Serengeti topi population inhabiting the World-famous Greater Mara-Serengeti Ecosystem of Kenya and Tanzania in East Africa. The state-space model allows estimation of both process and observation error variances (De Valpine and Hastings 2002; Buckland et al. 2004). The model incorporates age and sex structure and key life history characteristics (e.g., gestation length, lactation period, pregnancy status, females available to conceive, number of young per birth event) and life history strategies (e.g., feeding style, grazer for topi, and foraging style, resident for topi) essential to understanding large herbivore population dynamics and efficiently integrates ground demographic survey with aerial survey data. We relate birth and adult recruitments and age class specific survival rates and adult sex ratio to various covariates, such as prior population density, predation risk, past rainfall and temperature, environmental seasonality and their interactions. To estimate model parameters, we used the MCMC method in a Bayesian framework because of its flexibility (Brooks et al. 2004; Hoyle and Maunder 2004; Schaub et al. 2007; Finke et al. 2019). The convergence of the MCMC chains of the parameters is accelerated using the Transformation MCMC (TMCMC) technique (Dutta and Bhattacharya 2014) and the idea behind the IRMCMC (Bhattacharya and Haslett 2007) to reduce the computational time for model validation.

The predicted population trajectories show persistent and marked declines in the Mara topi population from 1989 to 2003 in accord with the trends derived from aerial surveys for 1977–2018 (Ogutu et al. 2016; Veldhuis et al. 2019) and for 1977–2022 (Figs. S11 and S12). Importantly, whereas birth and adult recruitments fluctuated around a stable average, the survival rates for quarter size, half-yearling size and adults decreased gradually and contemporaneously with the overall topi population decline. The disturbing and sustained decline in numbers of topi and other species in the Mara and across Kenya (Ogutu et al. 2016; Figs. S10 and S11) and continental Africa (Craigie et al. 2010) increases the urgency for establishing their leading causes and developing effective population conservation and recovery strategies.

The modeling framework can be extended to (i) identify the most influential processes driving population declines and assess their relative importance, (ii) test interesting ecological hypotheses, (iii) enable rigorous forward projection of large herbivore population dynamics allowing for both parameter and future demographic uncertainty (Hoyle and Maunder (2004), Figs. S10 and S11). Further extension can also (iv) enable assessment of likely future population trajectories under various scenarios of climate change, land use change, socio-economic development, human population growth, livestock population density, conservation and management interventions, such as formation of new wildlife conservancies (Rhodes et al. 2011; Maunder et al. 2015; Mosnier et al. 2015). Moreover, the monthly time step allows the model outputs to be used to study potential shifts in reproductive seasonality, phenology, synchrony and prolificacy of births, juvenile recruitment and adult sex ratio (Ogutu et al. 2010, 2011, 2014b) in response to future changes in climate and other factors. It would also be interesting and useful to adapt the model for other species, with contrasting life history traits and strategies as well as trophic relations, such as hartebeest (*Alcelaphus buselaphus cokeii*), impala (*Aepyceros melampus*), waterbuck (*Kobus ellpsyprimnus*), zebra, warthog (*Pharcocoerus africana*) and giraffe (*Giraffa camelopardalis*). We are already working on these extensions.

The model can also be extended to include several additional features. First, females that are reproducing for the first time can be allowed to have a lower pregnancy rate (86%) than older (100%) females (Duncan 1975). Second, females that lose their calves soon after birth can be moved to the group of females available to conceive. Third, the survival rate of calves aged 0 to 1 month can be explicitly incorporated in the model, especially if empirical estimates of such rates can be obtained. Fourth, the dependence of birth recruitment and age-specific survival rates and sex ratio on covariates may alternatively be made time varying to potentially better account for temporal variation in the influence of the covariates. Fifth, the model can be made spatially explicit to allow for spatial variation in the type and intensity of factors influencing survival and recruitment rates and sex ratio. Sixth, sightability bias in newborns, *n*(*t*), can be modeled explicitly by specifying *n*(*t*) to follow a Poisson distribution with parameter $\lambda _1(t)/h(t)$, where the proportionality constant *h*(*t*) is sampled from a suitable probability distribution. Seventh, birth recruitment and survival rates and sex ratio can be related to additional covariates, particularly anthropogenic covariates, such as human population density, livestock population density, settlement density and progressive habitat loss, such as that due to proliferation of fences. Lastly, birth recruitment, survival rates and sex ratio can all be related to one set of covariates collected at the monthly time scale using the ground demographic data, whereas the inter-annual variation in the overall population growth rate can be related to the other set of covariates collected at the annual time scale using the aerial survey data.

We are already working on developing these extensions for the six large herbivore species monitored alongside topi by both the ground and aerial sample counts in the Greater Mara-Serengeti Ecosystem. In these extensions, we aim to formalize SSMs model fitting and assessment (Auger-Méthé et al. 2021; Newman et al. 2023), as much as possible by (a) trying different probability distributions in the state process and observation models, e.g., lognormal, overinflated Poisson or negative binomial for count data; (b) performing formalized model selection, e.g., by computing and comparing the Watanabe Information Criterion (Watanabe 2013); and extensively exploring (c) model goodness of fit and (d) model redundancy, estimability and identifiability, which can often pose problems with SSMs (Cole and McCrea 2016; Cole 2020), as well as (e) degree of correlation among the posterior distributions for the unobserved states, the parameters and between states and parameters. Multi-modality in the posteriors, high collinearity in a joint posterior, ridges in a 2-D contour of the likelihood, or relatively flat likelihood functions are all signs of “weak” identifiability. We also explore the stability of the MCMC chains by running the chains using multiple sets of initial values for the population sizes of different age and sex classes. Lastly, we are exploring the actual performance of a fitted linear Gaussian approximation to the state-space model, using the Kalman filter to calculate maximum likelihood estimates in a sequel to this paper.

## Supplementary Information

Below is the link to the electronic supplementary material.

Supplementary file 1 (zip 422 KB)

Supplementary file 2 (r 163 KB)

Supplementary file 3 (r 148 KB)

Supplementary file 4 (zip 2373 KB)

Supplementary file 5 (zip 1301 KB)

Supplementary file 6 (zip 83 KB)

Supplementary file 7 (pdf 211540 KB)

## References

[CR1] Auger-Méthé M, Newman K, Cole D, Empacher F, Gryba R, King AA, Leos-Barajas V, Mills Flemming J, Nielsen A, Petris G (2021) A guide to state-space modeling of ecological time series. Ecol Monogr 91(4):e01470

[CR2] Bartzke GS, Ogutu JO, Mukhopadhyay S, Mtui D, Dublin HT, Piepho HP (2018) Rainfall trends and variation in the Maasai Mara ecosystem and their implications for animal population and biodiversity dynamics. PLoS ONE 13(9):e0202814 30231048 10.1371/journal.pone.0202814 PMC6145597

[CR3] Besbeas P, Freeman SN, Morgan BJ (2005) The potential of integrated population modelling. Aust N Z J Stat 47(1):35–48

[CR4] Besbeas P, Morgan BJ (2012) Kalman filter initialization for integrated population modelling. J R Stat Soc Ser C 61(1):151–162

[CR5] Bhattacharya S, Haslett H (2007) Importance re-sampling MCMC for cross-Validation in inverse problems. Bayesian Anal 2(2):385–408

[CR6] Boutton TW, Tieszen LL, Imbamba SK (1988) Biomass dynamics of grassland vegetation in Kenya. Afr J Ecol 26(2):89–101

[CR7] Boutton TW, Tieszen LL, Imbamba SK (1988) Seasonal changes in the nutrient content of East African grassland vegetation. Afr J Ecol 26(2):103–115

[CR8] Bro-Jørgensen J, Durant SM (2003) Mating strategies of topi bulls: getting in the centre of attention. Anim Behav 65(3):585–594

[CR9] Brooks SP, King R, Morgan BJT (2004) A Bayesian approach to combining animal abundance and demographic data. Anim Biodivers Conserv 27(1):515–529

[CR10] Buckland ST, Newman KB, Fernández C, Thomas L, Harwood J (2007) Embedding population dynamics models in inference. Stat Sci 22(1):44–58

[CR11] Buckland ST, Newman KB, Thomas L, Koesters NB (2004) State-space models for the dynamics of wild animal populations. Ecol Model 171(2):157–175

[CR12] Caswell H (2000) Matrix population models, vol 1. Sinauer Sunderland, Sunderland, MA

[CR13] Ceballos G, Ehrlich PR, Dirzo R (2017) Biological annihilation via the ongoing sixth mass extinction signaled by vertebrate population losses and declines. Proc Natl Acad of Sci 114(30):E6089–E6096 28696295 10.1073/pnas.1704949114 PMC5544311

[CR14] Chase MJ, Schlossberg S, Griffin CR, Bouché PJC, Djene SW, Elkan PW, Ferreira S, Grossman F, Kohi EM, Landen K, Omondi P, Peltier A, Selier SAJ, Sutcliffe R (2016) Continent-wide survey reveals massive decline in African savannah elephants. PeerJ 4:e2354 27635327 10.7717/peerj.2354 PMC5012305

[CR15] Cole D (2020) Parameter redundancy and identifiability. CRC Press, Boca Raton

[CR16] Cole DJ, McCrea RS (2016) Parameter redundancy in discrete state-space and integrated models. Biom J 58(5):1071–1090 27362826 10.1002/bimj.201400239 PMC5031231

[CR17] Craigie ID, Baillie EM, Balmford A, Carbone C, Collen B, Green RE, Hutton JM (2010) Large mammal population declines in Africa’s protected areas. Biol Cons 143(9):2221–2228

[CR18] Davison AC, Hinkley DV, Schechtman E (1986) Algorithms for balanced bootstrap simulations. Biometrika 73:555–566

[CR19] De Valpine P, Hastings A (2002) Fitting population models incorporating process noise and observation error. Ecol Monogr 72(1):57–76

[CR20] Deshmukh IK (1984) A common relationship between precipitation and grassland peak biomass for east and southern Africa. Afr J Ecol 22:181–186

[CR21] Duncan P (1975) Topi and their food supply. University of Nairobi, Nairobi, Kenya

[CR22] Dutta S, Bhattacharya S (2014) Markov chain Monte Carlo based on deterministic transformations. Stat Methodol 16:100–116

[CR23] Estes RD (1991) The behavior guide to African mammals: including hoofed mammals. University of California Press, Carnivores, Primates, California

[CR24] Finke A, King R, Beskos A, Dellaportas P (2019) Efficient sequential Monte Carlo algorithms for integrated population models. J Agric Biol Environ Stat 24(2):204–224

[CR25] Funk C, Peterson P, Landsfeld M, Pedreros D, Vermin J, Shukla S, Husak G, Rowland J, Harrison L, Hoell A, Michaelsen J (2015) The climate hazards infrared precipitation with stations–a new environmental record for monitoring extremes. Sci Data 2:1–21. 10.1038/sdata.2015.66 10.1038/sdata.2015.66 PMC4672685 26646728

[CR26] Gleason JR (1988) Algorithms for balanced bootstrap simulations. Am Stat 42:263–266

[CR27] Gosling LM (1991) The alternative mating strategies of male topi, *Damaliscus lunatus*. Appl Anim Behav Sci 29:107–119

[CR28] Hoyle SD, Maunder MN (2004) A Bayesian integrated population dynamics model to analyze data for protected species. Anim Biodivers Conserv 27(1):247–266

[CR29] Iijima H, Nagaike T, Honda T (2013) Estimation of deer population dynamics using a Bayesian state-space model with multiple abundance indices. J Wildl Manag 77(5):1038–1047

[CR30] Jolly GM (1969) Sampling methods for aerial censuses of wildlife populations. East Afr Agric For J 34:46–49

[CR31] Maunder MN, Deriso RB, Hanson CH (2015) Use of state-space population dynamics models in hypothesis testing: advantages over simple log-linear regressions for modeling survival, illustrated with application to longfin smelt (*Spirinchus thaleichthys*). Fish Res 164:102–111

[CR32] McCallum ML (2015) Vertebrate biodiversity losses point to a sixth mass extinction. Biodivers Conserv 24:2497–2519

[CR33] Mosnier A, Doniol-Valcroze T, Gosselin JF, Lesage V, Measures LN, Hammill MO (2015) Insights into processes of population decline using an integrated population model: the case of the St. Lawrence Estuary beluga (*Delphinapterus leucas*). Ecol Model 314:15–31

[CR34] Mukhopadhyay S, Ogutu JO, Bartzke GS, Dublin HT, Piepho HP (2019) Modelling spatio-temporal variation in sparse rainfall data using a hierarchical Bayesian regression model. J Agric Biol Environ Stat 24:369–393

[CR35] Nadeem K, Moore JE, Zhang Y, Chipman H (2016) Integrating population dynamics models and distance sampling data: a spatial hierarchical state-space approach. Ecology 97(7):1735–1745 27859153 10.1890/15-1406.1

[CR36] Newman K (2003) Modelling paired release-recovery data in the presence of survival and capture heterogeneity with application to marked juvenile salmon. Stat Model 3(3):157–177

[CR37] Newman KB, Buckland ST, Morgan BJ, King R, Borchers DL, Cole DJ, Besbeas P, Gimenez O, Thomas L (2014) Modelling population dynamics. Springer, New York, NY, USA

[CR38] Newman KB, Buckland TB, Lindley ST, Thomas L, Fernández CF (2006) Hidden process models for animal population dynamics. Ecol Appl 16(1):74–86 16705962 10.1890/04-0592

[CR39] Newman KB, Fernández C, Thomas L, Buckland ST (2009) Monte Carlo inference for state-space models of wild animal populations. Biometrics 65(2):572–583 18565166 10.1111/j.1541-0420.2008.01073.x

[CR40] Newman KB, Lindley ST (2006) Accounting for demographic and environmental stochasticity, observation error, and parameter uncertainty in fish population dynamics models. Am J Fish Manag 26(3):685–701

[CR41] Newman K, King R, Elvira V, de Valpine P, McCrea RS, Morgan BJ (2023) State-space models for ecological time-series data: practical model-fitting. Methods Ecol Evol 14(1):26–42

[CR42] Ogutu JO, Piepho HP, Dublin HT (2014) Reproductive seasonality in African ungulates in relation to rainfall. Wildl Res 41:323–342

[CR43] Ogutu JO, Piepho HP, Dublin HT, Bhola N, Reid RS (2008) El Niño Southern oscillation, rainfall, temperature and normalized difference vegetation index fluctuations in the Mara-Serengeti ecosystem. Afr J Ecol 46:132–143

[CR44] Ogutu JO, Piepho HP, Dublin HT, Bhola N, Reid RS (2008) Rainfall influences on ungulate population abundance in the Mara-Serengeti ecosystem. J Anim Ecol 77:814–829 18422558 10.1111/j.1365-2656.2008.01392.x

[CR45] Ogutu JO, Piepho HP, Dublin HT, Bhola N, Reid RS (2010) Rainfall extremes explain interannual shifts in timing and synchrony of calving in topi and warthog. Popul Ecol 52:89–102

[CR46] Ogutu JO, Piepho HP, Dublin HT, Bhola N, Reid RS (2011) Dynamics of births and juvenile recruitment in Mara-Serengeti ungulates in relation to climatic and land use changes. Popul Ecol 53(1):195–213

[CR47] Ogutu JO, Piepho HP, Said MY, Ojwang GO, Njino LW, Kifugo SC, Wargute PW (2016) Extreme wildlife declines and concurrent increase in livestock numbers in Kenya: what are the causes? PLoS ONE 11(9):e0163249 27676077 10.1371/journal.pone.0163249 PMC5039022

[CR48] Ogutu J, Piepho H, Dublin H (2014) Responses of phenology, synchrony and fecundity of breeding by African ungulates to interannual variation in rainfall. Wildl Res 40(8):698–717

[CR49] Polansky L, Newman KB, Mitchell L (2021) Improving inference for nonlinear state-space models of animal population dynamics given biased sequential life stage data. Biometrics 77(1):352–361 32243577 10.1111/biom.13267 PMC7984174

[CR50] Rhodes JR, Ng CF, de Villiers D, Preece HJ, McAlpine CA, Possingham HP (2011) Using integrated population modelling to quantify the implications of multiple threatening processes for a rapidly declining population. Biol Conserv 144:1081–1088

[CR51] SAS Institute (2023) System for statistical analysis, version 9.4. SAS Institute Inc., Carey, NC

[CR52] Schaub M, Gimenez O, Sierro A, Arlettaz R (2007) Use of integrated modeling to enhance estimates of population dynamics obtained from limited data. Conserv Biol 21(4):945–955 17650245 10.1111/j.1523-1739.2007.00743.x

[CR53] Schaub M, Kéry M (2022) Integrated population models: theory and ecological applications with R and JAGS. Academic Press, London

[CR54] Seaman III JW, Seaman JW Jr, Stamey JD (2012) Hidden dangers of specifying noninformative priors. Am Stat 66(2):77–84

[CR55] Sinclair ARE, Mduma SA, Arcese P (2000) What determines phenology and synchrony of ungulate breeding in Serengeti? Ecology 81:2100–2111

[CR56] Skinner JD, Chimimba CT (2005) The mammals of the southern African subregion. Cambridge University Press, Cambridge

[CR57] Stelfox JG, Peden DG, Epp H, Hudson RJ, Mbugua SW, Agatsiva JL, Amuyunzu CL (1986) Herbivore dynamics in southern Narok, Kenya. J Wildl Manag 50(2):339–347

[CR58] Theil H (1971) Principles of econometrics. Wiley, New York

[CR59] Thomas L, Buckland ST, Newman KB, Harwood J (2005) A unified framework for modelling wildlife population dynamics. Aust N Z J Stat 47(1):19–34

[CR60] Trenkel VM, Elston DA, Buckland ST (2000) Fitting population dynamics models to count and cull data using sequential importance sampling. J Am Stat Assoc 95(450):363–374

[CR61] Veldhuis MP, Ritchie ME, Ogutu JO, Morrison TA, Beale CM, Estes AB, Wargute PW (2019) Cross-boundary human impacts compromise the Serengeti-Mara ecosystem. Science 363(6434):1424–1428 30923217 10.1126/science.aav0564

[CR62] Vesey-Fitz-Gerald DF (1955) The topi herd. Oryx 3(1):4–8

[CR63] Watanabe S (2013) A widely applicable Bayesian information criterion. J Mach Learn Res 14(1):867–897

[CR64] Zhao Q, Arnold TW, Devries JH, Howerter DW, Clark RG, Weegman MD (2019a) Land-use change increases climatic vulnerability of migratory birds: insights from integrated population modelling. J Anim Ecol 88(10):1625–1637 10.1111/1365-2656.13043 31173349

[CR65] Zhao Q, Boomer GS, Royle JA (2019b) Integrated modeling predicts shifts in waterbird population dynamics under climate change. Ecography 42(9):1470–1481

