# Supplementary material for: Hierarchical Bayesian Integrated Modeling of Age- and Sex-Structured Wildlife Population Dynamics
Source: J Agric Biol Environ Stat. 2024 Jul 2;30(4):1068–93. doi: 10.1007/s13253-024-00634-w (PMC12559060; doi:10.1007/s13253-024-00634-w)
Supplement: Supplementary file 4 — (zip 2373 KB) [file 13253_2024_634_MOESM4_ESM.zip › Data_Dictionary_New/Data_Dictionary.docx]

**Data dictionary**

**File name: rainfall_mav_new.txt**

| **Number** | **Variable name** | **Description** |
| --- | --- | --- |
| 1 | year | Calendar year |
| 2 | month | Calendar month |
| 3 | rain | Total monthly rainfall in mm |
| 4 | lagrain0-lagrain12 | Total monthly rainfall in mm lagged by 0 to 12 months |
| 5 | dry1 | Total dry season (July-October) rainfall in mm lagged by 1 year. |
| 6 | wet1 | Total wet season (November-June) rainfall in mm lagged by 1 year. The wet season starts in November of the previous year and ends in June of the current year |
| 7 | annual1 | Total annual (November-October) rainfall in mm lagged by 1 year. The climatological year starts in November of the previous year and ends in October of the current year |
| 8 | earlywet1 | Total early wet season (November-February) rainfall in mm lagged by 1 year |
| 9 | latewet1 | Total late wet season (March-June) rainfall in mm lagged by 1 year |
| 10 | mavlatewet2 | 2 year moving (running) average of the total late wet (March-June) season rainfall |
| 11 | mavlatewet3 | 3 year moving (running) average of the total late wet season (March-June) rainfall in mm lagged by 3 years |
| 12 | earlydry1 | Total early dry season (July-August) rainfall in mm lagged by 1 year |
| 13 | earlydry3 | Total early dry season (July-August) rainfall in mm lagged by 3 years |
| 14 | latedry1 | Total late dry season (September-October) rainfall in mm lagged by 1 year |
| 15 | mavearlydry3 | 3-year moving (running) average of the total early dry  season (July-August) rainfall in mm |
| 16 | newborn_rain  =rain7-11 | The running average of the total monthly rainfall in mm lagged by 6 to 10 months =(lagrain6+lagrain7+lagrain8+lagrain9+lagrain10)/5 |
| 17 | half_rain= mavrain3_7 | The running average of the total monthly rainfall in mm lagged by 3 to 7 months = (Lagrain3+lagrain4+Lagrain5+lagrain6+lagrain7)/5 |

**File name: Predicted3.csv**

| **Number** | **Variable name** | **Description** |
| --- | --- | --- |
| 1 | Year | Calendar year (1989 to 2003) |
| 2 | Month 2 | Calendar month (January to December) |
| 3 | pred | Estimated total population size from the aerial surveys |
| 4 | lower | Lower bound of the estimated total population size from the aerial surveys |
| 5 | upper | Upper bound of the estimated total population size from the aerial surveys |
| 6 | lag3 | Total monthly rainfall in mm /85.66922807 lagged by 3 months |
| 7 | lag4 | Total monthly rainfall in mm /85.66922807 lagged by 4 months |
| 8 | lag5 | Total monthly rainfall in mm /85.66922807 lagged by 4 months |
| 9 | lag6 | Total monthly rainfall in mm /85.66922807 lagged by 4 months |
| 10 | lag7 | Total monthly rainfall in mm lagged 85.66922807 lagged by 4 months |
| 11 | dry1 | Total dry season (July-October) rainfall in mm /220.5998479 lagged by 1 year |
| 12 | D1 | Estimate of the total population size from aerial surveys lagged by 1 month. Population size estimates  were interpolated to cover all months between July 1989 and December 2003 |

**File name: Predicted5.csv**

| **Number** | **Variable name** | **Description** |
| --- | --- | --- |
| 1 | Year | Calendar year (1989 to 2003) |
| 2 | Month2 | Calendar month (1=January, 12=December) |
| 3 | Month3 | Calendar month with (1=July,2= August,…,June) |
| 4 | Min | Average monthly minimum temperature (°C) for the  current month |
| 5 | Max | Average monthly maximum for the  current month |
| 6 | max_3 | Average monthly maximum temperature (°C) lagged by 3 months |
| 7 | max_4 | Average monthly maximum temperature (°C) lagged by 4 months |
| 8 | min_5 | Average monthly minimum temperature (°C) lagged by 5 months |

**File name: eigen_values_rho_new3.txt**

| **Number** | **Variable name** | **Description** |
| --- | --- | --- |
| 1 | New | Parameters for the regression coefficients of birth recruitment computed by the TMCMC method |

**File name: eigen_values1.csv**

| **Number** | **Variable name** | **Description** |
| --- | --- | --- |
| 1 | NA | Parameters for regression coefficients for the quarter age class computed by the TMCMC method |
| 2 | NA | Parameters for regression coefficients for the half-yearling age class computed by the TMCMC method |
| 3 | NA | Parameters for regression coefficients for the quarter age class computed by the TMCMC method |

**File name: TopiData.csv**

| **Number** | **Variable name** | **Description** |
| --- | --- | --- |
| 1 | Year | Calendar year (1989 to 2003) |
| 2 | Month | Calendar month with entries as January,…,December |
| 3 | Month2 | Calendar month (1=January,…,12= December) |
| 4 | Species | Species name (=Topi) |
| 5 | New | Total ground sample count of number of newborn  calves |
| 6 | Quarter | Total ground sample count of quarter-  size juveniles |
| 7 | Hyearling | Total ground sample count of half-yearling age class |
| 8 | Femaleadult | Total ground sample count of adult females |
| 9 | Maleadult | Total ground sample count of adult males |
| 10 | Total | Total ground sample count of all age  classes |

**File name: Predicted5_sex.csv**

| **Number** | **Variable name** | **Description** |
| --- | --- | --- |
| 1 | Year | Calendar year |
| 2 | Month2 | Calendar month (1=January,…,12=December) |
| 3 | Month2_fem | Season variable with two levels,namely dry (June-September) and wet (October-May) |
| 4 | dry1 | Total dry season rainfall in mm /220.5998479 lagged by 1 year |
| 5 | max_1 | Average monthly maximum temperature (°C) lagged by 1 month |
| 6 | wet1 | Total wet season (November-June) rainfall in mm/  803.4210315 lagged by 1 year |
| 7 | min_2 | Average monthly minimum temperature (°C) lagged by 2 months |
| 8 | lag0 | Total monthly rainfall in mm/85.66922807 |
| 9 | min | Minimum monthly temperature (°C) |
| 10 | rain7_11 | The running average of the total monthly rainfall in mm lagged by 6 to 10 months/85.66922807  =((lagrain6+lagrain7+lagrain8+lagrain9+lagrain10)/  5)/85.66922807 |

**File name: Mara_newborns_birthrate_interpolation.csv**

| **Number** | **Variable name** | **Description** |
| --- | --- | --- |
| 1 | Year | Calendar year |
| 2 | Month2 | Calendar month(1= January,…,12= December) |
| 3 | Species | Species name |
| 4 | Ratio | Total number of newborns divided by  the corresponding total number of adult females=apparent birth rate |

**File name: Topi_hartebeest_impute3_new.csv**

| **Number** | **Variable name** | **Description** |
| --- | --- | --- |
| 1 | SP | Species name |
| 2 | Source | Indicates whether the observation is a population  estimate from an actual aerial survey (**obs)** or is  predicted (**predict)** by a semiparameteric generalized linear mixed model assuming a negative binomial distribution and a log link function |
| 3 | Year | Calendar year |
| 4 | Month | Calendar month (1= January,…,12=December) |
| 5 | Pred | Estimated or predicted total population size based on the aerial surveys |
| 6 | lower | 95% lower bound of the model estimate |
| 7 | upper | 95% upper bound of the model estimate |

**File name: Fsexratio_parameters_07JUNE2020.xlsx**

| **Number** | **Variable name** | **Description** |
| --- | --- | --- |
| 1 | Effect | Name of the regression components |
| 2 | Month2_fem | Season of the of the monthly effect, namely dry (June to September of the current year) or wet (October of the previous year to May of the current year) |
| 3 | Estimate | GLM estimate of the regression components |
| 4 | StdErr | Standard error of the estimate |
| 5 | Lower | 95% lower bound of the estimate |
| 6 | Upper | 95% upper bound of the estimate |

**File name: Females_parameters_07JUNE2020.xlsx**

| **Number** | **Variable name** | **Description** |
| --- | --- | --- |
| 1 | Effect | Name of the regression components |
| 2 | Month2_fem | Season with two levels, of the of the monthly effect, namely dry (June to September of the current year) or and wet (October of the previous year to May of the current year) |
| 3 | Estimate | GLM estimate of the regression components |
| 4 | StdErr | Standard error of the estimate |
| 5 | Lower | 95% lower bound of the estimate |
| 6 | Upper | 95% upper bound of the estimate |

**File name: Half_parameters_11JUNE2021_new.xlsx**

| **Number** | **Variable name** | **Description** |
| --- | --- | --- |
| 1 | Effect | Name of the regression components |
| 2 | Month2 | Month variable with 12 levels (January to December) |
| 3 | Estimate | GLM estimate of the regression components |
| 4 | StdErr | Standard error of the estimate |
| 5 | Lower | 95% lower bound of the estimate |
| 6 | Upper | 95% upper bound of the estimate |

**File name: newborn_parameters_07JUNE2020.xlsx**

| **Number** | **Variable name** | **Description** |
| --- | --- | --- |
| 1 | Effect | Name of the regression components |
| 2 | Estimate | GLM estimate of the regression components |
| 3 | StdErr | Standard error of the estimate |
| 4 | Lower | 95% lower bound of the estimate |
| 5 | Upper | 95% upper bound of the estimate |

**File name: Quarter_parameters_07JUNE2020.xlsx**

| **Number** | **Variable name** | **Description** |
| --- | --- | --- |
| 1 | Effect | Name of the regression components |
| 2 | Month2 | Month variable with 12 levels (January to December) |
| 3 | Estimate | GLM estimate of the regression components |
| 4 | StdErr | Standard error of the estimate |
| 5 | Lower | 95% lower bound of the estimate |
| 6 | Upper | 95% upper bound of the estimate |
