# Supplementary material for: Hierarchical Bayesian Integrated Modeling of Age- and Sex-Structured Wildlife Population Dynamics
Source: J Agric Biol Environ Stat. 2024 Jul 2;30(4):1068–93. doi: 10.1007/s13253-024-00634-w (PMC12559060; doi:10.1007/s13253-024-00634-w)
Supplement: Supplementary file 5 — (zip 1301 KB) [file 13253_2024_634_MOESM5_ESM.zip › R code_Input_Output_Data_Subroutines_Function_Descriptions/Description_of_R_functions.docx]

**Description of 56 R functions or subroutines used for fitting the integrated state space model**

Assisting_code.R

1. assist_all_densities() <- Calculates the likelihood function $L_{3}$() of the unobserved states, specified in equation 3 in the Supplementary materials.
2. Pois_mean_simple() <- Calculates hyperparameters for generating $\lambda_{t}^{T}$ (see Table 2 of the main text).
3. birth_rate_cal_new3 () <- Calculates birth recruitment rates (r(t)) for a given set of parameters and covariates at time t = 0 (see Table 4 of the main text).
4. terms_initial() <- Calculates the term $h_{0}$(Npop(t)) for calculating and simulating initial states (equations 16-23 in the Supplementary materials).
5. s_a_calc_t() <- Calculates the survival rates for the adult age class for a given parameter set and covariates (see Table 4 of the main text).
6. convert_alphabeta_diff() <- Calculates the terms ($\alpha_{1}$, $\beta_{1}$,…) etc., specified in Table 2 of the main text.
7. sum_years() <- Calculates the sum of the elements of a vector.
8. update_lambda() <- Simulates $\lambda_{1}$, $\lambda_{2}$,… etc. terms specified in Table 2 of the main text.
9. update_lambda_T() <- Simulates the $\lambda_{t}^{T}$ terms specified in Table 2 of the main text.
10. update_nba_terms() <- Calculates the term $f_{0}$ specified on page 25 of the Supplementary materials.
11. birth_rate_cal_t_new3() <- Calculates birth recruitment rates (r(t)) for a given total population size for t = 1, 2, …, 174 (see Table 4 of the main text).
12. assist_other_densities1() <- Calculates likelihood terms (a part of equation 3 in the supplementary materials) involving quarters and half-yearlings only.
13. NA19_densities1_test() <- Calculates the likelihood term (a part of equation 3 in the supplementary materials) for the animals which have survived the half-yearling stage and graduated to adulthood.

**state_int_update_simple_diff.R**

1. birth_rate_cal_m_newbirth3() <- Subroutine which helps to calculate birth recruitment rates and is used to calculate acceptance probabilities for P(t, k) (see Table 2 in the main text) at the Metropolis-Hastings step.
2. s_a_calc_m() <- Subroutine which helps to calculate survival rates of adults and is used to calculate acceptance probabilities for P(t, k) (see Table 2 in the main text) at the Metropolis-Hastings step.
3. update_NBAfless8_lagged_int() <- Subroutine which helps to simulate P(t, k), 1<= k <= 10, k $\neq$ 3 and k $\neq$ 4; 1<= t <= 173 (see Table 2 in the main text).
4. update_NBAf3_lagged_int() <- Subroutine which helps to simulate P(t, k), k = 3; 1<= t <= 173 (see Table 2 in the main text).
5. update_NBAf8_lagged_int() <- Subroutine which helps to simulate P(t, k), k = 11; 1<= t <= 173 (see Table 2 in the main text).
6. update_NBAf9_lagged_int() <- Subroutine which helps to simulate P(t, k), k = 12; 1<= t <= 173 (see Table 2 in the main text).
7. update_NAf_lagged_int() <- Subroutine which helps to simulate $F_{a}$(t), 1<= t <= 173 (see Table 2 in the main text).
8. update_NAm_lagged_int() <- Subroutine which helps to simulate $M_{a}$(t), 1<= t <= 173 (see Table 2 in the main text).
9. update_SAm_lagged_int() <- Subroutine which helps to simulate M(t), 1<= t <= 173 (see Table 2 in the main text).
10. update_Yearlkft_lagged_int() <- Subroutine which helps to simulate G(t, k), 7 <= k <= 18; 1<= t <= 173 (see Table 2 in the main text).
11. update_Yearl18ft_bound_int() <- Subroutine which helps to simulate G(t, k), k = 19; 1<= t <= 173 (see Table 2 in the main text).
12. update_Q_2ft_lessT_lagged_int() <- Subroutine which helps to simulate G(t, k), k = 1; 1<= t <= 173 (see Table 2 in the main text).
13. update_Q_3ft_lagged_int() <- Subroutine which helps to simulate G(t, k), 2 <= k <=5; 1<= t <= 173 (see Table 2 in the main text).
14. update_Q_10ft_lagged_int() <- Subroutine which helps to simulate G(t, k), k = 6; 1<= t <= 173 (see Table 2 in the main text).
15. update_Nnew_lessT_int() <- Subroutine which helps simulate N(t), 1<= t <= 173 (see Table 2 in the main text).
16. update_NBAf4_lagged_int() <- Subroutine which helps to simulate P(t, k), k = 4; 1<= t <= 173 (see Table 2 in the main text).
17. T_count_update() <- Subroutine which helps to simulate $\Psi_{t}$, k = 4; 1<= t <= 173 (see Table 2 in the main text).

**state_terminal_integrated_diff.R**

1. update_NBAfless8_bound_int() <- Subroutine which helps to simulate P(t, k), 1<= k <= 10, k $\neq$ 3, 4; t = 174 (see Table 2 in the main text).
2. update_NBAf3_bound() <- Subroutine which helps to simulate P(t, k), k = 3; t = 174 (see Table 2 in the main text).
3. update_NBAf4_bound() <- Subroutine which helps to simulate P(t, k), k = 4; t = 174 (see Table 2 in the main text).
4. update_NBAf8_T_bound() <- Subroutine which helps to simulate P(t, k), k = 11; t = 174 (see Table 2 in the main text).
5. update_NBAf9_T_bound() <- Subroutine which helps to simulate P(t, k), k = 12; t = 174 (see Table 2 in the main text).
6. update_NAf_bound_new() <- Subroutine which helps to simulate $F_{a}$(t), t = 174 (see Table 2 in the main text).
7. update_NAm_bound_new() <- Subroutine which helps to simulate $M_{a}$(t), t = 174 (see Table 2 in the main text).
8. update_SAm_bound_new() <- Subroutine which helps to simulate M(t), t = 174 (see Table 2 in the main text).
9. update_Yearlkft_bound() <- Subroutine which helps to simulate G(t, k), 7 <= k <= 19; t = 174 (see Table 2 in the main text).
10. update_Q_2ft_T_bound() <- Subroutine which helps to simulate G(t, k), k = 1; t =174 (see Table 2 in the main text).
11. update_Q_3ft_bound() <- Subroutine which helps to simulate G(t, k), 2 <= k <=6; t = 174 (see Table 2 in the main text).
12. update_NnewT() <- Subroutine which helps to simulate N(t), t = 174 (see Table 2 in the main text).

**State_initial_int.R**

1. initial_updateNAf01() <- Subroutine which helps to simulate P(t, k), k = 1, t = 0 (see Table 2 in the main text).
2. initial_updateNAf0_lesspreg_new1() <- Subroutine which helps to simulate P(t, k), 2 <= k <= 12; t = 0 (see Table 2 in the main text).
3. initial_updateMt_new1() <- Subroutine which helps to simulate M(t), k = 4; t = 0 (see Table 2 in the main text).
4. initial_update() <- Subroutine which helps to simulate N(t), t = 0 (see Table 2 in the main text).
5. initial_updateQ() <- Subroutine which helps to simulate P(t, k), 2 <= k <= 6; t = 0 (see Table 2 in the main text).
6. initial_hy_calc() <- Subroutine which helps to generate starting values of P(t, k) for MCMC simulation, 7<= k <= 19, 1<= t <= 174 (see Table 2 in the main text).
7. initial_updateY18() <- Subroutine which helps to simulate $P$(t, k), 7<= k <= 19, t = 0 (see Table 2 in the main text).

**sigma_modified.R**

1. update_sigobs_modified_diff() <- Subroutine which helps to simulate $\sigma_{1}$, $\sigma_{2}$ (see Table 2 in the main text) etc.
2. update_sigmaT_modified() <- Subroutine which helps to simulate $\sigma_{T}$ (see Table 2 of the main text).

**survival_rates_sex_ratio_simple.R**

1. update_R_r_int() <- Subroutine for simulating alpha, needed to calculate r(t), 1<= t <= 174 (see Table 4 in the main text).
2. update_s_a_int() <- Subroutine for simulating alpha, needed to calculate of $s_{a}$(t), 1<= t <= 174 (see Table 4 in the main text).
3. update_s_y_int_new() <- Subroutine for simulating alpha, needed to calculate $s_{h}$(t), 1<= t <= 174 (see Table 4 in the main text).
4. update_s_q_int() <- Subroutine for simulating alpha, needed to calculate of $s_{q}$(t), 1<= t <= 174 (see Table 4 in the main text).
5. update_sexratio() <- Subroutine for simulating alpha, needed to calculate sex ratio $\phi$(t), 1<= t <= 174 (see Table 4 in the main text).
