# Supplementary material for: Hierarchical Bayesian Integrated Modeling of Age- and Sex-Structured Wildlife Population Dynamics
Source: J Agric Biol Environ Stat. 2024 Jul 2;30(4):1068–93. doi: 10.1007/s13253-024-00634-w (PMC12559060; doi:10.1007/s13253-024-00634-w)
Supplement: Supplementary file 7 — (pdf 211540 KB) [file 13253_2024_634_MOESM7_ESM.pdf]

Supplementary materials for the paper "Hierarchical Bayesian state-space modeling of age-and sex-structured wildlife population dynamics"

Sabyasachi Mukhopadhyay[1,2], Hans-Peter Piepho[1], Sourabh Bhattacharya[3], Holly T. Dublin[4], Joseph O. Ogutu[1]

[1]University of Hohenheim, Institute of Crop Science, Biostatistics Unit (340C), Fruwirthstrasse 23,
70599 Stuttgart Germany
[2]Indian Institute of Management, Udaipur, India
[3]ISRU, Indian Statistical Institute,
203, B.T. Road, Kolkata-108, India
[4]IUCN ESARO, Wasaa Conservation Centre,
P.O. Box 68200, Nairobi, Kenya, 00200
Email for correspondence: `jogutu2007@gmail.com, sabymstat@gmail.com`

S.1 Data Details

Two main data types are used to model topi population dynamics: animal counts and meteorological data. The animal count data consists of age-and sex-structured ground vehicle sample counts and aerial sample counts. The ground vehicle sample counts are not processed except that the count totals from the three transects are tallied to produce a count total for each survey month for the different age and sex classes. However, the aerial sample counts are used to obtain estimates of the total topi population size. These estimates are calculated for the Mara Ecosystem and each of its four zones: Mara Reserve,

Mara Conservancies, Loita Plains and Siana (encompassing Siana Plains, Naikara and Loita), for each of the 75 surveys, using Jolly's Method 2 for unequal length transects (Jolly, 1969) as explained in more detail in Section S.1.3. The unprocessed aerial sample count data are used in the balanced bootstrap procedure described in Section 5.2 in the main text and in Section S.3.2 below.

The rainfall records were taken from S1Data.csv in the Supplementray materials of Bartzke et al. (2018), available at https://doi.org/10.1371/journal.pone.0202814.s001.xlsx.

S.1.1 Data sets for the ground sample count data, aerial sample count data and aerial sample count data used in the balanced bootstrap

The ground counts of animals classified according to age and sex are provided in the data set `TopiData.csv`. The descriptions of the column headings in the `TopiData.csv` are given in Table S1.

Table S1: Names and descriptions of the variables in the data set `TopiData.csv`

Column	Variable name	Description
1	Species	Name of the species
2	Year	Year in which ground sample count was done, with values $1989, 1990, \ldots, 2003$
3	Month	Month in which ground sample count was done, with values $1, 2, \ldots, 12$
4	New	Number of newborn topi recorded
5	Quarter	Number of topi in quarter-size age class
6	Hyearling	Number of topi in half-yearling age class
7	Femaleadult	Number of adult female topi
8	Maleadult	Number of adult male topi

The total topi population size estimated from the aerial sample survey data are provided in the data set `AerialData.csv`. The descriptions of the column headings in this data set are given in Table S2.

Table S2: The names and descriptions of variables contained in the data set `Aerial-Data.csv`.

Column	Variable name	Description
1	Species	Name of the species
3	Year	Year in which aerial sample survey was done, with values $1989, 1990, \ldots, 2003$
4	Month	Month in which aerial sample survey was done, with values $1, 2, \ldots, 12$
5	Population size	Total number of animals estimated from aerial survey

The aerial sample count data used by the balanced bootstrap method is provided in the data set `Topi_bootstrap.csv`. The descriptions of the column headings in this data set are given in Table S3. Jolly's (1969) Method 2 is used to estimate the total population size from the bootstrap samples for each survey.

Table S 3: The names and descriptions of variables contained in the data set `Data_file_bootstrap.csv`. Details of the procedure for generating the estimated population sizes are given in Sections 5.1 of the main text and Section S.3.2.

Column	Variable name	Description
1	Replicate	The bootstrap replicate used for prediction
3	Year	Year in which aerial sample survey was done, with values $1989, 1990, \ldots, 2003$
4	Month	Month in which aerial sample survey was done, with values $1, 2, \ldots, 12$
5	Population	Total number of animals estimated for the balanced bootstrap sample
6	New	Number of simulated newborn topi
7	Quarter	Number of simulated quarter-size topi
8	Hyearling	Number of simulated half-yearling topi
9	Femaleadult	Number of simulated adult female topi
10	Maleadult	Number of simulated adult male topi

All the data sets are included in the folder named 'Data_Dictionary_new' in the supplementary materials. This folder also contains a complete dictionary of all the variables,

model parameters, initial state and parameter values and transformations of each variable used in the topi population dynamics model in a document called Data_Dictionary.docx.

S.1.2 Additional details on ground vehicle age and sex composition sample surveys

The Masai Mara Ecological Monitoring Program (MMEMP) carried out monthly ground vehicle sample counts of seven ungulate species (and of ostrich *Struthio camelus massaicus*), including topi, for 174 months from July 1989 to December 2003, in the 1530 km^2 Masai Mara National Reserve (1°13′–1°45′S, 34°45′–35°25′E) and in its adjoining pastoral lands to the north and northeast. This region (Mara) is located in the Narok County of Kenya and is the northern-most section of the Greater Mara–Serengeti ecosystem, covering about 40,000 km^2 in Kenya and Tanzania. The counts were conducted in three counting blocks, each having a fixed strip transect (Figure S1). The transects jointly sampled 29.4% (450.4 km^2) of the Reserve (Ogutu et al. 2008*a*, 2009). Counts were not carried out on one, two or all the three transects in 17 months because of heavy rains, logistical, budgetary, and other constraints. The 17 months were distributed over nine years and eight calendar months (Ogutu et al., 2014*b*). To determine the functional forms of the relationships between birth rate, survival rates, or adult sex ratio and the covariates, all the missing ground counts were imputed using a transect-specific state-space model, separately for each age and sex class (Piepho and Ogutu, 2007; Ogutu et al., 2014*b*). The full model was fit by the MCMC method using the original ground count data, which included all the missing values.

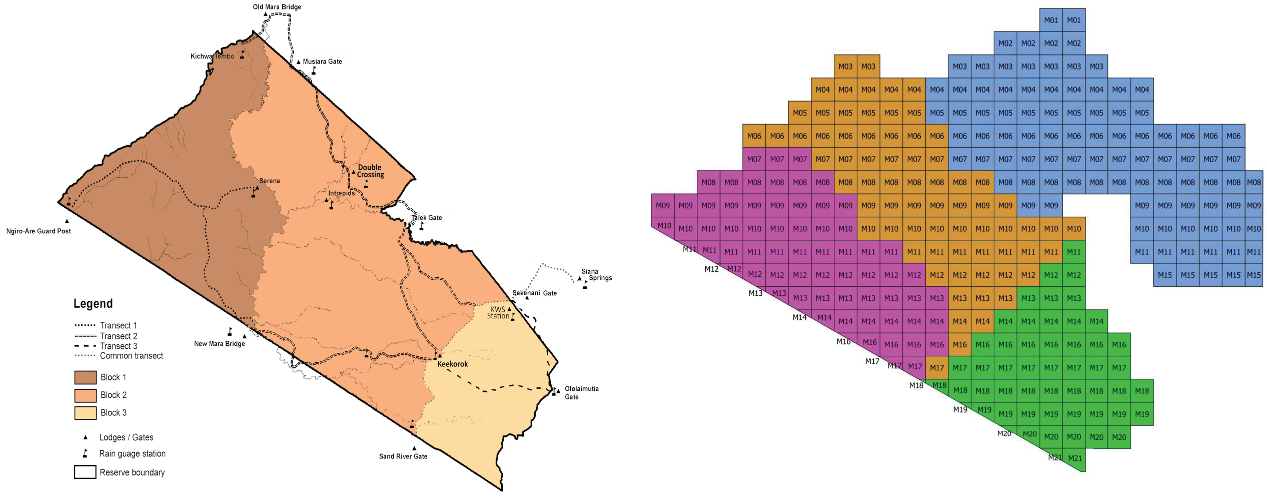

(a) (b)

Figure S1: a) Map of the Masai Mara National Reserve showing the three counting blocks and the three fixed road transects used for the ground vehicle age- and sex-composition sample counts from July 1989 to December 2003, b) map of the Masai Mara Ecosystem showing its four constituent zones comprising the Masai Mara National Reserve (magenta, 1561.1 km^2), Mara Conservancies (dark orange, 1775.0 km^2), Loita Plains (cornflower blue, 2372.1 km^2) and Siana (green, 1224.8 km^2). The alphanumeric labels identify sampling units used in the aerial surveys. A row of sampling units form East-West or West-East transects whereas a column of sampling units form a South-North or North-South transects. The sampling units and transects have remained fixed since monitoring began in 1977.

The complete groups were recorded by date, counting block, species and number of individuals in each age and sex class during each count. A combination of body size, coat colour, horn length and shape was used to assign animals to size classes comprising newborn, quarter, half-yearling, three-quarter and adult size (see Figure S3 for topi). Animals were highly visible because of open grasslands, minimising the likelihood of misclassification into age–sex classes. Chances of misclassification of fully grown topi were further reduced by amalgamating the adult and three-quarter size classes (Ogutu et al. 2008*b*,

2014*a*).

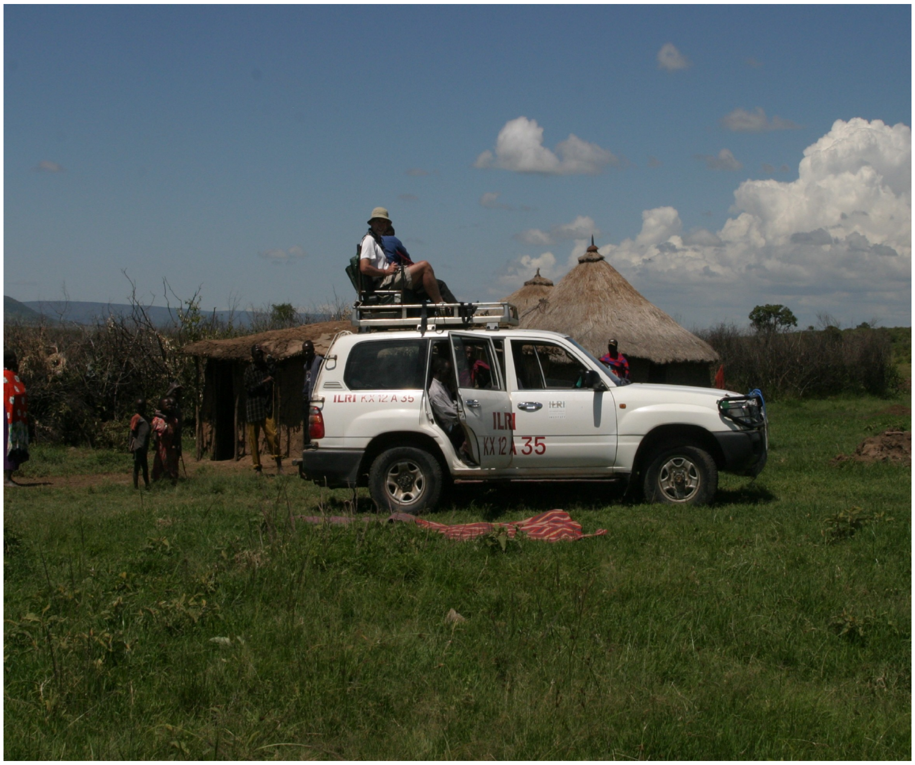

Figure S2: A vehicle used for age and sex composition ground sample counts in Masai Mara in 2005 and 2006. During July 1989 to December 2003, counting, aging and sexing of animals during the ground counts was done by one observer sitting inside the vehicle. But in 2005 and 2006, the observer and recorder sat on seats attached to the roof rack of the counting vehicle. Note that only the data for July 1989 to December 2003 are used in this paper.

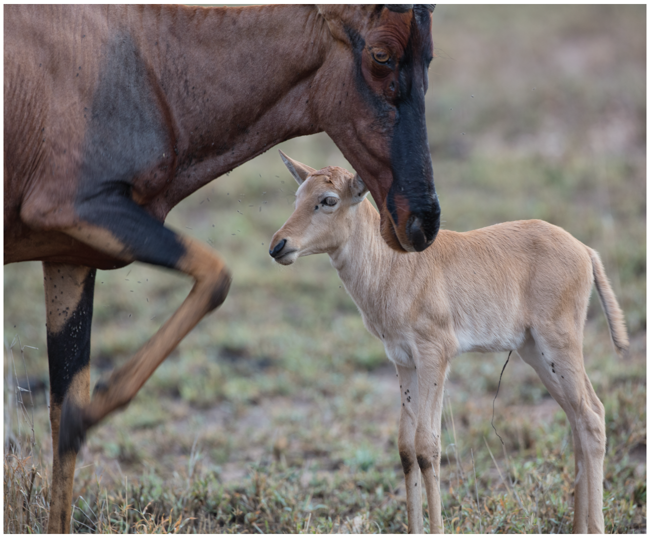

(a)

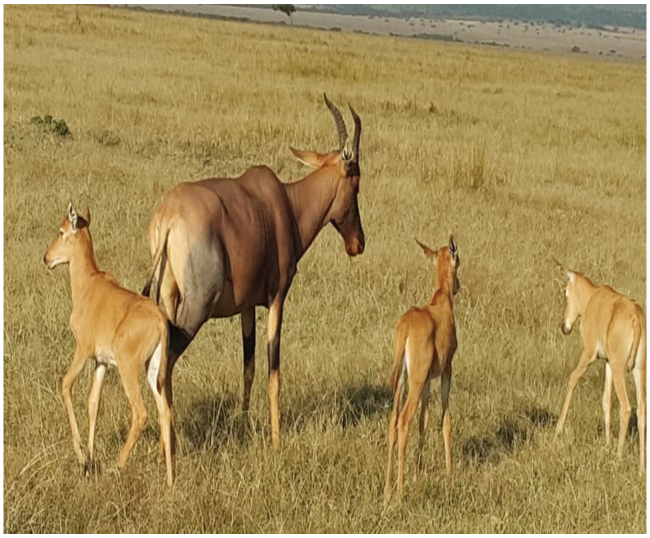

(b)

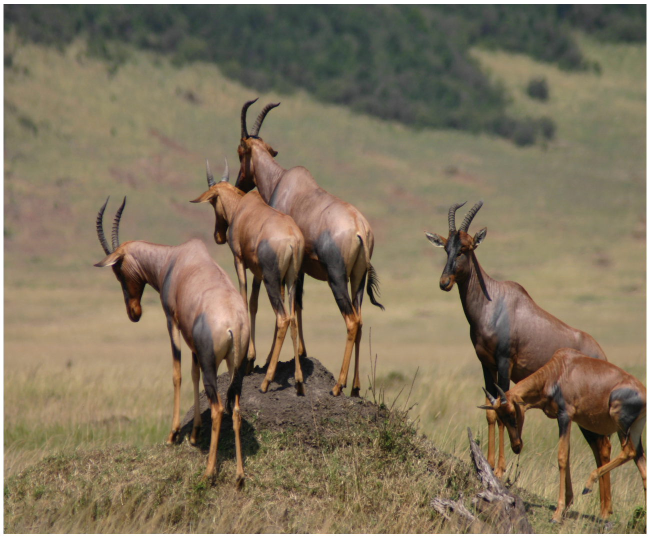

(c)

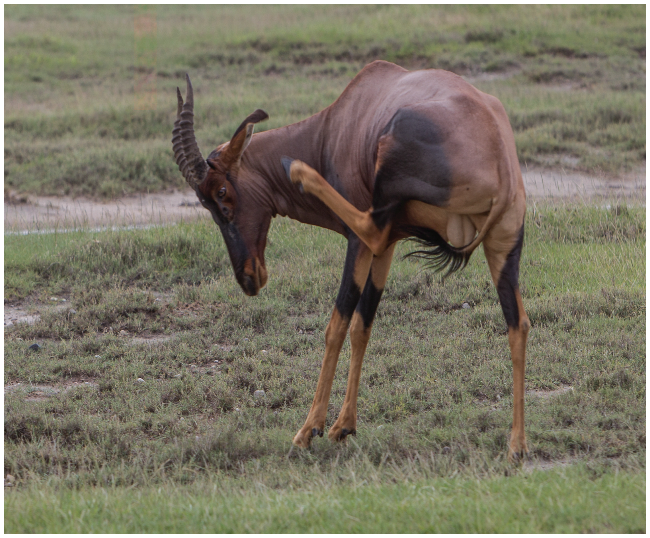

(d)

Figure S3: Pictures showing a) adult female topi with a newborn calf with the umbilical cord still attached, b) adult female topi with three quater-size calves, c) three adult topi with two half-yearlings and d) an adult male topi in Masai Mara.

The monthly ground vehicle counts were carried out along the three strip-transects out to 1000 m either side of the transect centre line. The vehicle (Figure S2) was driven off the transect path to within 200 m of each sighted group and stopped to reliably assign individuals to age and sex classes and count animals using binoculars. All sighted herds of the target species were counted. The same transects and counting procedures were used throughout the monitoring period to ensure representative and consistent coverage. Year round accessibility by car dictated the choice of transect paths. Each count took 4 days to conduct, on average, with a counting day starting at 7–7:30 hrs and ending at 14–15 hrs.

For constructing the model, the apparent monthly topi birth rate (or recruitment) was estimated as the total number of newborns, divided by the corresponding number of adult females counted in each month. The month of conception was estimated by backdating recorded birth months by the topi gestation length (Western, 1979). The complete ground sample count and total monthly rainfall data for 15 stations are available in Ogutu et al. (2015).

S.1.3 Additional details on aerial sample surveys

The Directorate of Resource Surveys and Remote Sensing (DRSRS) flights are carried out using high-winged aircraft (Cessna 186 or Partenavia 68) equipped with Global Navigation Systems, a Global Positioning System, Internal Communication System and radar altimeters for accurate navigation and mapping of animal distribution (Figure S4a). The survey flights follow systematic, east-west oriented and parallel flight lines or transects spaced typically 5 km apart, following the Universal Transverse Mercator (UTM) coordinate system. North-south transects are used only where the terrain makes east-west flying at low altitude too dangerous to undertake. Each transect is subdivided into equal, 5 km long sample intervals (Ogutu et al., 2016). A typical sampling unit is thus 5 km $\times$ 5 km (n = 46 surveys) but 27 surveys had a higher (5 km $\times$ 2.5 km) whereas the first two surveys in 1977 had a lower (5 km $\times$ 10 km) spatial resolution. The total number of sampling units varied slightly across surveys during 1977–2018 (261.2 $\pm$ 60.99, range 134–498 units, n = 75 surveys).

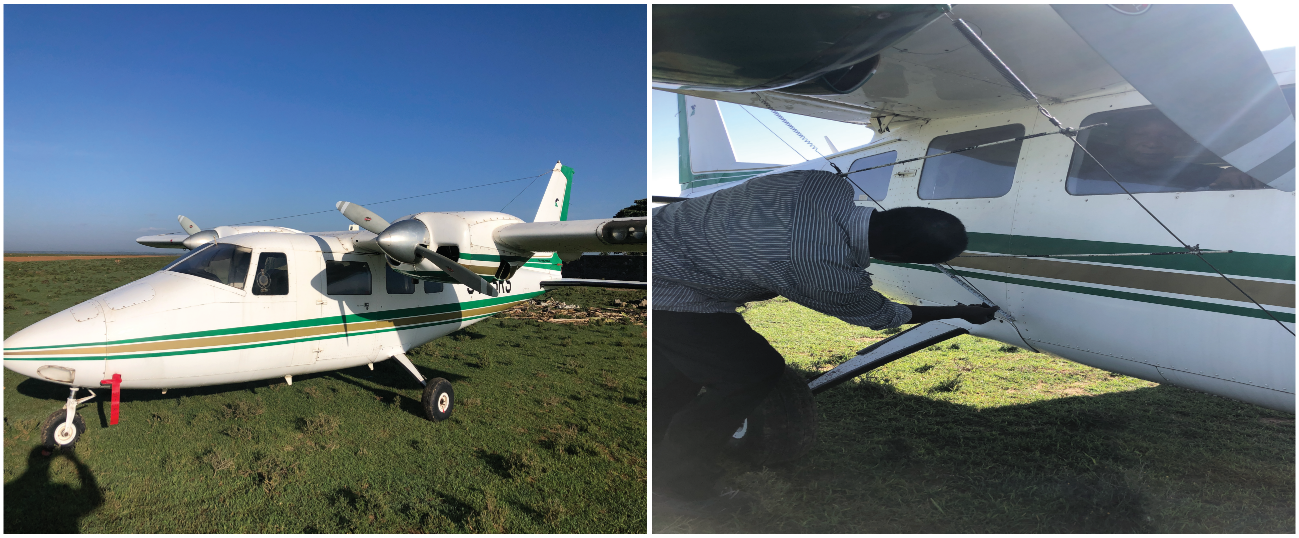

(a) (b)

Figure S4: Pictures of a) a twin engine, high wing, turbocharged Partenavia P.68 aircraft used in the DRSRS aerial surveys, b) head of aerial surveys at DRSRS, Mr. Gordon Ojwang calibrating the counting strip width by fixing metal rods (streamers) to the wing struts of the aerial survey aircraft.

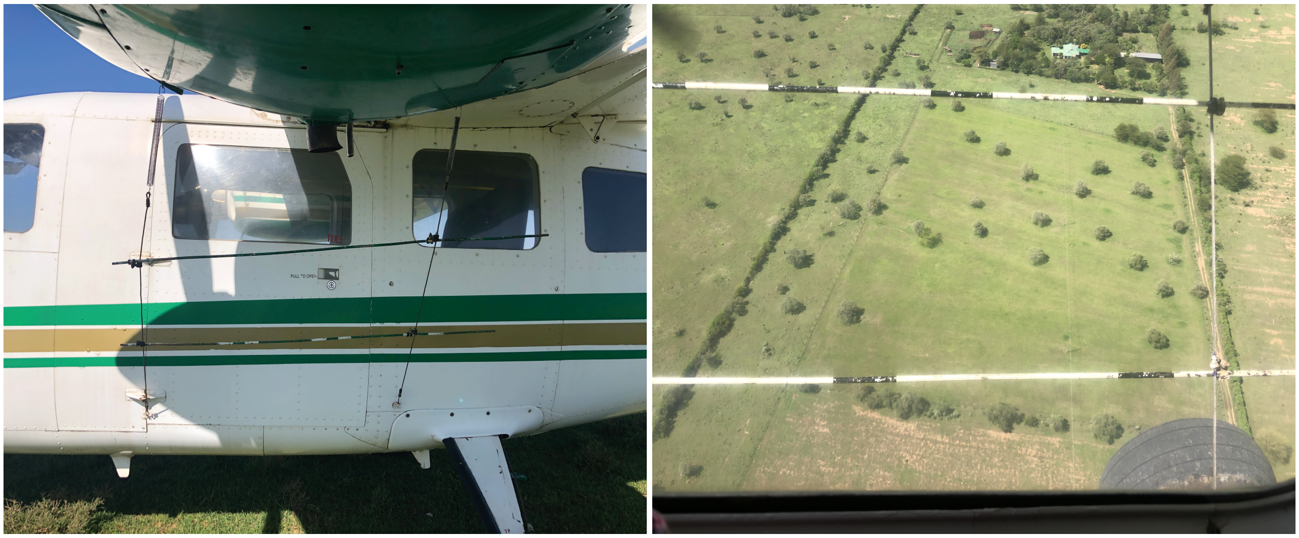

(a) (b)

Figure S5: Pictures of a) metal rods attached to the aircraft's wing struts for marking counting strips (141 m) on the ground on either side of the aircraft's flight line and b) a counting strip (141 m) on one side of the aircraft during a survey flight. Only animals seen within this strip on either side of the aircraft are counted.

Prior to each survey the aircraft is calibrated to the defined survey strip width (Figures S4b and S5a). Animals are counted only within observation strips defined on the ground by two parallel rods (streamers) attached to the wing struts of the aircraft (Figure S5b). The crew consists of a pilot and one front-seat observer who records land use and cover and other environmental variables (Figure S6), and two rear-seat observers who count and record animals and settlements sighted within the strip width on either side of the aircraft (Figure S7). The average strip width was 266.1 $\pm$ 29.78 m (range 210–366 m, n = 75 surveys) and the average flying height above ground level was 75–122 m. The flight speed varies with terrain features but never falls below 190 km /hour. The average coverage or sampling intensity was 5.25 $\pm$ 1.61% (range 2.0–10.88%, n = 75 surveys).

Observations were recorded on video tapes. A 35 mm analogue camera was used to take oblique photos of groups of more than 10 animals within the strip width for later

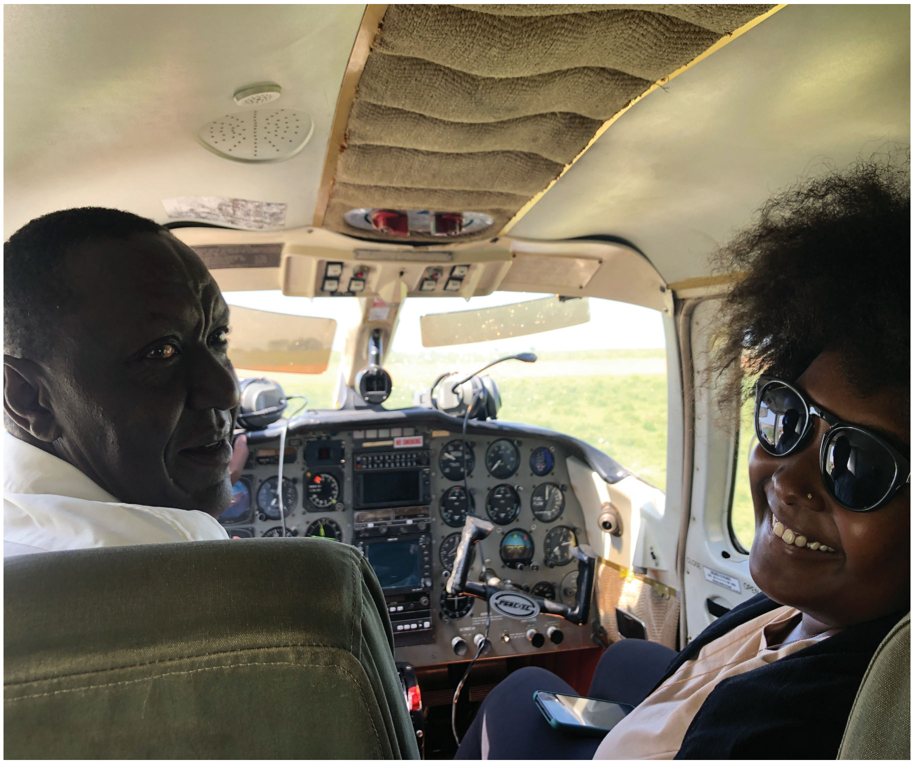

Figure S6: Photo showing the pilot, Captain Goodwin Wachira, and the front seat observer (FSO), Cindy Obath, during a DRSRS aerial survey in the Masai Mara Ecosystem in March 2022.

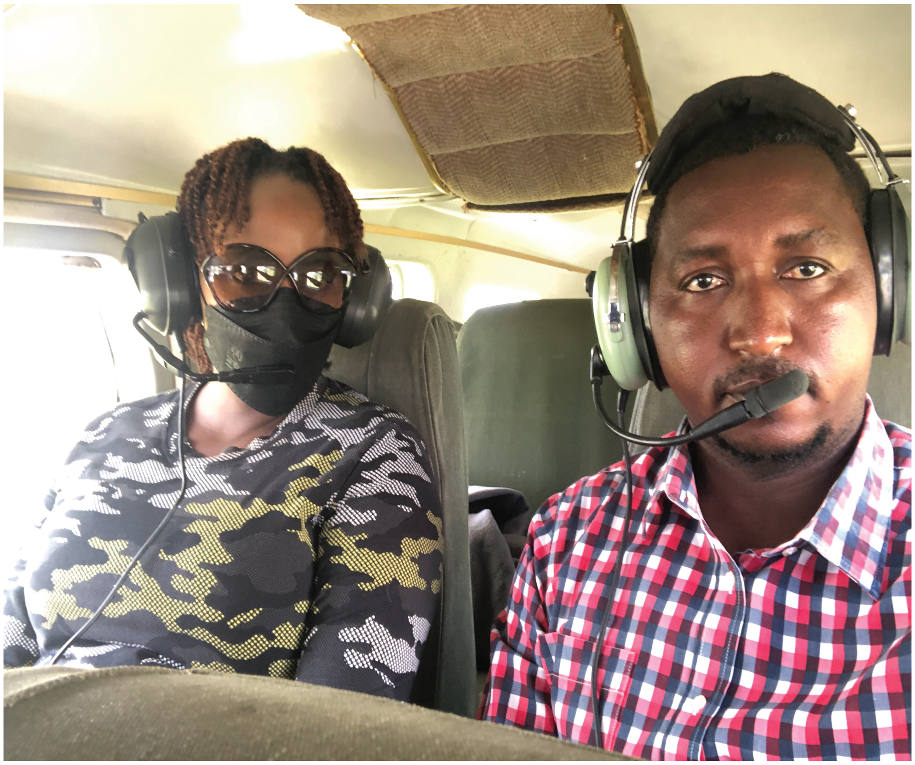

Figure S7: Photo showing the right rear seat observer, Jully S. Senteu, and the left rear seat observer, Stephen M. Ndambuki, during a DRSRS aerial survey in the Masai Mara Ecosystem in March 2022.

correction of the visual estimates from 1977 to 1990s. The photos were captured on film rolls, which were then projected onto a large screen for accurate counting. From 1990s onwards, 35 mm digital cameras were used. The digital photos (Figure S8 and S9) are downloaded and animals counted on large computer or digital television screens. Animals in earlier photos were counted with the aid of binocular microscopes or data projectors. Jolly's Method 2 (Jolly, 1969) for transects of unequal lengths is used to estimate population size and its standard error. The accuracy of population estimates derived from the aerial sample surveys ranges between 71% and 83% or higher (Ottichilo and Khaemba, 2001; Georgiadis, 2011).

A semiparametric generalized linear mixed regression model assuming a negative binomial error distribution and a log link function was used to interpolate the 75 aerial surveys to cover all the 174 months spanned by the ground vehicle surveys (Ogutu et al., 2014*b*; Ogutu et al., 2016). Only 14 aerial surveys were done in the period covered by the ground counts spanning July 1989 to December 2003.

The imputed total topi population size for the period July 1989 to December 2003 are not used in the MCMC model fitting. They are merely used at the screening stage to calculate possibly lagged topi population size used as a covariate representing density dependence in the logistic regression models used to obtain estimates of the initial regression parameter estimates for topi survival and recruitment rates.

S.1.4 Additional details on the Mara-Serengeti and other topi populations

Topi weigh 91-147 kg, have elongate and narrow faces, ringed and lyrate horns. In captivity, the life-span of topi that live to three years is 8-9 years with no significant difference between males and females but some animals can live to 23-24 years of age (Duncan, 2014). They are widely but patchily distributed across Africa from South Africa and Botswana in the south where they are called tsessebe, to South Sudan in the north and Nigeria in the west, where they are called tiang. The six subspecies or geographical races of *Damaliscus lunatus* form two distinct groups. The first group comprises western races found in arid desert areas such as *D. l. korrigum*. In Kenya, the topi primarily inhabits highland grasslands, areas with a mix of trees and grassland, coastal forests, and bush-

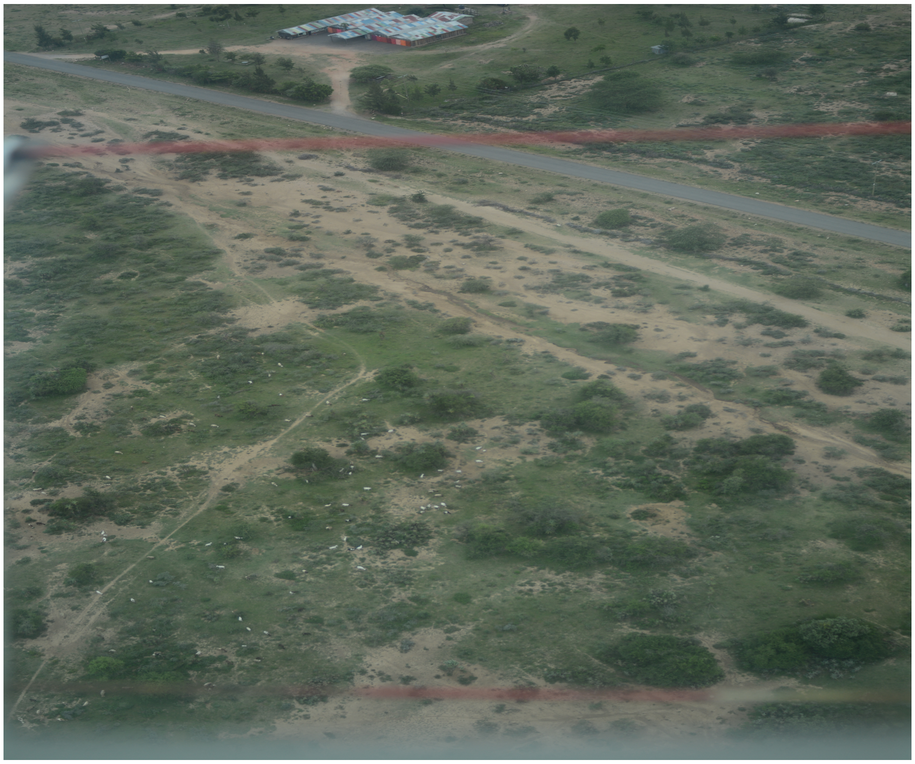

Figure S8: Example of digital oblique aerial photos of a flock of sheep and goats (shoats) taken at 400 feet above ground by a rear seat observer during a DRSRS survey in the Masai Mara Ecosystem. The photos are magnified on a large digital screen before counting the animals.

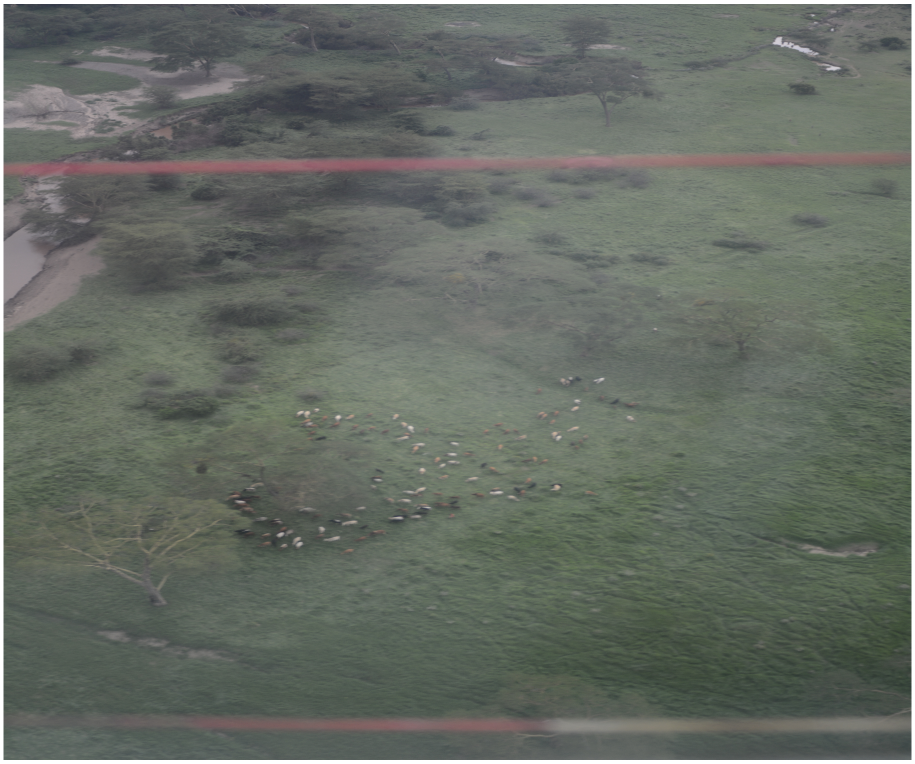

Figure S9: Example digital oblique aerial photos of a herd of cattle taken at 400 feet above ground by a rear seat observer during a DRSRS survey in the Masai Mara Ecosystem. The photos are magnified on a large digital screen before counting the animals.

lands. Exceptionally, it can also be found in desert-scrub environments. Its habitat ranges from sea level to elevations of approximately 2134 m. Early in the 20-th century, topi were abundant in certain areas of Kenya. They were notably plentiful near the Turkwell River by Lake Turkana in northwestern Kenya and in the southern coastal regions near Mombasa. The animals located to the northwest of Lake Turkana were identified as tiang (*Damaliscus korrigum tiang*) whereas the remaining population in the region was classified as the topi (*Damaliscus korrigum korrigum*) (Stewart and Stewart, 1963). The topi also had a broader presence in the northern part of Kenya by 1909. Notably, topi were sighted as far as Mt. Marsabit in 1911. However, the full extent of their range during this early time is unclear. Since then, the topi population in Kenya has significantly declined. In two initial locations (near Lake Turkana and south of Mombasa), topi no longer exist. In the northern regions, their presence has become highly restricted. By 1963 topi had nearly vanished from western Kenya's settled areas largely due to control measures implemented by farmers and increased hunting for food during wartime (WWII) (Percival 1924, 1928; Stewart and Stewart 1963). These subspecies are adapted to migrate substantial distances in search of water during dry seasons. In contrast, the second group includes eastern races such as *D. l. jimela*. These races inhabit fertile, well-watered regions and, as a result, do not engage in long migrations (Sidney, 1965, p. 175-187). They select mesic, perennial grassland or savanna. The Greater Mara-Serengeti Ecosystem supports one of the largest remaining topi populations in East Africa (Jarman and Sinclair, 1979).

Topi are physiologically adapted to environments with high-quality diets, leading to their discontinuous distribution in Kenya and Tanzania (Duncan, 1975, p. 208). They are mostly found within the 750 mm isohyet, except near lakes like Lake Turkana, where grass growth is sustained by water (Sydney, 1965; Duncan, 1975, p. 209). Commonly, topi inhabit high rainfall, swampy areas such as the western Serengeti and Kenyan Lamu (Duncan, 1975, p. 209), and southern Tanzania (Vesey-Fitz-Gerald, 1955, 1960). Similarly, the tiang in Southern Sudan relies on swamps (Duncan, 1975, p. 209). Due to their need for a high-quality diet, topi distribution is limited but they thrive in high rainfall zones, achieving high densities. Their rapid growth and early maturity enable swift recovery from unfavourable climatic periods (Duncan, 1975, p. 209).

Topi likely compete for limiting high-quality food in the dry season and the intensity of this competition likely increases with population density (Jarman and Sinclair, 1979). We

therefore use prior topi population size to index intraspecific competition for resources. Lastly, topi predation is likely higher for the younger age classes and in the wet season. This is because numerous wildebeest (*Connochaetes taurinus*), zebra (*Equus quagga*) and Thomson's gazelle (*Eudorcas thomsoni*) and eland (*Taurotragus oryx*) migrating to, and occupying the Mara in the dry season, from the adjoining Serengeti Ecosystem in Tanzania to the south (southern migration) and Loita Plains in Kenya to the north-east (northern migration, which is virtually extinct since 2020) absorb most of the lion (*Panthera leo*) and spotted hyena (*Crocuta crocuta*) predation pressure falling on the resident Mara ungulates, such as topi (Scheel and Packer, 1979).

Concurrent with their dramatic population decline, topi range has contracted dramatically over time in Kenya. This has led to extirpation of topi populations in western Kenya by overhunting, expanding settlements, agricultural and other land use developments. Where they still occur in Kenya, topi numbers (by between 88% and 99%) and range have drastically reduced in eastern, southwestern, northern and north eastern parts and along the east coast of Kenya (Sidney, 1965; Ogutu et al., 2016; Figures S10 a-d).

Topi numbers declined persistently by 73% (from 35,898 to 9,686 animals) in Kenya's Narok County and by 80% (from 20,204 to 4,031 animals) within the Maasai Mara National Reserve between 1977 and 2016 (Ogutu et al., 2016; Veldhuis et al., 2019). Topi numbers have continued to decline persistently in Narok County of Kenya in the Mara Ecosystem and in Trans Mara (2,049.6 km^2) (Figures S11 a and b). Topi hardly occur in northern Narok (5,083.0 km^2) where only a herd of 6 topi was sighted in December 1985 and another herd of 2 animals in May 1986 since DRSRS surveys began in 1977. Similarly, topi hardly occur in the more arid northeastern Narok County (3,171.4 km^2). Notably, topi numbers continue to decline strikingly in the Masai Mara Ecosystem (Figure S11 a) and each of its four zones regardless of their degree of protection (S12). Thus, between 1977–78 and 2022, topi numbers declined in the Mara Ecosystem by 84.5% (from 40414 in 1977-78 to 6284 in 2022), in the protected Mara Reserve by 77.2% (from 17,877 in 1977–78 to 4,079 in 2022), in the semi-protected Conservancies by 85.5% (from 17,239 to 2,506) and in the unprotected Loita Plains by 93.1% (from 960 to 66) and in the Siana Plains by 99.1% (from 45 to 1) (Figures S11 and S12). The causes of the troubling declines and their relative importance have not yet been fully quantified.

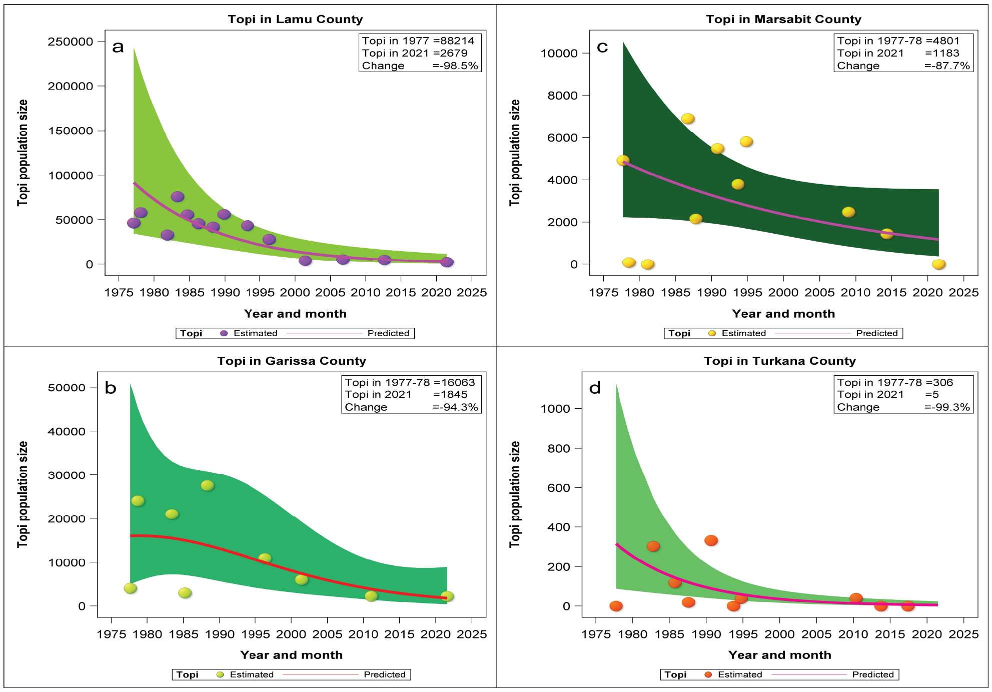

Figure S10: Trend in total topi population size in Lamu, Garissa, Marsabit and Turkana Counties of Kenya estimated (solid circles) from aerial monitoring surveys conducted by the Directorate of Resource Surveys and Remote Sensing (DRSRS) (1977 to 2021) and one aerial total count conducted by the Kenya Wildlife Service (KWS) and Wildlife Research and Training Institute (WRTI) in 2021. The solid line is the fitted trend line whereas the shaded band is the 95% pointwise confidence band.

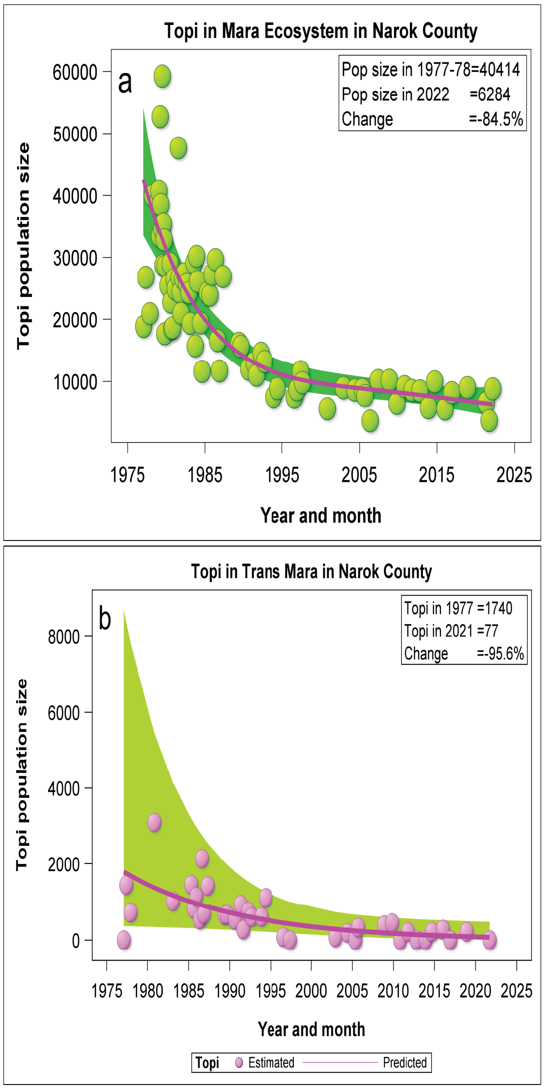

Figure S11: Trend in total topi population size in the Masai Mara Ecosystem and Trans Mara in Narok County estimated (solid circles) from 77 aerial monitoring surveys conducted by the Directorate of Resource Surveys and Remote Sensing (DRSRS) (1977 to 2021) and one aerial total count conducted by the Kenya Wildlife Service (KWS) and Wildlife Research and Training Institute (WRTI) in 2021. The solid line is the fitted trend line whereas the shaded band is the 95% pointwise confidence band.

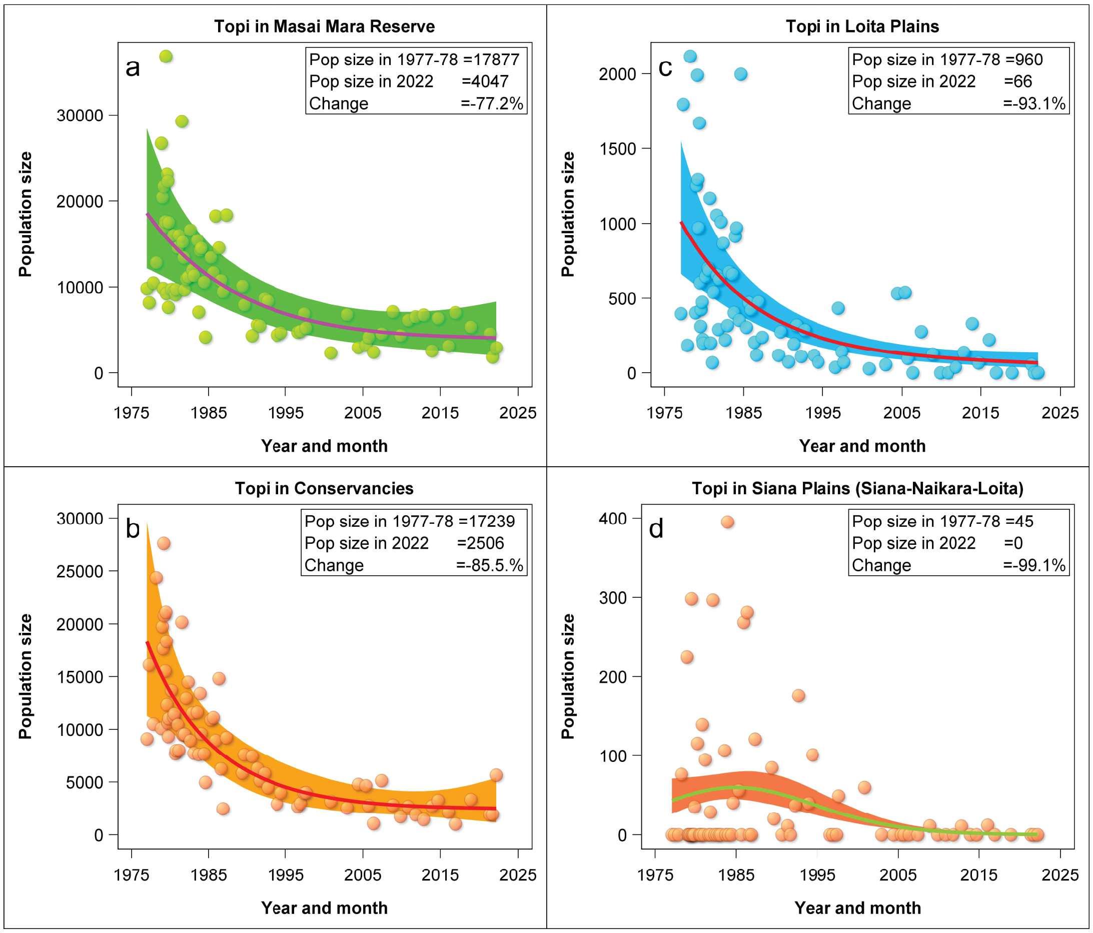

Figure S12: Trend in total topi population size in the a) Masai Mara National Reserve, b) Mara Conservancies, c) Loita Plains and d) Siana-Naikara-Loita (Siana Plains or simply Siana) estimated (solid circles) from 77 aerial monitoring surveys conducted by the Directorate of Resource Surveys and Remote Sensing (DRSRS) (1977 to 2022) and one aerial total count conducted by the Kenya Wildlife Service (KWS) and Wildlife Research and Training Institute (WRTI) in 2021. The solid line is the fitted trend line whereas the shaded band is the 95% pointwise confidence band.

S.1.5 Additional details on rainfall and temperature

In the Mara, rain falls in the wet (November–June) and dry (July–October) seasons. The wet season consists of the early wet-season (November–February, "short rains") and the late wet-season (March–June, "long rains") components. Rainfall distribution is thus bimodal, with a secondary peak in December–January separated from a primary peak in April–May by a short dry season in February (Ogutu et al., 2008*a*). Severe El Niño Southern Oscillation (ENSO) droughts in 1993, 1997 and 1999–2000, plus mild droughts in 1991 and 1994 characterised rainfall during 1989–2003, whereas extreme floods, coincident with the strongest ENSO episode on instrumental record up to 2003, occurred in 1997–98 (Bartzke et al., 2018; Ogutu et al., 2008*b*).

The rainfall averaged 785.0 $\pm$ 151.7 mm in the wet season and 213.7 $\pm$ 76.4 mm in the dry season. The total monthly rainfall averaged 82.1 $\pm$ 51.6 mm during 1989–2003. The annual rainfall total over 1965–2003 averaged 1010.1 $\pm$ 187.3 mm, with all months receiving an average of no less than 46 mm of rainfall (Ogutu et al., 2014*b*). Hence, the dry-season nutritional shortfalls for ungulates should be relatively low.

Maximum daily temperatures are the lowest from May to August (Ogutu et al., 2014*b*). The monthly minimum temperature records for 639 months spanning January 1960 to July 2020 for Narok Town situated 75 km northeast of the Mara Reserve averaged 9.2 $\pm$ 2.3 °C (range 0.3 - 17.4 °C). The corresponding monthly maximum temperature averaged 24.9 $\pm$ 2.1 °C (range 19.8 - 30.6 °C). Temperatures are rising in the Mara with the minimum component increasing more steeply than the maximum (Ogutu et al., 2008*b*).

S.1.6 Transformation of variables

The covariates for logit regression models listed in Table 3 of the main text are standardized using various transformations. We standardized the rainfall values by dividing all the values of each rainfall component by its average calculated over July 1989 to December 2003. The minimum and maximum temperatures are standardized to zero mean and unit variance. The covariate "month" in the logit model for $r(t)$ is treated as a numeric variable and is standardized to zero mean and unit variance. The lagged population values are standardized by dividing each by the average computed over July 1989 to December 2003. The transformations applied to the various covariates are specified in Table S4 and in the data

dictionary document named Data_Dictionary.docx in the folder named Data_Dictionary new.

Table S4: List of transformations of the explanatory variables

Covariate	Logit model involved	Transformation
Rainfall components	$r(t)$, $s_q(t)$, $s_h(t)$, $s_a(t)$	Divided by their respective averages
Minimum temperature	$r(t)$, $\phi(t)$	Standardized to zero mean and unit variance
Maximum temperature	$\phi(t)$	Standardized to zero mean and unit variance
Total population size derived from the aerial surveys at different lags	$r(t)$, $s_a(t)$	Divided by its average
Month (treated as numeric variable)	$r(t)$	Standardized to zero mean and unit variance

S.2 Model Formulation Details

The full model is described in Section 3 and Table 2 in the main text. To implement the model in the Bayesian setup, we need to formulate the full likelihood and the posterior or the full conditional distributions of the model parameters.

The forms of the full conditional distributions of the parameters in the population dynamics model are specified in Equations (4)–(27) in this section. Most of the notations used in this section have already been defined in Section 3 of the main text and thus are not defined anew; only notations introduced for the first time are defined in this section.

S.2.1 Full likelihood and full conditional distributions

The full conditional distributions for $Pop(t) = (N(t)$, $G(t,1)$, …, $G(t,6)$, $G(t,1)$, …, $G(t,19)$, $P(t,1), \ldots, P(t,12), F_a(t)), M_a(t), M_b(t))$ can be derived from the full likelihood given in Equation 1 below. The full likelihood and the full conditional distributions in equations 2 to 23 below are strictly conditional on the unknown parameters, the vectors (γ^R, γ^Q, γ^H, γ^A), that underlie $r(t)$, $s_q(t)$, $s_h(t)$, and $s_a(t)$.

The full likelihood can be decomposed into three components as follows. The set of expressions in Equation 1 are derived from the set of distributions specified in Table 2 in the main text.

$$\begin{aligned}\boldsymbol{L}(n,q,h,f,m,N,G,P,M_a,M_b,\mho) &= \boldsymbol{L}_1(n,q,h,f,m \mid N,G,P,M_a,M_b,\mho)\\ &\times \boldsymbol{L}_2(\mho \mid N,G,P,M_a,M_b) \times \boldsymbol{L}_3(N,G,P,M_a,M_b),\end{aligned} \tag{1}$$

where the likelihood for the ground sample count data is

$$\boldsymbol{L}_1(n,q,h,f,m \mid N,G,P,M_a,M_b,\mho) = \prod_{t=1}^{T}\Bigg\{\frac{e^{-\lambda_1(t)}\lambda_1(t)^{n(t)}}{n(t)!} \times \frac{e^{-\lambda_2(t)}\lambda_2(t)^{q(t)}}{q(t)!}$$
$$\times \quad \frac{e^{-\lambda_3(t)}\lambda_3(t)^{h(t)}}{h(t)!} \times \frac{e^{-\lambda_4(t)}\lambda_4(t)^{f(t)}}{f(t)!} \times \frac{e^{-\lambda_5(t)}\lambda_5(t)^{m(t)}}{m(t)!} \times \prod_{\ell=1}^{6}\Big(\frac{e^{-\beta_\ell(t)\lambda_\ell(t)}\lambda_\ell(t)^{\alpha_\ell(t)-1}}{\Gamma(\alpha_\ell)}\Big)\Bigg\},$$

the likelihood for the total population size estimate derived from the aerial survey data using Jolly's (1969) Method 2 is

$$\boldsymbol{L}_2(\mho \mid N,G,P,M_a,M_b) = \prod_{\ell=1}^{T}\Bigg\{\frac{\Big(e^{-\lambda_t^T}\Big)\Big(\lambda_t^{\mho_t}\Big)}{\mho_t!} \times \frac{\Big(e^{-\frac{K_tB_t}{\sigma_T^2}\mho_t}\Big)\Big(\mho_t^{(\frac{K_tB_t}{\sigma_T})^2-1}\Big)}{\Gamma(\frac{K_tB_t}{\sigma_T})}\Bigg\} \times e^{-\frac{(\Psi_t-\mho_t)^2}{2\sigma_\Psi^2}}, \tag{2}$$

and the likelihood for the latent, hidden or true states or population sizes is

$$
\begin{aligned}
&\boldsymbol{L}_3(N,G,P,M_a,M_b) = \prod_{t=1}^{T}\Bigg\{ \binom{P(t,11)+P(t,12)}{N(t)} r(t)^N(t)(1-r(t))^{P(t,11)+P(t,12)-N(t)} \\
&\times \binom{N(t-1)}{G(t,1)} s_q^{G(t,1)}(1-s_q)^{N(t-1)-G(t,1)} \\
&\times \binom{G(t-1,19)}{F_a(t),M_a(t)} (c(t)s_a(t))^{F_a(t)}((1-c(t))s_a(t))^{M_a(t)}(1-s_a(t))^{G(t-1,19)-F_a(t)-M_a(t)} \\
&\times \prod_{\ell=1}^{19} \binom{G(t-1,k-1)}{G(t,k)} p_\ell(t)^{G(t,k)}(1-p_\ell(t))^{G(t-1,k-1)-G(t,k)} \\
&\times \binom{M(t-1)}{M_b(t)} s_a^{M_b}(1-s_a)^{M(t-1)-M_b} \times \binom{N(t-1)}{P(t,1)} s_a(t)^{P(t,1)}(1-s_a(t))^{N(t-1)-P(t,1)} \\
&\times \prod_{\ell=1,\ell\neq 3}^{11} \binom{P(t-1,\ell-1)}{P(t,\ell)} s_a(t)^{P(t,\ell)}(1-s_a(t))^{P(t-1,\ell-1)-P(t,\ell)} \\
&\times \binom{P(t-1,11)+P(t-1,12)-N(t-1)}{P(t,12)} s_a(t)^{P(t,12)}(1-s_a(t))^{P(t-1,11)+P(t-1,12)-N(t-1)-P(t,12)} \\
&\times \binom{P(t-1,2)+F_a(t-1)}{P(t,3)} s_a(t)^{P(t,3)}(1-s_a(t))^{P(t-1,2)+F_a(t-1)-P(t,3)} \Bigg\},
\end{aligned}
\tag{3}
$$

where $p_\ell(t) = s_h(t)$ when $1 \leq \ell \leq 6$ and $p_\ell(t) = s_a(t)$ when $1 \leq \ell \leq 19$.

The conditional posterior distributions for the latent (hidden or true) states or population sizes from time $t = 1, \ldots, T$ are provided in equations 4 to 15.

For deriving the posterior distributions, first define, $f_1(Pop(t))$ and $f_2(Npop(t))$ (as defined in Table 3 in the main text, $Pop(t)$, the vectors of all the population components at time t, and $Npop(t)$, the vectors of all the population components at time (t – 8)) as

$$
\begin{aligned}
f_1(Pop(t)) &= (s_a(t+1))^{\sum_{k=1}^{12} P(t+1,k)+F_a(t+1)+M_a(t+1)+M_b(t+1)} \times \\
&\quad (1-s_a(t+1))^{G(t,19)+\sum_{k=1}^{12} P(t,k)-\sum_{k=1}^{12} P(t+1,k)-F_a(t+1)-M_a(t+1)-M_b(t+1)}, \\
&\quad \text{if } t < 174 \\
&= 1, t = 174.
\end{aligned}
$$

$$
\begin{aligned}
f_2(Npop(t)) &= (r(t+8))^{N(t+8)}(1-r(t+8))^{P(t+8,11)+P(t+8,12)-N(t+8)}, \text{if } t < 166 \\
&= 1, t \geq 166.
\end{aligned}
$$

S.2.2 Posterior or full conditional distributions

Write $A \setminus B$ as the difference between sets A and B.

S.2.2.1 Conditionals for states, t $\geq$1

The full conditional distributions for newborns $N(t)$, for $t \geq 1$, are then given by

$$
\begin{aligned}
[N(t) \quad | \quad (Pop(t) \setminus N(t)), \boldsymbol{\gamma}] = & \binom{P(t,11)+P(t,12)}{N(t)}(r(t))^{N(t)} \times \\
& (1-r(t))^{P(t,11)+P(t,12)-N(t)} \times \\
& \binom{N(t)}{G(t,1)}(s_q(t))^{G(t,1)}(1-s_q(t))^{N(t)-G(t,1)} \times \\
& \binom{P(t,11)+P(t,12)-N(t)}{P(t+1,12)} \times \binom{N(t)}{P(1,12)} \times \\
& e^{-\beta_1(t)\lambda_1(t)}\lambda_1(t)^{\alpha_1(t)} \times \frac{\beta_1(t)^{\alpha_1(t)}}{\Gamma(\alpha_1(t))} \times f_1(Pop(t)) \times f_2(Npop(t)). \qquad (4)
\end{aligned}
$$

The full conditional distributions for quarters, $G(t,k)$, for $2 \leq k \leq 6$, and $1 \leq t \leq 174$ vary with k as shown below -

For $k = 2$,

$$
\begin{aligned}
[G(t,2) \quad | \quad (Pop(t) \setminus G(t,2)), \boldsymbol{\gamma}] = & \binom{N(t-1)}{G(t,2)}(s_q(t))^{G(t,2)}(1-s_q(t))^{N(t-1)-G(t,2)} \times \\
& \binom{G(t,2)}{G(t+1,3)}(s_q(t+1))^{G(t+1,3)}(1-s_q(t+1))^{N(t)-G(t+1,2)} \times \\
& e^{-\beta_2(t)\lambda_2(t)}\lambda_2(t)^{\alpha_2(t)} \times \frac{\beta_2(t)^{\alpha_2(t)}}{\Gamma(\alpha_2(t))} \times f_1(Pop(t)) \times f_2(Npop(t)); \qquad (5)
\end{aligned}
$$

for $3 \leq k \leq 6$,

$$
\begin{aligned}
[G(t,k) \quad | \quad (Pop(t) \setminus G(t,k)), \boldsymbol{\gamma}] = & \binom{G(t-1,k-1)}{G(t,k)} (s_q(t))^{G(t,k)} \\
& (1-s_q(t))^{G(t-1,k-1)-G(t,k)} \times \\
& \binom{G(t,k)}{G(t+1,k+1)} (s_q(t+1))^{G(t+1,k+1)} (1-s_q(t+1))^{G(t,k)-G(t+1,k+1)} \times \\
& e^{-\beta_2(t)\lambda_2(t)} \lambda_2(t)^{\alpha_2(t)} \times \frac{\beta_2(t)^{\alpha_2(t)}}{\Gamma(\alpha_2(t))} \times f_1(Pop(t)) \times f_2(Npop(t)); \qquad (6)
\end{aligned}
$$

The full conditional distributions for half-yearlings, $G(t,k)$, for $7 \leq k \leq 19$, and $1 \leq t \leq 174$ are given below.

$$
\begin{aligned}
[G(t,\ell) \quad | \quad (Pop(t) \setminus G(t,\ell)), \boldsymbol{\gamma}] = & \binom{G(t-1,\ell-1)}{G(t,\ell)} (s_h(t))^{G(t,\ell)} \times \\
& (1-s_h(t))^{G(t-1,\ell-1)-G(t,\ell)} \times \\
& \binom{G(t,\ell)}{G(t+1,\ell+1)} (s_h(t+1))^{G(t+1,\ell+1)} (1-s_h(t+1))^{G(t,\ell+1)-G(t+1,\ell)} \times \\
& e^{-\beta_3(t)\lambda_3(t)} \lambda_3(t)^{\alpha_3(t)} \times \frac{\beta_3(t)^{\alpha_3(t)}}{\Gamma(\alpha_3(t))} \times f_1(Pop(t)) \times f_2(Npop(t)); \qquad (7)
\end{aligned}
$$

for $\ell = 19$,

$$
\begin{aligned}
[G(t,19) \quad | \quad (Pop(t) \setminus G(t,19)), \boldsymbol{\gamma}] = & \binom{G(t-1,18)}{G(t,19)} (s_h(t))^{G(t,19)} \times \\
& (1-s_h(t))^{G(t-1,18)-G(t,19)} \binom{G(t,19)}{F_a(t+1), M_a(t+1)} \times \\
& e^{-\beta_3(t)\lambda_3(t)} \lambda_3(t)^{\alpha_3(t)} \times \frac{\beta_3(t)^{\alpha_3(t)}}{\Gamma(\alpha_3(t))} \times f_1(Pop(t)) \times f_2(Npop(t)). \qquad (8)
\end{aligned}
$$

The full conditional distributions for adult females are given by

$$
\begin{aligned}
[F_a(t) \quad | \quad (Pop(t) \setminus F_a(t)), \boldsymbol{\gamma}] = &\binom{G(t-1,19)}{F_a(t), M_a(t)} (\phi \times s_a(t))^{F_a(t)} \times \\
&((1-\phi) \times s_a(t))^{M_a(t)} \times (1-s_a(t))^{G(t-1,19)-F_a(t)-M_a(t)} \times \\
&\binom{F_a(t)+P(t,3)}{P(t+1,4)} \times e^{-\beta_4(t)\lambda_4(t)} \lambda_4(t)^{\alpha_4(t)} \times \frac{\beta_4(t)^{\alpha_4(t)}}{\Gamma(\alpha_4(t))} \times \\
&f_1(Pop(t)) \times f_2(Npop(t)); \qquad (9)
\end{aligned}
$$

for $k = 1$,

$$
\begin{aligned}
[P(t,1) \quad | \quad (Pop(t) \setminus P(t,1)), \boldsymbol{\gamma}] = &\binom{N_{t-1}}{P(t,1)} (s_a(t))^{P(t,1)} (1-s_a(t))^{N_{t-1}-P(t,1)} \times \\
&\binom{M_a(t)+M_b(t)}{M_b(t+1)} \times e^{-\beta_4(t)\lambda_4(t)} \lambda_4(t)^{\alpha_4(t)} \times \frac{\beta_4(t)^{\alpha_4(t)}}{\Gamma(\alpha_4(t))} \\
&f_1(Pop(t)) \times f_2(Npop(t)); \qquad (10)
\end{aligned}
$$

For $2 \leq k \leq 10$,

$$
\begin{aligned}
[P(t,k) \quad | \quad (Pop(t) \setminus P(t,k)), \boldsymbol{\gamma}] = &\binom{Prev(t,k)}{P(t,k)} (s_a(t))^{P(t,k)} (1-s_a(t))^{Prev(t,k)-P(t,k)} \times \\
&\binom{Next(t,k)}{P(t+1,k+1)} \times e^{-\beta_4(t)\lambda_4(t)} \lambda_4(t)^{\alpha_4(t)} \times \frac{\beta_4(t)^{\alpha_4(t)}}{\Gamma(\alpha_4(t))} \times \\
&f_1(Pop(t)) \times f_2(Npop(t)); \qquad (11)
\end{aligned}
$$

where

$$
\begin{aligned}
Prev(t,k) &= P(t-1,k-1), \text{if } k \neq 4 \\
&= P(t-1,3) + F_a(t-1), \text{if } k = 4 \\
Next(t,k) &= P(t,k), \text{if } k \neq 3 \\
&= P(t,3) + F_a(t), \text{if } k = 3.
\end{aligned}
$$

For $k = 11$,

$$\begin{aligned}[P(t,11) \quad | \quad Pop(t) - P(t,11)] = &\binom{P(t-1,10)}{P(t,11)}(s_a(t))^{P(t,11)}(1-s_a(t))^{P(t-1,10)-P(t,11)} \times \\ &\binom{P(t,11)+P(t,12)}{N(t)}(r(t))^{N(t)}(1-r(t))^{P(t,11)+P(t,12)-N(t)} \times \\ &\binom{P(t,11)+P(t,12)-N(t)}{P(t+1,12)}e^{-\beta_4(t)\lambda_4(t)}\lambda_4(t)^{\alpha_4(t)} \times \frac{\beta_4(t)^{\alpha_4(t)}}{\Gamma(\alpha_4(t))} \times \\ &f_1(Pop(t)) \times f_2(Npop(t)); \end{aligned} \tag{12}$$

for $k = 12$,

$$\begin{aligned}[P(t,12) \quad | \quad Pop(t) - P(t,12)] = &\binom{P(t-1,11)+P(t-1,12)}{P(t,12)} \times \\ &(s_a(t))^{P(t,12)}(1-s_a(t))^{P(t-1,11)+P(t-1,12)-P(t,12)} \times \\ &\binom{P(t,11)+P(t,12)}{N(t)}(r(t))^{N(t)}(1-r(t))^{P(t,11)+P(t,12)-N(t)} \times \\ &\binom{P(t,11)+P(t,12)-N(t)}{P(t+1,12)} \times e^{-\beta_4(t)\lambda_4(t)}\lambda_4(t)^{\alpha_4(t)} \times \frac{\beta_4(t)^{\alpha_4(t)}}{\Gamma(\alpha_4(t))} \times \\ &f_1(Pop(t)) \times f_2(Npop(t)). \end{aligned} \tag{13}$$

For adult males $M(t) = M_a(t) + M_b(t)$, where $M_a(t)$ is the number of males out of $G(t-1,19)$ half-yearlings present at time $t-1$ that join the adult age class at time t. $M_b(t)$ is the number of surviving adult males at time t out of $M(t-1)$ adult males present at time $t-1$. The full conditional distributions for $M_b(t)$ and $M_a(t)$ are given below.

$$\begin{aligned}[M_b(t) \quad | \quad (Pop(t) \setminus M_b(t)), \boldsymbol{\gamma}] = &\binom{M_b(t-1)+M_a(t-1)}{M_b(t)} \times \\ &(s_a(t))^{M_b(t)}(1-s_a(t))^{M_b(t-1)+M_a(t-1)-M_b(t)} \times \\ &\binom{M_b(t)+M_a(t)}{M_b(t+1)} \times e^{-\beta_4(t)\lambda_4(t)}\lambda_4(t)^{\alpha_4(t)} \times \frac{\beta_4(t)^{\alpha_4(t)}}{\Gamma(\alpha_4(t))} \times \\ &f_1(Pop(t)) \times f_2(Npop(t)), \end{aligned} \tag{14}$$

$$\begin{aligned}[M_a(t) \quad | \quad (Pop(t) \setminus M_a(t)), \boldsymbol{\gamma}] = & \binom{G(t-1,19)}{F_a(t), M_a(t)} (\phi \times s_a(t))^{F_a(t)} \times \\ & ((1-\phi) \times s_a(t))^{M_a(t)} \times (1 - s_a(t))^{G(t-1,19)-F_a(t)-M_a(t)} \times \\ & \binom{M_a(t) + M_b(t)}{M_b(t+1)} \times e^{-\beta_4(t)\lambda_4(t)} \lambda_4(t)^{\alpha_4(t)} \times \frac{\beta_4(t)^{\alpha_4(t)}}{\Gamma(\alpha_4(t))} \times \\ & f_1(Pop(t)) \times f_2(Npop(t)). \end{aligned} \quad (15)$$

S.2.2.2 Conditionals for initial states (t = 0)

The conditional posterior distributions for the latent (hidden or true) states or population sizes at time $t = 0$ are specified in equations 16 to 23. The full conditional distributions for the initial states $Pop_0 = (N(0), G(0,1), \ldots, G(0,6), H(0,1), \ldots, H(0,19), P(0,1), \ldots, P(0,12), M(0))$ can be similarly derived as above. The prior distributions for the initial states are specified in Section 3.11 of the main text and so are not reproduced here. Without loss of generality, we denote the prior distribution of any state by $\pi(\cdot)$. Before specifying the forms of the full conditional distributions for the initial states we first define

$$\begin{aligned} f_0 \quad = \quad & (s_a(1))^{\sum_{k=1}^{12} P(1,k)+F_a(1)+M_a(1)+M_b(1)} \times \\ & (1 - s_a(1))^{G(0,19)+\sum_{k=1}^{12} P(0,k) - \sum_{k=1}^{12} P(1,k) - F_a(1) - M_a(1) - M_b(1)} \end{aligned}$$

$$h_0(Npop(t)) \quad = \quad (r(8))^{N(8)} (1 - r(8))^{P(8,11)+P(8,12)-N(8)}.$$

Since they are present in all equations 16–20 we introduce notations for these two complex forms to avoid writing them repeatedly.

The full conditional distribution for newborns, $N(0)$, is then given by

$$\begin{aligned}[N(0) \quad | \quad (Pop_0 \setminus N(0)), \boldsymbol{\gamma}] = & \binom{P(0,11)+P(0,12)}{N(0)} \times \\ & (r(0))^{N(0)}(1-r(0))^{P(0,11)+P(0,12)-N(0)} \times \\ & \binom{N(0)}{G(1,1)}(s_q(1))^{G(1,1)}(1-s_q(1))^{N(0)-G(1,1)} \times \\ & \binom{P(0,11)+P(0,12)-N(0)}{P(1,12)} \times \binom{N(0)}{F_a(1,1)} \times \\ & f_0 \times h_0 \times \pi(N(0)) \end{aligned} \tag{16}$$

The full conditional distributions for quarters, $G(0,k)$, $1 \leq k < 6$, are given by

$$\begin{aligned}[G(0,k) \quad | \quad (Pop_0 \setminus G(0,k)), \boldsymbol{\gamma}] = & \binom{G(0,k)}{G(1,k+1)}(s_q(1))^{G(1,k+1)} \times \\ & (1-s_q(1))^{G(0,k)-G(1,k+1)} \times f_0 \times h_0 \times \pi(G(0,k)). \end{aligned} \tag{17}$$

For $k = 6$,

$$\begin{aligned}[G(0,6) \quad | \quad (Pop_0 \setminus G(0,k)), \boldsymbol{\gamma}] = & \binom{G(0,k)}{G(1,1)}(s_h(1))^{G(1,1)} \times \\ & (1-s_h(1))^{G(0,6)-G(1,1)} \times f_0 \times h_0 \times \pi(G(0,6)) \end{aligned} \tag{18}$$

The full conditional distributions for half-yearlings, $G(0,k)$, $7 \leq k < 19$, are given by

$$\begin{aligned}[G(0,k) \quad | \quad (Pop_0 \setminus G(0,k)), \boldsymbol{\gamma}] = & \binom{G(0,k)}{G(1,k+1)}(s_h(1))^{G(1,k+1)} \times \\ & (1-s_h(1))^{G(0,k)-G(1,k+1)} \times f_0 \times h_0 \times \pi(G(0,k)) \end{aligned} \tag{19}$$

$$\begin{aligned}[G(0,19) \quad | \quad (Pop_0 \setminus G(0,19)), \boldsymbol{\gamma}] = & \binom{G(0,19)}{F_a(1), M_a(1)}(\phi \times s_a(1))^{F_a(1)} \times \\ & ((1-\phi) \times s_a(1))^{M_a(1)} \times (1-s_a(1))^{G(0,19)-F_a(1)-M_a(1)} \times \\ & f_0 \times h_0 \times \pi(G(0,19)) \end{aligned} \tag{20}$$

The full conditional distributions for adult females that gave birth k months before time t = 0, $P(0,k)$, $1 \leq k < 11$, are given by

$$[P(0,k) \quad | \quad (Pop_0 \setminus P(0,k)), \boldsymbol{\gamma}] = \binom{P(0,k)}{P(1,k+1)} \times f_0 \times h_0 \times \pi(P(0,k)); \tag{21}$$

for $k = 11$,

$$\begin{aligned}
[P(0,11) \quad | \quad (Pop_0 \setminus P(0,11)), \boldsymbol{\gamma}] = & \binom{P(0,11) + P(0,12)}{N(0)} \times \\
& (r(0))^{N(0)} (1 - r(0))^{P(0,11)+P(0,12)-N(0)} \times \\
& \binom{P(0,11) + P(0,12) - N(0)}{P(1,12)} \times f_0 \times h_0 \times \pi(P(0,11)); \qquad (22)
\end{aligned}$$

for $k = 12$,

$$\begin{aligned}
[P(0,12) \quad | \quad (Pop_0 \setminus P(0,12)), \boldsymbol{\gamma}] = & \binom{P(0,11) + P(0,12)}{N(0)} \times \\
& (r(0))^{N(0)} (1 - r(0))^{P(0,11)+P(0,12)-N(0)} \times \\
& \binom{P(0,11) + P(0,12) - N(0)}{P(1,12)} \times f_0 \times h_0 \times \pi(P(0,11)). \qquad (23)
\end{aligned}$$

S.2.2.3 Conditionals for parameters

The posterior distributions of the regression parameters $\boldsymbol{\gamma}^A$, $\boldsymbol{\gamma}^H$, $\boldsymbol{\gamma}^Q$, $\boldsymbol{\gamma}^R$ and $\boldsymbol{\gamma}^S$ are specified in equations 24 to 28.

The full conditional distribution for the vector of adult survival parameters, $\boldsymbol{\gamma}^A$, is given by

$$\begin{aligned}
[\boldsymbol{\gamma}^A \quad | \quad Pop(t), Pop_0] = & \left\{ \prod_{t=1}^{174} (s_a(t))^{\sum_{k=1}^{12} P(t,k) + F_a(t) + M_a(t)} \right\} \times \\
& \left\{ (1 - s_a(1))^{G(0,19) + \sum_{k=1}^{12} P(0,k) - \sum_{k=1}^{12} P(1,k)} \right\} \times \\
& \left\{ \prod_{t=2}^{174} (1 - s_a(t))^{G(t-1,19) + \sum_{k=2}^{12} P(t-1,k) - \sum_{k=1}^{12} P(t,k) - F_a(t) - M_a(t)} \right\} \times \\
& \pi(\boldsymbol{\gamma}^A) \qquad (24)
\end{aligned}$$

The full conditional distribution for the vector of birth recruitment parameters, $\boldsymbol{\gamma}^R$, is

given by

$$[\boldsymbol{\gamma}^R \mid Pop(t), Pop_0] = \left\{(r(0))^{N(0)} \times (1 - r(0))^{P(0,11)+P(0,12)-N(0)}\right\} \times \left\{\prod_{t=1}^{174}(r(t))^{N(t)} \times (1 - r(t))^{P(t,11)+P(t,12)-N(t)}\right\} \times \pi(\boldsymbol{\gamma}^R) \tag{25}$$

The full conditional distribution for the vector of survival parameters for the quarter age class, $\boldsymbol{\gamma}^Q$, is given by

$$[\boldsymbol{\gamma}^Q \mid Pop(t), Pop_0] = \left\{(s_q(1))^{\sum_{k=1}^{6} G(1,k)} \times (1 - s_q(1))^{N(0)+\sum_{k=1}^{5} G(1,k)-\sum_{k=1}^{6} G(2,k)}\right\} \times \left\{\prod_{t=2}^{174}(s_q(t))^{\sum_{k=1}^{6} G(t,k)} \times (1 - s_q(t))^{N(t-1)+\sum_{k=1}^{5} G(t-1,k)-\sum_{k=1}^{6} G(t,k)}\right\} \times \pi(\boldsymbol{\gamma}^Q) \tag{26}$$

The full conditional distribution for the vector of survival parameters for half-yearlings, $\boldsymbol{\gamma}^H$, is given by

$$[\boldsymbol{\gamma}^H \mid Pop(t), Pop_0] = \left\{(s_h(1))^{\sum_{k=7}^{19} G(1,k)} \times (1 - s_h(1))^{G(0,6)+\sum_{k=7}^{18} G(1,k)-\sum_{k=7}^{19} G(2,k)}\right\} \times \left\{\prod_{t=2}^{174}(s_h(t))^{\sum_{k=7}^{19} G(t,k)} \times (1 - s_q(t))^{G(t-1,6)+\sum_{k=7}^{18} G(t-1,k)-\sum_{k=7}^{19} G(t,k)}\right\} \times \pi(\boldsymbol{\gamma}^Y) \tag{27}$$

The full conditional distribution for the vector of parameters for adult sex-ratio, $\boldsymbol{\gamma}^S$, is given by

$$[\boldsymbol{\gamma}^S \mid Pop(t)] = \left\{\prod_{t=1}^{174}(\phi(t))^{F_a(t)} \times (1 - \phi(t))^{M_a(t)}\right\} \times \pi(\boldsymbol{\gamma}^S) \tag{28}$$

S.2.3 Determining the initial states for running the MCMC chain

Estimation of the parameters of the topi population dynamics model described in Table 2 is done by simulating from the posterior distribution of the parameters described in Section

S.2.2.1. We used the MCMC technique for the simulations. According to the theory of MCMC simulation, we need an initial estimate of the parameter set ($Pop(t)$, Pop_0, γ^R, γ^Q, γ^H, γ^A, etc.) to initialize the MCMC chain. The initial estimates are sequentially updated using the TMCMC (Section S.3.1) for a large number of iterations. The theory of the TMCMC (Dutta and Bhattacharya, 2014) shows that after convergence the chain is independent of the impact of the initial choice. Even so, for the sake of completeness and those who may wish to use a similar modeling approach, we explain in detail how we calculated the initial chioces for these parameters. To void any possible confusion it is essential to stress that these estimates are used solely to start the MCMC chain, and not for any prediction or inference and that the final estimates from the converged MCMC chain are expected to be free from or not to be influenced by the initial choices. Moreover, it is critical to emphasize that the MCMC theory argues against double use of the same data so that the initial estimates should ideally be based solely on data that will not be used in the MCMC to fit the population dynamics model. In our case, there were no other data with a structure that matches our ground sample count data for June 1989. This left us with no other option but to rely on the available data to derive the initial state estimates. To gain a better understanding of how this is actually implemented in the model please refer to the R codes specified in the Supplementary Materials Section S.6.

The logit functional forms and the initial values of (γ^R, γ^Q, γ^H, γ^A, etc.) are determined using logistic regression in SAS PROC GLIMMIX as described in Section S.6. Note that this exercise also gives us initial estimates for ($r(t)$, $s_q(t)$, $s_h(t)$, $s_a(t)$ and $\phi(t)$). We first describe how we determine the initial estimates of ($Pop(t)$, Pop_0). Recall that in our data set time t = 0 corresponds to the month of June 1989.

The equations used to calculate the initial values are summarized in the following steps.

1. Take the ground vehicle count data for month t and calculate the proportion of animals in each age and sex class.

We let

$$
\begin{aligned}
&\text{The sampled total population size based on the ground counts} = \\
&\tilde{\varphi}(t) = n(t) + q(t) + h(t) + f(t) + m(t) \\
&\text{The sample population proportion of newborns} = \tilde{p}_r(t) = \frac{n(t)}{\tilde{\varphi}(t)} \\
&\text{The sample population proportion of quarters} = \tilde{p}_q(t) = \frac{q(t)}{\tilde{\varphi}(t)} \\
&\text{The sample population proportion of half-yearlings} = \tilde{p}_h(t) = \frac{h(t)}{\tilde{\varphi}(t)} \\
&\text{The sample population proportion of adult females} = \tilde{p}_f(t) = \frac{f(t)}{\tilde{\varphi}(t)} \\
&\text{The sample population proportion of adult males} = \tilde{p}_m(t) = \frac{m(t)}{\tilde{\varphi}(t)} \qquad (29)
\end{aligned}
$$

2. Calculate the monthly average sample proportions for each age or sex class.

$$
\begin{aligned}
&\text{Average sample population proportion of newborns} = \bar{A}_r(\ell) = \frac{1}{N(\ell)} \sum_{\mathbb{H}E(\ell)} \tilde{p}_r(t) \\
&\text{Average sample population proportion of quarters} = \bar{A}_q(\ell) = \frac{1}{N(\ell)} \sum_{\mathbb{H}E(\ell)} \tilde{p}_q(t) \\
&\text{Average sample population proportion of half-yearlings} = \bar{A}_h(\ell) = \frac{1}{N(\ell)} \sum_{\mathbb{H}E(\ell)} \tilde{p}_h(t) \\
&\text{Average sample population proportion of adult females} = \bar{A}_f(\ell) = \frac{1}{N(\ell)} \sum_{\mathbb{H}E(\ell)} \tilde{p}_f(t) \\
&\text{Average sample population proportion of adult males} = \bar{A}_m(\ell) = \frac{1}{N(\ell)} \sum_{\mathbb{H}E(\ell)} \tilde{p}_m(t), \qquad (30)
\end{aligned}
$$

where $N(\ell)$ is the number of time points t ($t = 1, \ldots, 174$) which corresponds to month ℓ ($\ell = 1, \ldots, 12$) and $\mathbb{H}E(\ell)$ denotes the set of time points t which are in month ℓ.

3. Take the total population size estimates derived from the aerial survey data using Jolly's Method2 and impute the missing values by the method of Piepho and Ogutu

(2007) as implemented in Ogutu et al., (2014*b*, 2016). Let $\hat{\chi}(t)$ be the estimated total population size derived from the aerial counts at time t.

4. Calculate the monthly average of the total population size estimated from the aerial survey data over all the months from July 1989 to December 2003.

$$\bar{\chi}(\ell) = \frac{1}{N(\ell)} \sum_{\mathbb{H}E(\ell)} \hat{\chi}(t) \tag{31}$$

5. Multiply the monthly average of the total population size at time t = 0 with the sample proportion for the different age and sex classes. This gives an estimate of the total population size for all the age and sex groups - $N(0)$, $Q(0)$, $H(0)$, $F(0)$, $M(0)$ which can then be used as starting values for the MCMC chain. Take ℓ = 6 and calculate the following.

$$\begin{aligned}
&\text{Initial population size of newborns} = \hat{N}(0) = \bar{\chi}(\ell)\bar{A}_r(\ell)\\
&\text{Initial population size of quarters} = \hat{Q}(0) = \bar{\chi}(\ell)\bar{A}_q(\ell)\\
&\text{Initial population size of half-yearlings} = \hat{H}(0) = \bar{\chi}(\ell)\bar{A}_h(\ell)\\
&\text{Initial population size of adult females} = \hat{F}(0) = \bar{\chi}(\ell)\bar{A}_f(\ell)\\
&\text{Initial population size of adult males} = \hat{M}(0) = \bar{\chi}(\ell)\bar{A}_m(\ell).
\end{aligned} \tag{32}$$

These estimates serve as the starting values, Pop_0, for the MCMC chain.

6. Following the distributional specifications in Table 2 in the main text and using the initial estimates of probabilities we can split $F(t)$ into $P(t, k)$, $k = 1, \ldots, 12$ and $F_a(t)$. Note that $P(t, k)$ is the number of females that gave birth k months ago and survived for k months. For our data, t = 0 means the month of June or ℓ = 6. We approximate the relative proportions of breeding females $P(0, 1) : P(0, 2) : P(0, 3) : P(0, 4) : P(0, 5) : P(0, 6) : P(0, 7) : P(0, 8) : P(0, 9) : P(0, 10) : P(0, 11) : P(0, 12)$ by $\bar{A}_r(5) : \bar{A}_r(4) : \bar{A}_r(3) : \bar{A}_r(2) : \bar{A}_r(1) : \bar{A}_r(12) : \bar{A}_r(11) : \bar{A}_r(10) : \bar{A}_r(9) : \bar{A}_r(8) : \bar{A}_r(7) : \bar{A}_r(6)$, respectively. Upon multiplying these proportions by $\hat{F}(0)$, we can get estimates for $P(0, k)$. We then estimate $F_a(0)$ = $\hat{F}(0)$ - $\sum_{k=1}^{12} P(0, k)$.

7. A newborn will remain in the quarter age class for the next 5 months (k = $2, \ldots, 6$) and then join and remain in the half-yearling class for 13 months (k = $7, \ldots, 19$). This information can be used together with the quantities defined in point (2) above to obtain estimates of initial values for the total population sizes of the quarter-size topi aged 2 to 6 months, half-yearling topi aged 7 to 18 months and half-yearlings joining the adult age class and aged 19 months using the following expressions:

   Quarter size topi aged 2 to 6 months:
   $G(0, k) = 1.33 \times \bar{A}_r(6 - k + 1) \times (\bar{s}_q(6 - k + 1))^{k-1}$, $k = 2, \ldots, 6$;
   Half-yearling topi aged 7 to 18 months:
   $G(0, 6 + k) = 1.2 \times \bar{A}_q(13 - k) \times (\bar{s}_h(13 - k))^k$, $k = 1, \ldots, 12$;
   Half-yearling topi joining the adult class:
   $G(0, 19) = 1.2 \times \bar{A}_q(12) \times (\bar{s}_h(12))^{13}$,

   (33)

   where $\bar{s}_q(\ell) = \frac{1}{N(\ell)} \sum_{\mathbb{H}E(\ell)} \hat{s}_q(t)$ and $\bar{s}_h(\ell) = \frac{1}{N(\ell)} \sum_{\mathbb{H}E(\ell)} \hat{s}_h(t)$.

8. We used multipliers - 1.2 and 1.33 for quarters and half-yearlings, respectively. These are used to adjust for sightability bias, loss to predation or both, and also serve to counteract the effect of underestimation of the actual number of newborns. We tried various other mutlipliers close to these values and obtained comparable results.

9. This approach to estimating initial numbers of individuals in the various age classes will only work well when the starting year is not an year with climatic extremes. It is therefore advisable to carefully consider whether the initial year can be considered extreme and its possible impact on the ecosystem and the study population before deciding how to estimate the initial values for the total population sizes for the different age and sex classes.

S.3 Model fitting, diagnostics and assessment

Model fitting in the Bayesian framework is done after formulating the model, specifying the prior and posterior distributions, the likelihood, the initial parameter and state values, and using a simulation method to generate possible values for the model parameters and states. After fitting the model and obtaining posterior samples, it is essential to assess the model's performance. This helps to assess and establish the model's robustness. Lastly, important features of the model are explored, for example, by examining the relationships between the model parameters and assessing their significance. Two recent reviews or tutorial-type papers on state space models, which focus largely on ecological settings, provide excellent templates of the salient steps in state space model fitting and assessment (Auger-Méthé et al., 2021; Newman et al., 2023).

S.3.1 TMCMC step

The beginning of each Metroplois-Hastings step for the $\boldsymbol{\gamma}$'s (specified in Table 4 of the main text) requires a new value from a proposal distribution. When the dimension of the parameter vector is large, then finding a suitable proposal distribution (Robert, 2015) can be exceedingly challenging. The TMCMC circumvents this problem by updating a functional of $\boldsymbol{\gamma}$'s of reduced dimension to improve mixing of the chain. To do so, we can define a d-dimensional (d denotes the dimension of $\boldsymbol{\gamma}^R$, $\boldsymbol{\gamma}^Q$, $\boldsymbol{\gamma}^H$ or $\boldsymbol{\gamma}^A$) random vector $\mathbf{b} = \{b_1, b_2, \ldots, b_d\}$, such that $b_i = 1$ with probability p and $b_i = -1$ otherwise.

Now, suppose that at the ℓ-th MCMC step the value of the parameter vector is $\boldsymbol{\gamma}^{(\ell)}$, then the next sequential update $\boldsymbol{\gamma}^{(\ell+1)}$ is given by

$$\boldsymbol{\gamma}^{(\ell+1)} = \boldsymbol{\gamma}^{(\ell)} + (\mathbf{b} * \boldsymbol{c})\epsilon, \tag{34}$$

where $*$ denotes the inner vector product, $\boldsymbol{c} = (c_1, \ldots, c_d)$, $c_i > 0$, for all i, is a vector of positive constants and $\epsilon \sim$ Normal(0, 1) such that $\epsilon > 0$.

The preceding transformation is closely related to a random walk. The choices of c and p are determined by trial and error. Suppose we choose p to be 0.5. Then, we can choose c by observing that $\boldsymbol{c}$ is the standard deviation of the components of $\boldsymbol{\gamma}^{(\ell+1)}$ given $\boldsymbol{\gamma}^{(\ell)}$. Next, we set a pilot run of 500,000 for $\boldsymbol{\gamma}$'s, discarding the initial 200,000 runs, and

calculate the standard deviation of the MCMC samples of the parameter components to be used as c_i, $i = 1, \ldots, d$.

The final model is run for 3,000,000 iterations after discarding the initial 1,000,000 as burn-in. Since plotting 3,000,000 values would result in a very large file, we plotted every 100-th value from the MCMC iterations for a total of 30,000 values. However, we used the full set of 3,000,000 values in all the analyses. Trace plots of some of the $\boldsymbol{\gamma}$'s are shown in Supplementary Figures S13–S14. These plots indicate good mixing of the chains.

To assess if the chains are highly autocorrelated, we calculated effective sample sizes using the 30,000 subsamples obtained from the MCMC chain of 3,000,000 by keeping every 100th sample for ten randomly selected parameters and states and found these to range between 1250 and 5000 for each parameter and state.

A prior that matches the posterior distribution typically indicates that the data had no influence on the parameter estimation and that the parameter is not estimable. To check for parameter estimability, we therefore compared the prior and posterior distributions by examining the density of the prior distributions of 10 randomly selected parameters against the densities of their corresponding posteriors from the MCMC simulations. The results revealed strong divergences between the prior and posterior densities. To formalize this observation, we conducted Kolmogorov-Smirnov tests for the prior and posterior densities. In all cases, the test confirmed that the two densities were significantly different for all the parameters (p-value $< 2.22 \times 10^{-6}$ for all tests). Therefore, we conclude that the data provides no evidence that the priors had strong influences on the posterior distributions. This suggests that the parameters are estimable.

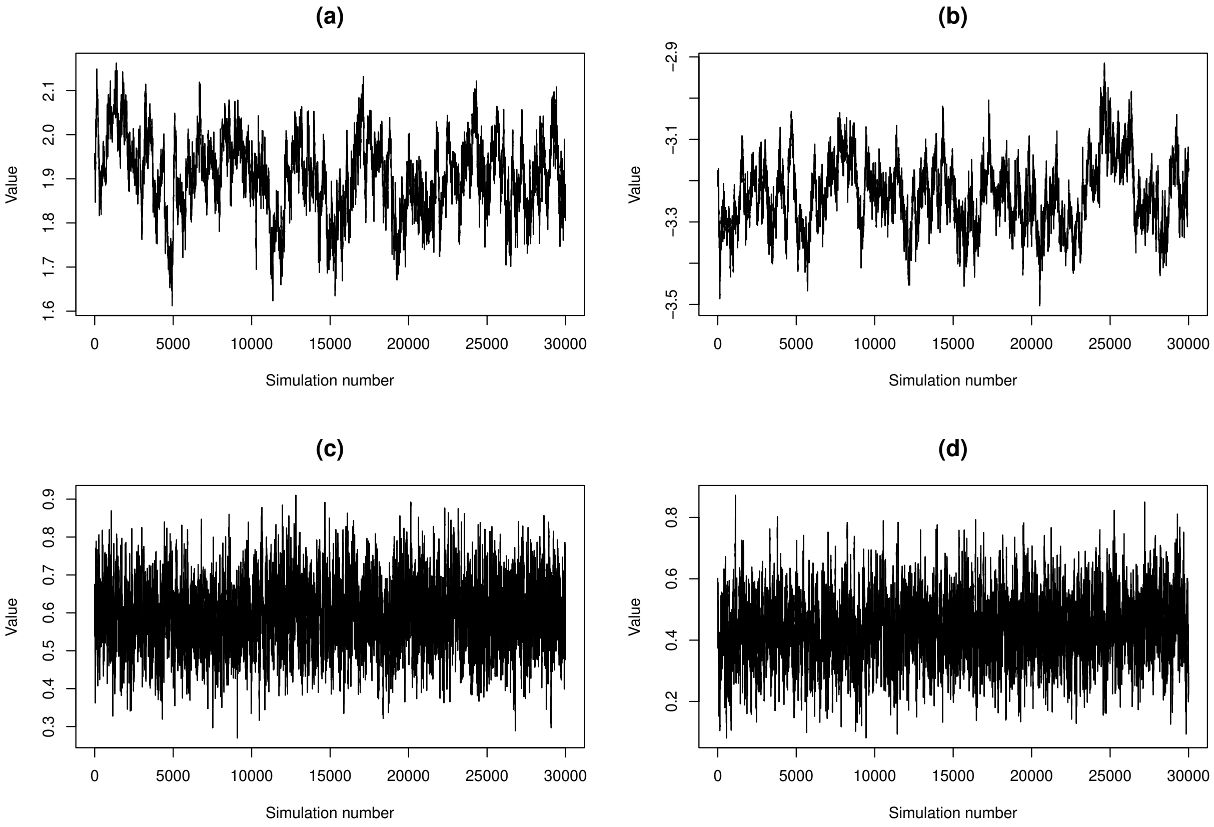

Figure S13: Trace plots for a) γ_2^R, b) γ_4^R, c) γ_2^Q and d) γ_4^Q.

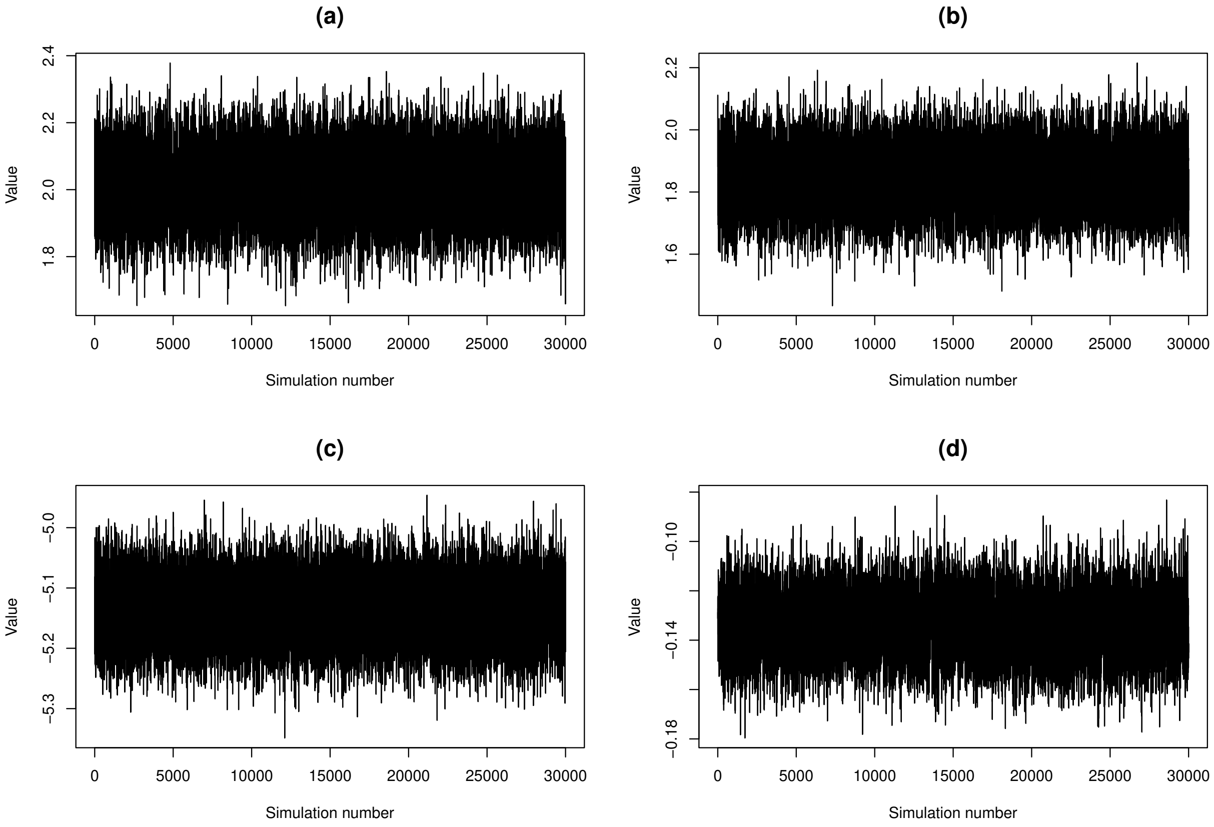

Figure S14: Trace plots for a) γ_2^H, b) γ_4^H, c) γ_2^A and d) γ_4^A.

S.3.2 Cross-validation details

The validation was a two-stage procedure, one stage involving the aerial surveys and the other stage involving the ground surveys. Stage 1 involved a stratified cluster random sample, where the 75 aerial surveys, each covering the entire Masai Mara Ecosystem, were treated as the strata, with survey h having $N_{h,trans}$ aerial transects (ranging from 9 to 57) collectively containing a total of $N_{h,units}$ (ranging from 232 to 705) sampling units (SUs, parallelograms with 3 possible spatial dimensions and sizes 12.5, 25, and 50 km^2). For each survey the procedure performs a balanced bootstrap selection of samples of $N_{h,trans}$ transects 1000 times. When a transect is selected for each survey, all the observations of all the species made on all the sampling units on that transect are automatically

selected as well. Hence, a transect is treated as a cluster associated with multiple observations. Because the bootstrap algorithm is balanced the overall total number of selections or times each transect is selected out of the 1000 replications is the same for all transects for each survey. Out of the 1000 bootstrap samples drawn for each survey, we used only a subsample n_h = 10 in the cross-validation exercise because of the huge computational burden. For each combination of survey and bootstrap replicate, we use Jolly's (1969) Method 2 to calculate an estimate of the total topi population size $P_{i,t}$ (bootstrap iteration i and year-month t) and its standard error. These estimates correspond to Ψ_t in Tables 2 of the main text. We then interpolate the estimated total topi population size to cover all the 174 months spanned by the ground vehicle counts (July 1989 and December 2003) using a semiparametric generalized linear mixed regression model assuming a negative binomial error distribution and a log link function (Piepho and Ogutu, 2007; Ogutu et al., 2014*b*, 2016).

Then Stage 2 uses the results from Stage 1, namely the T = 174 year-month total population size estimates, and results from the ground surveys, on the same year-month time scale, to partition each of those year-month totals into age and sex classes. A given year-month ground survey at time t yields empirical estimates of the proportions by age and sex (proportions calculated from $n(t)$, $q(t)$, $h(t)$, $f(t)$, and $m(t)$-based on Table 2). And those proportions are multiplied against a given $P_{i,t}$.

Next, we provide more detailed descriptions of the cross-validation procedure.

To validate the model, we produced a set of 10 time series of bootstrap estimates of total population sizes for each of the 174 ground survey sampling time points (we call these estimates bootstrap population estimates, as also done in Section 5.2 of the main text) denoted by $\boldsymbol{P}_1, \boldsymbol{P}_2, \ldots, \boldsymbol{P}_{10}$. $\boldsymbol{P}_i = (P_{i,1}, \ldots, P_{i,174})$ is a vector of population estimates, where $P_{i,t}$ denotes the i-th boostrap population size estimates at the t-th time point. Next, we used the bootstrap population size estimates to generate 10 hypothetical time series of age-and sex-structured animal counts each of length 174 months and spanning from July 1989 to December 2003 and having the same age and sex classes as the actual ground sample count data, we call these hypothetical data sets generated data, as also done in Section 5.2 of the main text, using the following method.

First, we calculated from the ground count data the average population proportion across all the 15 years (1989 to 2003) for each age and sex class in each calendar month

(January, February, . . ., December). In other words, 12 different proportions are calculated for each age class or sex class, one for each calendar month. We assumed that the same population proportions for each age and sex class for January to December also hold for the corresponding month for each of the 174 time points (July 1989 to December 2003) for the bootstrap population estimates. Then, we derived estimates of the expected number of animals in each age and sex class by multiplying each bootstrap population estimate with the corresponding sample proportions. We denote these average proportions for January to December by $w_1, \ldots, w_{12}$.

Let,

$NewB(i, t)$ = Estimated number of newborns at time t calculated from the i-th bootstrap sample.

$QB(i, t)$ = Estimated number of quarters at time t calculated from the i-th bootstrap sample.

$HB(i, t)$ = Estimated number of half-yearlings at time t calculated from the i-th bootstrap sample.

$FB(i, t)$ = Estimated number of adult females at time t calculated from the i-th bootstrap sample.

$MB(i, t)$ = Estimated number of adult males at time t calculated from i-th bootstrap sample.

$m(i, t)$ = Month (January, February, . . ., December) at time point t, $m(t) = 1, 2, \ldots, 12$.

Then, $NewB(i, t) = P_{i,t} w_{m(t)}$, $i = 1, \ldots, 10$; $t = 1, 2, \ldots, 174$. This procedure is repeated for each of the other age- and sex-classes.

Next, we resampled the hypothetical count data from these age- and sex-structured population size estimates 10 times using an observation process identical to that described in Section 4.3 of the main text. $N(t)$, $Q(t)$, etc., in Table 2 and in Section 4.3 are replaced by $NewB(i, t)$, $QB(i, t)$, $HB(i, t)$, $FB(i, t)$ and $MB(i, t)$ for each bootstrap sample $i = 1, \ldots, 10$ and each of the $t = 1, 2, \ldots, 174$ ground survey sampling points. Using equations in Table 2 in the main text, $\lambda_k(i, t)$, $k = 1, \ldots, 5$ age-sex classes; $i = 1, \ldots, 10$ hypothetical time series; $t = 1, \ldots, 174$, were generated assuming σ^2 = 10,000,000,000 and $n(t)$, $q(t)$, etc., were also generated. The resulting samples of $n(t)$, $q(t)$, etc., were treated as hypothetical ground sample counts drawn from hypothetical populations of the same sizes as the total population sizes derived from the bootstrap samples for each of the

$t = 1, 2, \ldots, 174$ ground survey sampling points.

Then, for each of the 10 time series of generated ground sample counts, each of length 174 months, and the 10 time series of total population sizes derived from the corresponding bootstrap samples, we ran the state-space model (described in Sections 3.1-3.7 of the main text) and predicted the total population size for each of the 174 time points. Additional details of the cross-validation procedure are provided in Section 4.3 of the main text.

However, running the state-space model separately for each of the 10 time series of generated ground count data sets and producing converged MCMC chains was computationally very time-consuming. To reduce the computing time we used the idea behind the Importance Resampling MCMC (IRMCMC) proposed by Bhattacharya and Haslett (2007) for inverse problems. Though the current problem is not an inverse problem, the idea can be generalised to tackle it. The details of how this generalization was done are provided next in Section S.3.3.

S.3.3 IRMCMC step

Implementation of the IRMCMC step was done as follows. As explained in Section S.3.2 above, we have 10 time series of generated data sets each of length 174 months. We denote them by $Data_k$, $k = 1, 2, \ldots, 10$. In Section 4.1 of the main text, we denoted the vector with elements $\boldsymbol{\gamma}^R$, $\boldsymbol{\gamma}^Q$, $\boldsymbol{\gamma}^H$ and $\boldsymbol{\gamma}^A$ by $\boldsymbol{\gamma}$. We denote the set of all the state processes and initial states by $\theta = \{Pop_0, Pop(t), t = 1, 2, \ldots, 174\}$ and the rest of the parameters by ψ.

The IRMCMC procedure can then be described by the following algorithm.

1. Set $\pi(\theta, \boldsymbol{\gamma}, \psi \mid Data_1)$ to be the importance sampling density.

2. Simulate 200,000 MCMC runs for θ, $\boldsymbol{\gamma}$ and ψ, discarding the initial 100,000 iterations from the full conditional distributions of the parameters given $Data_1$. Denote the iterations after burn-in by $(\theta^{(\ell)}, \boldsymbol{\gamma}^{(\ell)}, \psi^{(\ell)})$, $\ell = 1, 2, \ldots, 100,000$.

3. For each $k = 2, 3, \ldots, 10$,

(a) For each MCMC sample $(\theta^{(\ell)}, \boldsymbol{\gamma}^{(\ell)}, \psi^{(\ell)})$, calculate importance weights as

$$w\left(\boldsymbol{\gamma}^{(\ell)}, \theta^{(\ell)}, \psi^{(\ell)}\right) = \frac{\pi(\boldsymbol{\gamma}^{(\ell)}, \theta^{(\ell)}, \psi^{(\ell)} \mid Data_k)}{\pi(\boldsymbol{\gamma}^{(\ell)}, \theta^{(\ell)}, \psi^{(\ell)} \mid Data_1)} \propto \frac{L(\boldsymbol{\gamma}^{(\ell)}, \theta^{(\ell)}, \psi^{(\ell)} \mid Data_k)}{L(\boldsymbol{\gamma}^{(\ell)}, \theta^{(\ell)}, \psi^{(\ell)} \mid Data_1)} \tag{35}$$

(b) For $m = 1, 2, \ldots, 100$,

i. Sample $\tilde{\boldsymbol{\gamma}}^{(m)}$ from $\boldsymbol{\gamma}^{(1)}, \ldots, \boldsymbol{\gamma}^{(100000)}$ with probability of choosing $\boldsymbol{\gamma}^{(\ell)}$ proportional to $w\left(\boldsymbol{\gamma}^{(\ell)}, \theta^{(\ell)}, \psi^{(\ell)}\right)$.

ii. For every $\tilde{\boldsymbol{\gamma}}^{(m)}$ selected, simulate 1000 MCMC samples from $\pi(\theta, \psi \mid \tilde{\boldsymbol{\gamma}}^{(m)}, Data_k)$.

(c) These 100×1000 draws of θ are samples from the marginal posterior distribution $\pi(\theta \mid Data_k)$. Denote the simulated values by $\hat{\theta}^{(1)}, \hat{\theta}^{(2)}, \ldots, \hat{\theta}^{(100000)}$.

As described in Section S.3.2 the generated data were resampled from the bootstrap population size estimates, which, in turn, were produced from components of the same aerial survey population estimates. Thus, we can assume that the form of the population dynamics is the same as that for all the 10 time series of bootstrap population estimates. So, the same values for ($\boldsymbol{\gamma}^{R(\ell)}$, $\boldsymbol{\gamma}^{Q(\ell)}$, $\boldsymbol{\gamma}^{Y(\ell)}$, $\boldsymbol{\gamma}^{A(\ell)}$) are expected to characterize the birth recruitment and survival probabilities and sex ratios for all the 10 time series of bootstrap population estimates. However, since various demographic segments of the population have different numbers in the different bootstrap population size estimates, $\theta^{(\ell)}$ varies across the different bootstrap population size estimates.

So, we used $\boldsymbol{\gamma}^{(\ell)}$ for the importance sampling step and $\theta^{(\ell)}$ for the MCMC step. The IRMCMC samples $\hat{\theta}^{(1)}, \hat{\theta}^{(2)}, \ldots, \hat{\theta}^{(100000)}$ were used to derive 95% credible intervals for the population size estimates at each of the 174 time points for each of the 10 generated time series $Data_k$, $k = 2, \ldots, 10$.

S.3.4 Sensitivity of topi population dynamics model to variation in predation risk

Predation is an important component of the population dynamics model proposed in Section 3.5 of the main text. In Section 3.6 of the main text, we briefly discuss the predation

risk falling on each age group and how this is represented in the model. Here, we formulate how predation risk affects birth recruitment rate and the survival probability of the different age and sex groups and explore how varying predation risk for each age or sex group affects topi population dynamics.

As defined in Section 3.4 in the main text $\tilde{r}(t)$, $\tilde{S}_q(t)$, $\tilde{S}_h(t)$ and $\tilde{S}_a(t)$ denote the birth recruitment rates for newborns and survival rate of the quarter, half-yearling and adult age classes, respectively, in the absence of predation. Without predation, birth recruitment rate and survival probabilities of the different age groups are primarily driven by forage quality and quantity, competition, disease, etc.

We denote by $\check{P}_r$, $\check{P}_q$, $\check{P}_h$ and $\check{P}_a$ the base predation risk for newborn, quarter, half-yearling and adult age groups, respectively. As discussed briefly in Section 3.5 of the main text, the base predation risk for each age or sex class is multiplied by a seasonal factor to account for the effect of seasonality in prey availability driven by ungulate migration. Also, predation risk varies between adult males and females. Given this information we can exprress predation risk as follows.

For the newborn, quarter and half-yearling age-groups, the predation risk at time t can be given by the following equations (following notations in Section 3.4 in the main text).

$$\tilde{P}_r(t) = \tilde{g}(\ell)\check{P}_r$$
$$\tilde{P}_q(t) = \tilde{g}(\ell)\check{P}_q$$
$$\tilde{P}_h(t) = \tilde{g}(\ell)\check{P}_h,$$

where $\ell = 1$ means t is in the wet season and $\ell = 0$ means t is in the dry season and $\tilde{g}(0) = 0.7$ whereas $\tilde{g}(1) = 1$.

For adult males, predation risk is assumed to be:

$$\tilde{P}_a(t) = 0.999\tilde{g}(\ell)\check{P}_a$$

For adult females, predation risk is defined as follows.

$$\tilde{P}_a(t) = \tilde{g}(\ell)\check{P}_a$$

Finally, the actual birth recruitment and age-specific survival rates can be obtained

using the following expressions.

$$
\begin{aligned}
r(t) &= (1 - \tilde{P}_r(t))\tilde{r}(t) \\
s_q(t) &= (1 - \tilde{P}_q(t))\tilde{S}_q(t) \\
s_h(t) &= (1 - \tilde{P}_h(t))\tilde{S}_h(t), \\
s_a(t) &= (1 - \tilde{P}_a(t))\tilde{S}_a(t),
\end{aligned}
$$

We conducted a simple experiment to informally assess the sensitivity of topi population dynamics to the choice of predation risk for each age or sex class. More specifically, we assess how the total topi population size responds to predation pressure falling on a particular age or sex class.

The details of this experiment are as follows. First we choose a possible value for the total population size, say 10,000. Then, we apply the survival and birth recruitment rates on the initial population size and generate the expected total topi population size for each of the 174 time points. As described in Section 3.5 in the main text, we set the predation risk for each age or sex class based on experimentation with the past data. This run is taken as the benchmark and the generated total population size for each of the 174 time points as the base population size. We denote the estimates of the total population sizes as $\mho^B(t)$ for $t = 1, \ldots, 174$.

Next, we vary the predation risk for each age class (5% for adults, 10% for half-yearlings, 20% for quarter age class and 40% for newborn age class) and assess its impacts on the total population size. First we take the base predation risk for the adult age class (5% annually) and increase it by 5%, 10%, 15% and 20% ($i = 1, 2, 3, 4$) consecutively. For each increment we hold the predation risks for all the other age classes fixed at their base rates and run the state-space model. We note the total population size for each of the 174 time points for each run. We repeat this exercise, in turn, for the predation risk for the newborn (40% annually), quarter (20% annually) and half-yearling (10% annually) age classes ($j = 1, 2, 3, 4$).

We repeated this experiment for three choices of the inital population sizes – 10,000, 20,000 and 40,000 ($k = 1, 2, 3$). Overall, we produced 36 runs for each of the three inital total population sizes and each of the four increments to the base predation risk for each of the four age classes. To ensure that the effect of varying the predation risk is not

confounded with those of the other parameters, the rest of the parameters are kept fixed at their values for the base models and are not updated as would be done in the usual MCMC runs.

For each of the 36 runs, the total population size at each of the 174 time points is compared with the total population size estimate for the corresponding time point from the benchmark run. Suppose $\mho^E_{ijk}(t)$ is the estimate of the total population size from one of the trial runs corresponding to the k-th initial population, j-th age class and i-th increment in the predation risk. Then, the percentage change in the total population size is measured by $\tilde{C_{ijk}}$(t) = $100 \times \frac{\mho^B(t)-\mho^E_{ijk}(t)}{\mho^B(t)}$, for $t = 1, \ldots, 174$. We calculated the average of the percentage changes in the total population size at each of the 174 time points over the three initial population sizes as $\bar{C_{ij}}$(t) = $\frac{1}{3}\sum_{k=1}^{3} \tilde{C_{ijk}}$(t).

We plotted $\bar{C_{ij}}$(t), $t = 1, 2, \ldots, 174$, for all the four increments in the same figure for all the age classes (Figure S15). In the plots, we use a common scale for the y-axis to enable a direct comparison of the four plots. It is clear from the four plots that the total population size is very sensitive to changes in the predation risk for the adult age class but is far less sensitive to the same amount of change in the predation risk for the newborn, quarter or half-yearling age class.

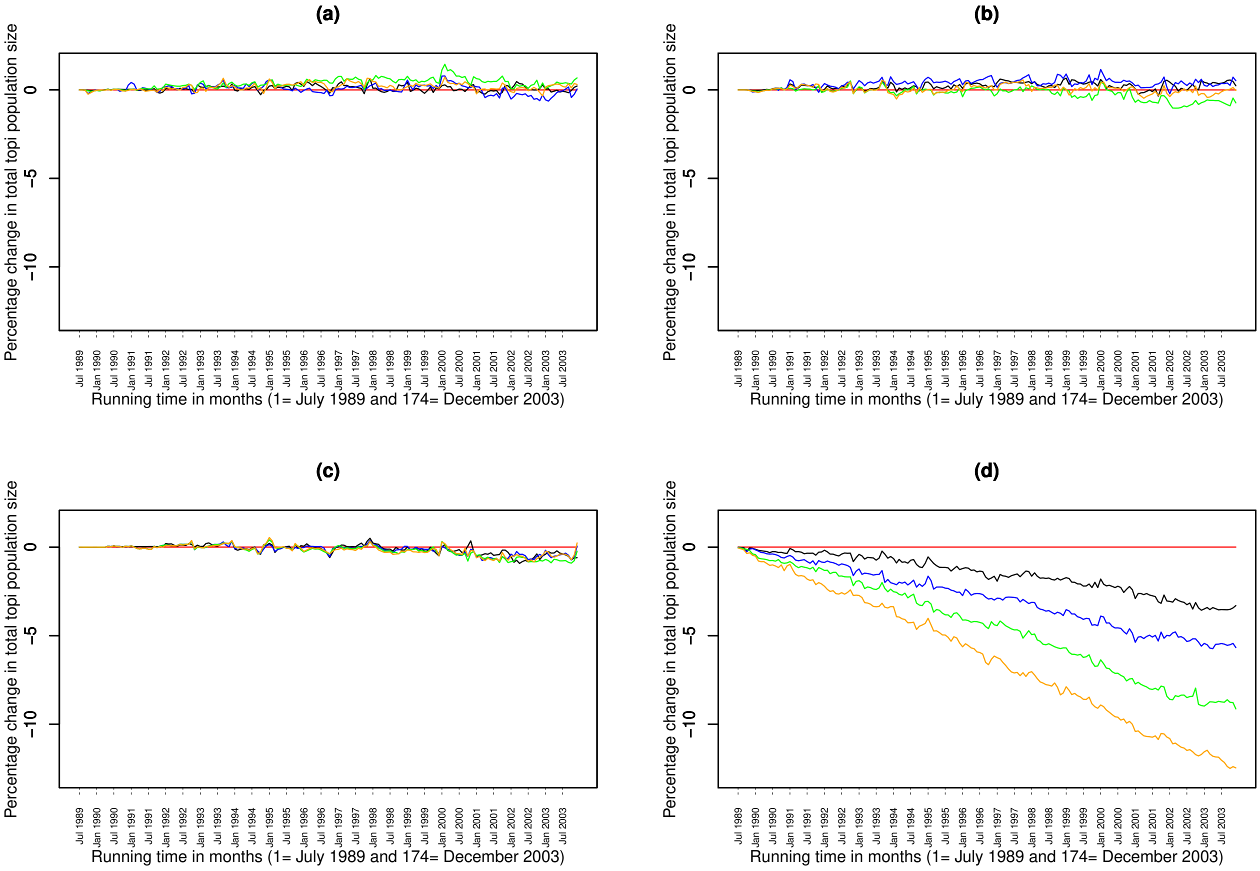

Figure S15: Percentage change in the total topi population size at each of the 174 time points for a 5% (black lines), 10% (blue lines), 15% (green lines) and 20% (orange lines) increment in the base predation risk for the (a) newborn; (b) quarter; (c) half-yearling and (d) adult age class.

S.4 Result details

Using the simulated MCMC values we explored various features of the population dynamics model, specifically focusing on topi population size and the dynamics of birth recruitment and survival rates. In the subsequent subsections we present selected subsets of our findings, for brevity, in graphs and tables.

S.4.1 Dynamics of adult female topi

Females constitute an especially important segment of large herbivore populations, becasue they produce offspring and thus primarily drive population dynamics. We illustrate the dynamics of three subgroups of females in this section. We plot the population trajectories of three subgroups of adult females, female half-yearlings that join the adult female class at time t, $F_a(t)$, adult females that gave birth exactly 8 months ago, $P(t, 8)$, and adult females that gave birth 11 months ago or at least 12 months ago and so are available to conceive, $P(t, 11)$ + $P(t, 12)$ in Figure S16. All the trajectories show a pronounced but time-varying seasonal pattern, reflecting the underlying controlling influence of rainfall seasonality and its impact on forage availability and quality for grazing herbivores, such as topi.

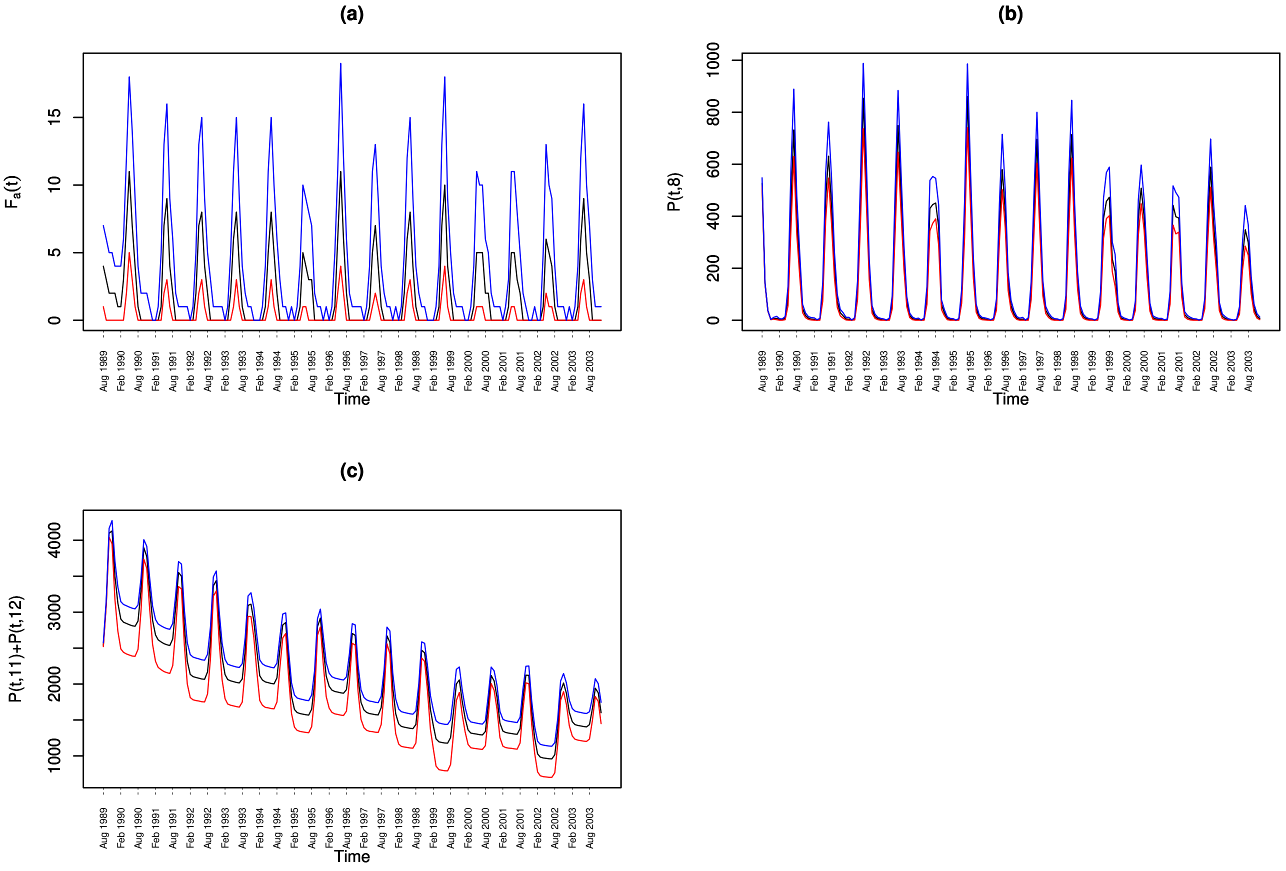

Figure S16: Modeled population trajectories (posterior means) for a) female half-yearlings that join the adult female class at time t ($F_a(t)$), b) number of adult females at time t that gave birth exactly 8 months ago ($P(t, 8)$) and c) the total number of adult females that gave birth 11 months ago or at least 12 months ago and so are available to conceive at time t ($P(t, 11) + P(t, 12)$). The black line at the centre is for the predicted population size, the red line is the 95% lower and the blue line is the 95% upper credible limit of the predicted population size.

S.4.2 Proportions of topi population for each age and sex class

The proportions of newborns, quarters, half-yearlings and adults in the population are plotted in Figure S17a-d.

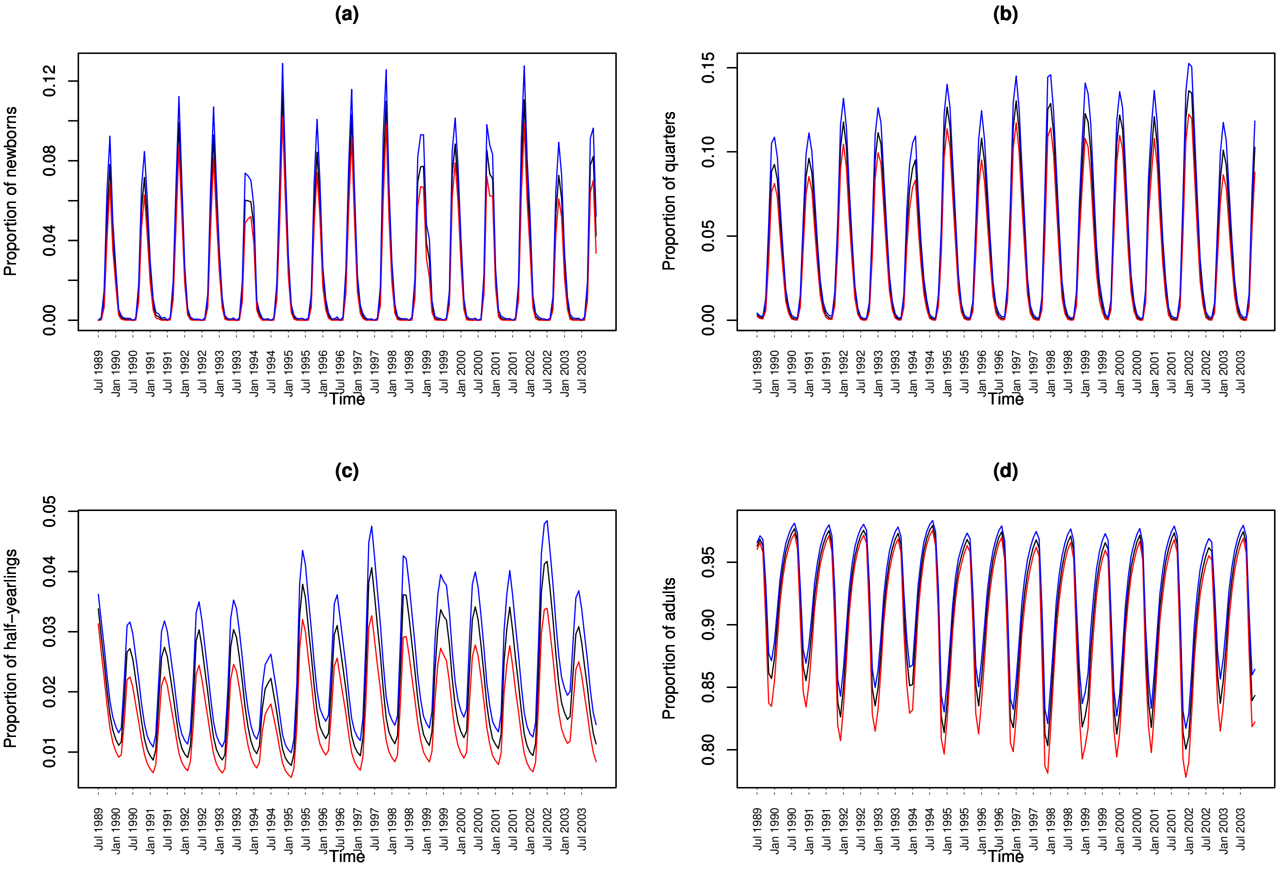

Figure S17: Trajectories of the predicted proportions (posterior means) of a) newborn ($N(t)$), b) quarter-size ($Q(t)$), c) half-yearling size ($G(t)$) and d) adults of both sexes ($F(t)$+$M(t)$) in the predicted total topi population in the Masai Mara Ecosystem during July 1989 - December 2003 (174 months). The black line at the centre is the predicted proportion, the red line is the 95% lower and the blue line is the 95% upper credible limit of the predicted proportion.

S.4.3 Birth recruitment, survival rates and adult sex ratio

The birth recruitment and survival rates for the newborn, quarter size, half-yearling and adult topi age classes are plotted in Figures S18a–d, respectively.

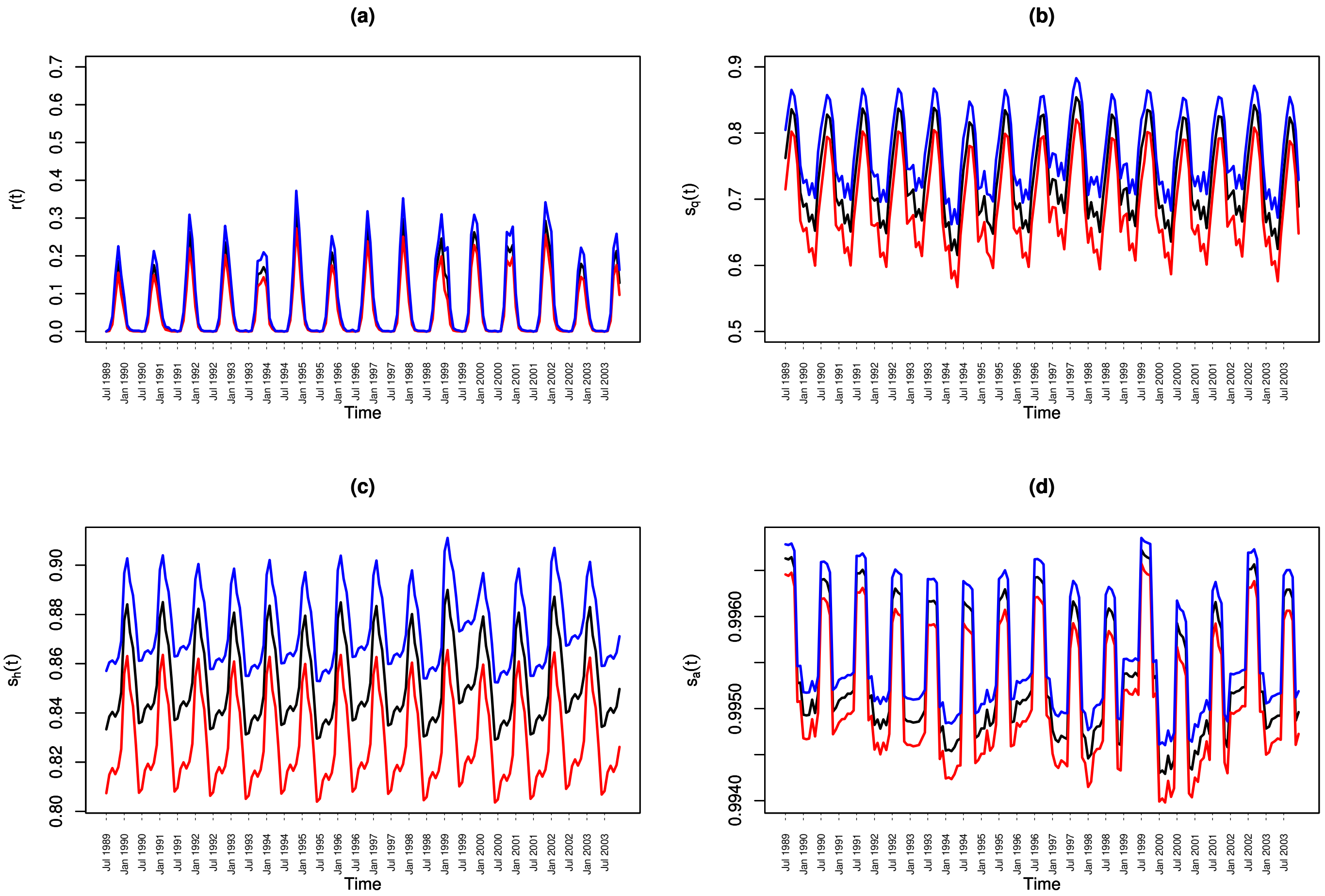

Figure S18: Trajectories of the predicted birth recruitment and survival rates (posterior means) for a) newborn ($N(t)$), b) quarter-size ($Q(t)$), c) half-yearling size ($G(t)$) and d) adult female ($F(t)$) and adult male ($M(t)$) topi in the Masai Mara Ecosystem during July 1989 - December 2003 (174 months). The black line at the centre is for the predicted birth recruitment or suvival rates, the red line is the 95% lower whereas the blue line is the 95% upper credible limit of the predicted rate.

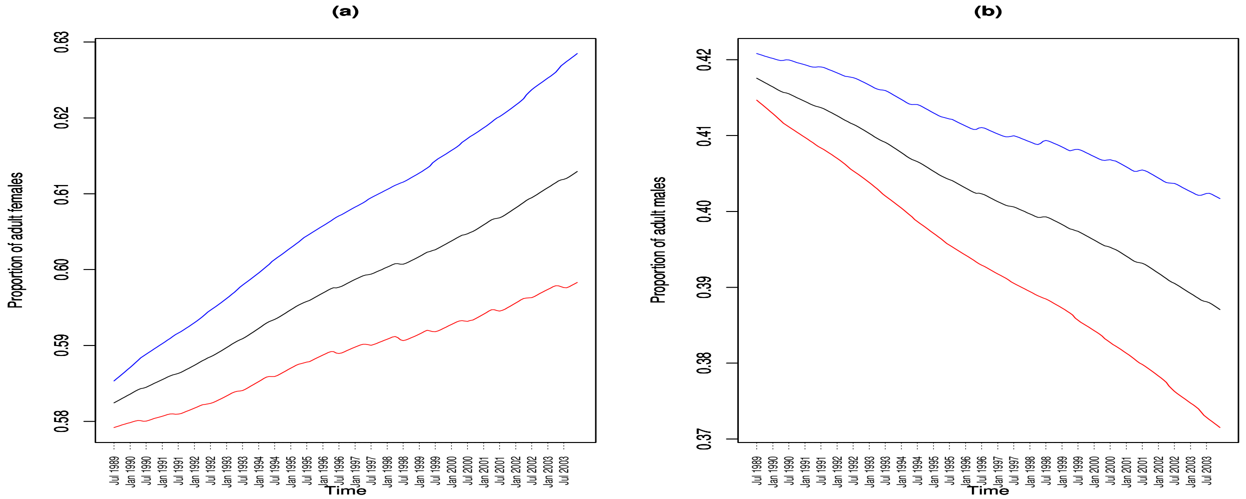

Figure S19: Trajectories of the predicted sex ratios (posterior means) for a) adult female ($F(t)$) and b) adult male ($M(t)$) topi population in the Masai Mara Ecosystem during July 1989 - December 2003 (174 months). The black line at the centre is the predicted sex ratio, the red line is the 95% lower and the blue line is the 95% upper credible limit of the predicted sex ratio.

S.4.4 Determining the birth recruitment, sex ratio and age-specific survival functions

We used a logistic regression model with a binomial error distribution and a logit link function to relate the proportion of adult females that gave birth ($1.7 \times \frac{n(t)}{F(t)}$) to the total population size of topi derived from the aerial surveys and averaged over 7–11 months before the birth month and prior minimum and maximum temperatures and prior rainfall. We similarly related the survival rates for the quarter size class, half-yearling class, adult female and adult male topi to the total topi population size in the preceding 1 to 3 months, seasonality (indexed by month), prior minimum and maximum temperatures and prior rainfall. For modelling adult sex ratio, seasonality (indexed by the dry (June-September) and wet (January-May, November-December) seasons), prior rainfall during the wet and dry seasons and prior temperature are used as covariates in the logistic regression model. The definition of wet and dry seasons used with adult sex ratio is based on the distribution

of topi numbers across months and differs from the meteorological wet season that spans November-June and dry season that covers July-October. The particular lagged values of topi population size, lagged and moving average values of the minimum and maximum temperatures and rainfall considered for each age class and adult sex class are summarized in Table 3 in the main text.

For each age and sex class we considered all the pertinent lagged topi population size, lagged and cumulative moving average values of the minimum and maximum temperatures and rainfall. We observed the principle of marginality (Nelder, 2000) to ensure that interaction, quadratic and cubic terms in the covariates are included only if the main effects are already included in the model. We used forward selection to select variables to retain in the model. We used automatic model selection based on the Akaike (AIC) and Corrected (AICc) Akaike Information Criteria to select the best supported model by using the SAS HPGENSELECT procedure (SAS Institute, 2023). We re-fit the selected best model for each age and sex class and adult sex ratio using the SAS GLIMMIX procedure (SAS Institute, 2023). We estimated and tested parameters in the final model for statistical significance and estimated the 95% confidence limits of the parameters. The selected best model was simplified by sequentially dropping clearly insignificant effects. The estimates of parameters generated by the SAS GLIMMIX procedure (SAS Institute, 2023) were used as hyperparameters of the prior distributions of the regression coefficients.

The functional forms of the logit regression models relating birth recruitment and age-specific survival rates and adult sex ratio to covariates are given in Table 4 of the main text.

S.4.5 Parameter Estimates for birth recruitment, survival rates and adult sex ratio

Summaries of estimates of the parameters γ^R's, γ^Q's, γ^H's and γ^A's are provided in Tables S5–S9. The posterior densities for a few illustrative examples of the parameters from each of the four categories are shown in Figures S20–S23.

Table S5: Estimates (posterior mean) of the parameters (γ^R) of the logistic regression model specified in the first row of Table 4 in the main text used to predict birth recruitment rates ($r(t)$), their standard deviations (SD) and the 95% lower (low95) and upper (up95) credible limits. The estimates are obtained for the model fitted to the full dataset. The notations for the parameters correspond to those for the covariates specified in Table 3 in the main text. Insignificant effects are highlighted in bold font.

Parameter	Mean	SD	low95	up95
Intercept (γ_1^R)	0.56439	0.19607	0.15451	0.91857
$month(t)$ (γ_2^R)	–3.24522	0.07773	–3.40167	-3.09926
$month(t)^2$ (γ_3^R)	1.90593	0.08665	1.73104	2.06846
$month(t)^3$ (γ_4^R)	2.24289	0.17085	1.88393	2.56106
$rain(t)$ (γ_5^R)	–3.23517	0.08238	–3.38511	–3.06744
$rain(t)^2$ (γ_6^R)	–1.50706	0.09739	–1.69776	–1.30532
$Npop(t)$ (γ_7^R)	–3.32808	0.30011	–3.90986	–2.72313
$mintemp(t)$ (γ_8^R)	–0.34619	0.02779	–0.40186	–0.28991
$maxtemp(t)$ (γ_9^R)	**–0.01811**	**0.03670**	**–0.08512**	**0.06543**

Table S 6: Estimates (posterior mean) of the parameters (γ^Q) of the logistic regression model specified in the second row of Table 4 in the main text used to predict survival rates ($s_q(t)$) for quarter-size topi, their standard deviations (SD) and the 95% lower (low95) and upper (up95) credible limits. The estimates are obtained from the model fitted to the full dataset. The notations for the parameters correspond to those for the covariates specified in Table 3 in the main text.

Parameter	Mean	SD	low95	up95
Jan (γ_1^Q)	0.56632	0.08278	0.40608	0.72988
Feb (γ_2^Q)	0.59440	0.08529	0.43157	0.76407
Mar (γ_3^Q)	0.39492	0.09385	0.21209	0.57586
Apr (γ_4^Q)	0.43671	0.10337	0.23428	0.64107
May (γ_5^Q)	0.33148	0.09985	0.13763	0.52723
June (γ_6^Q)	0.63291	0.10026	0.44046	0.83530
July (γ_7^Q)	0.82530	0.11123	0.60622	1.04409
Aug (γ_8^Q)	1.04465	0.11271	0.82696	1.26638
Sep (γ_9^Q)	1.33482	0.10725	1.12092	1.54380
Oct (γ_{10}^Q)	1.33111	0.10026	1.13433	1.52564
Nov (γ_{11}^Q)	1.07603	0.09649	0.88954	1.27186
Dec (γ_{12}^Q)	0.62114	0.09135	0.44174	0.80258
$dry1(t)$ (γ_{13}^Q)	0.26367	0.05216	0.16189	0.36520
$mavrain(3-4,t)$ (γ_{14}^Q)	0.09251	0.04513	0.00540	0.18189

Table S7: Estimates (posterior mean) of the parameters (γ^H) of the logistic regression model specified in the third row of Table 4 in the main text used to predict survival rates ($s_h(t)$) for half-yearling topi, their standard deviations (SD) and the 95% lower (low95) and upper (up95) credible limits. The estimates are obtained from the model fitted to the full dataset. The notations for the parameters correspond to those for the covariates specified in Table 3 in the main text. Insignificant effects are highlighted in bold font.

Parameter	Mean	SD	low95	up95
Jan (γ_1^H)	1.94907	0.09092	1.77037	2.12714
Feb (γ_2^H)	2.01588	0.09289	1.83475	2.19802
Mar (γ_3^H)	1.90217	0.09173	1.72162	2.08174
Apr (γ_4^H)	1.84284	0.09039	1.66653	2.02045
May (γ_5^H)	1.72245	0.08948	1.54683	1.89901
June (γ_6^H)	1.58825	0.08745	1.41724	1.76155
July (γ_7^H)	1.57659	0.08511	1.41077	1.74251
Aug (γ_8^H)	1.61568	0.08209	1.45487	1.77662
Sep (γ_9^H)	1.62990	0.07984	1.47289	1.78523
Oct (γ_{10}^H)	1.61534	0.08057	1.45718	1.77225
Nov (γ_{11}^H)	1.65122	0.08234	1.49136	1.81374
Dec (γ_{12}^H)	1.70962	0.08378	1.54502	1.87353
$earlywet1(t)$ (γ_3^H)	**0.08462**	**0.04794**	**–0.00923**	**0.17842**

Table S 8: Estimates (posterior mean) of the parameters (γ^A) of the logistic regression model specified in the fourth row of Table 4 in the main text used to predict survival rates ($s_a(t)$) for adult topi, their standard deviations (SD) and the 95% lower (low95) and upper (up95) credible limits. The estimates are obtained from the model fitted to the full dataset. The notations for the parameters correspond to those for the covariates specified in Table 3 in the main text. Insignificant effects are highlighted in bold font.

Parameter	Mean	SD	low95	up95
Dry season (γ_1^A)	–5.19996	0.05090	–5.29971	–5.09992
Wet season (γ_2^A)	-5.13727	0.04720	–5.22989	–5.04453
$Apop(t)$ (γ_3^A)	**–3.88** 10^{-6}	**4.58** 10^{-8}	**–3.96** 10^{-6}	**–3.81** 10^{-6}
$lagrain(4,t)$ (γ_4^A)	–0.13382	0.01209	–0.15746	–0.11012
$lagrain(5,t)$ (γ_5^A)	**–0.00317**	**0.01199**	**–0.02686**	**0.02051**
$lagrain(6,t)$ (γ_6^A)	–0.03620	0.01194	–0.05974	–0.01290
$lagrain(7,t)$ (γ_7^A)	–0.02958	0.01125	–0.05162	–0.00749
$wet1(t)$ (γ_8^A)	–0.18666	0.01559	–0.21899	–0.15766

Table S9: Estimates (posterior mean) of the parameters (γ^S) of the logistic regression model specified in the fifth row of Table 4 in the main text used to predict sex ratios ($\phi(t)$) for adult male and female topi, their standard deviations (SD) and the 95% lower (low95) and upper (up95) credible limits. The estimates are obtained from the model fitted to the full dataset. The notations for the parameters correspond to those for the covariates specified in Table 3 in the main text. Insignificant effects are highlighted in bold font.

Parameter	Mean	SD	low95	up95
Dry (γ_1^S)	–1.34142	0.18969	–1.71015	–0.96693
Wet (γ_2^S)	–1.41116	0.20574	–1.81398	–1.00690
$wet1(t)$ (γ_3^S)	0.18742	0.04608	0.09700	0.27758
$dry1(t)^3$ (γ_4^S)	0.06963	0.02997	0.01084	0.12868
$lagrain(0,t)$ (γ_5^S)	0.05469	0.01596	0.02324	0.08589
$rain(7-11,t)$ (γ_6^S)	–0.12657	0.03064	–0.18672	–0.06632
$mintemp(t)$ (γ_7^S)	0.08067	0.00845	0.06417	0.09724
$lagmin(2,t)$ (γ_8^S)	0.02568	0.00776	0.01049	0.04089
$lagmax(1,t)$ (γ_9^S)	**0.00897**	**0.00716**	**–0.00501**	**0.02312**

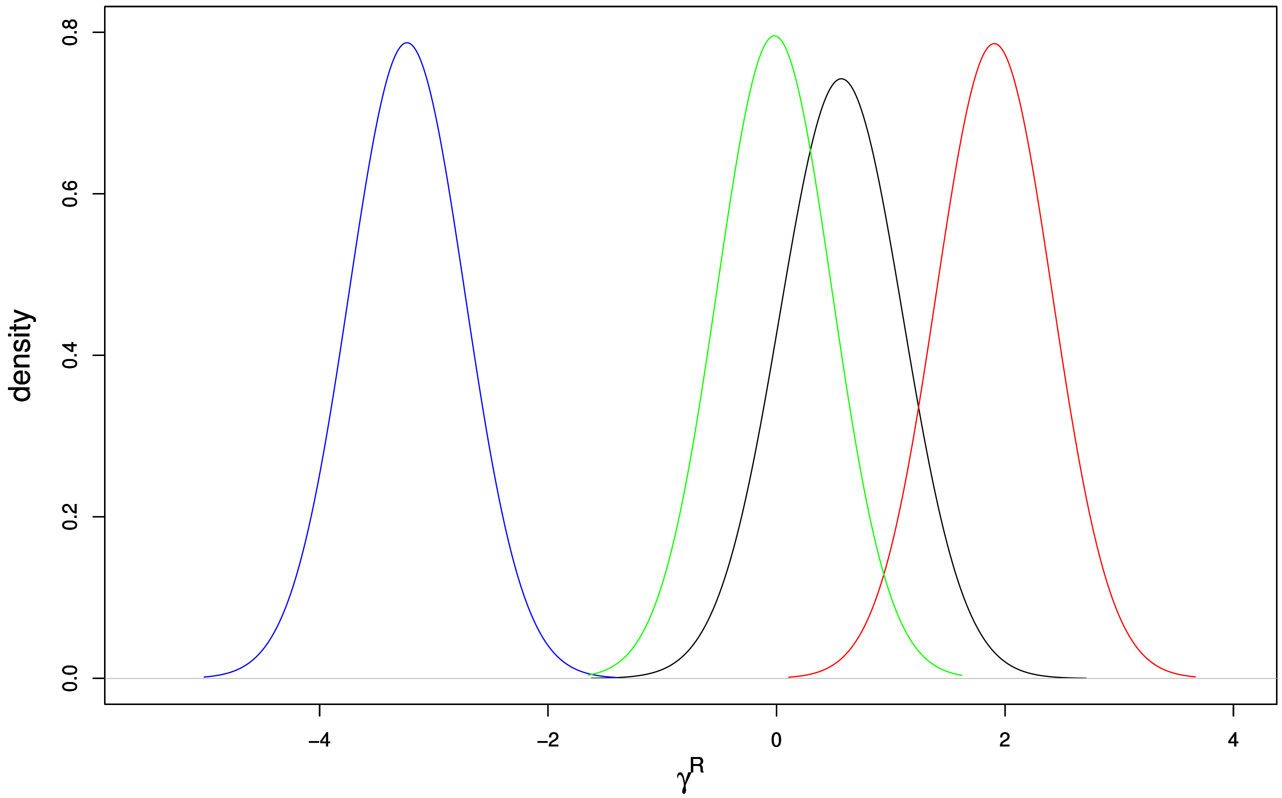

Figure S20: Posterior density of γ_1^R (black), γ_3^R (red), γ_5^R (blue) and γ_9^R (green).

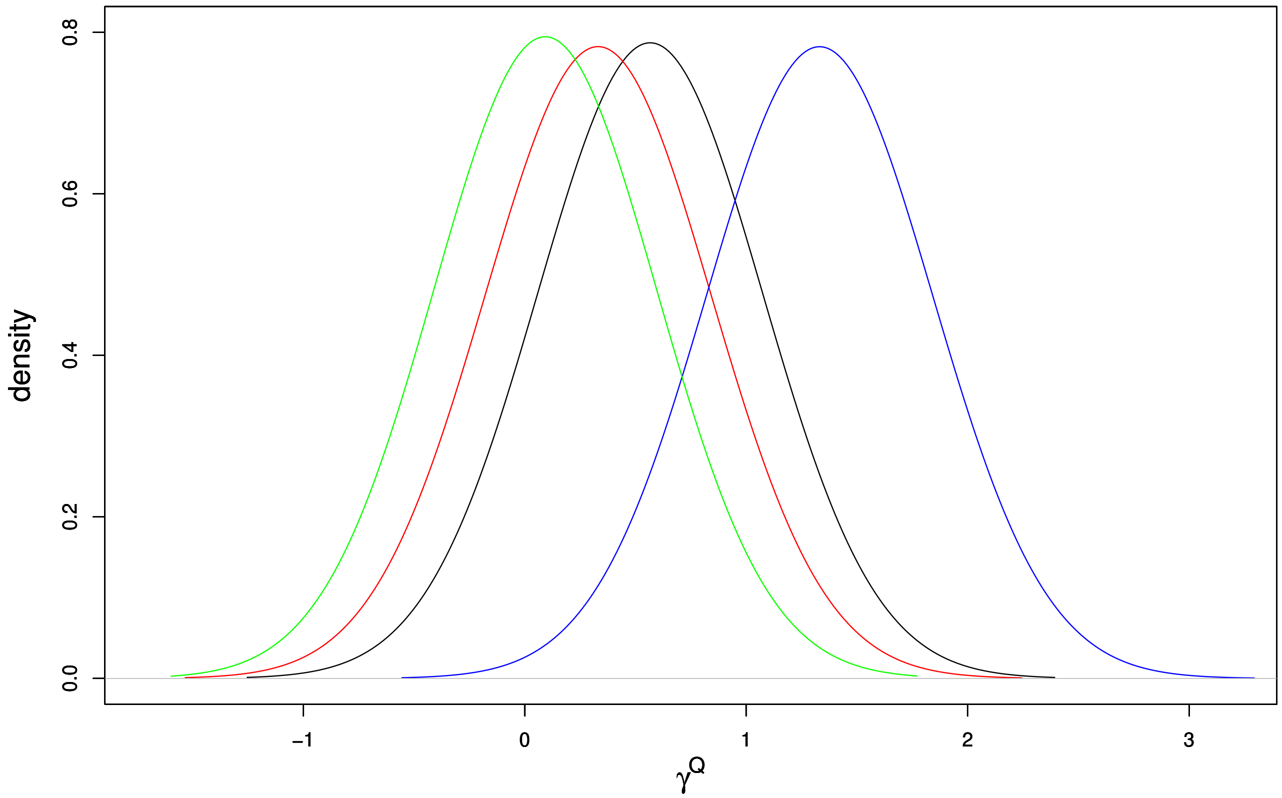

Figure S21: Posterior density of γ_1^Q (black), γ_5^Q (red), γ_{10}^Q (blue) and γ_{14}^Q (green).

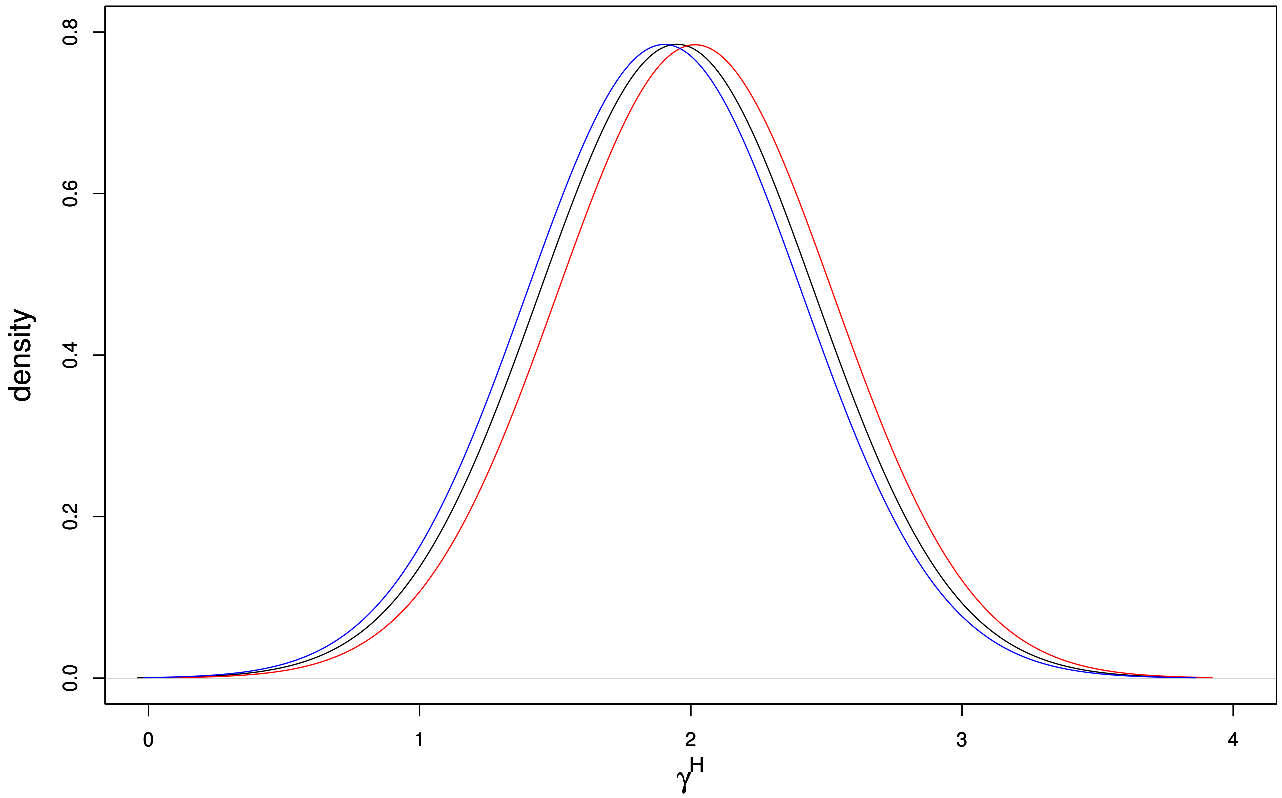

Figure S22: Posterior density of γ_1^H (black), γ_2^H (red) and γ_3^H (blue).

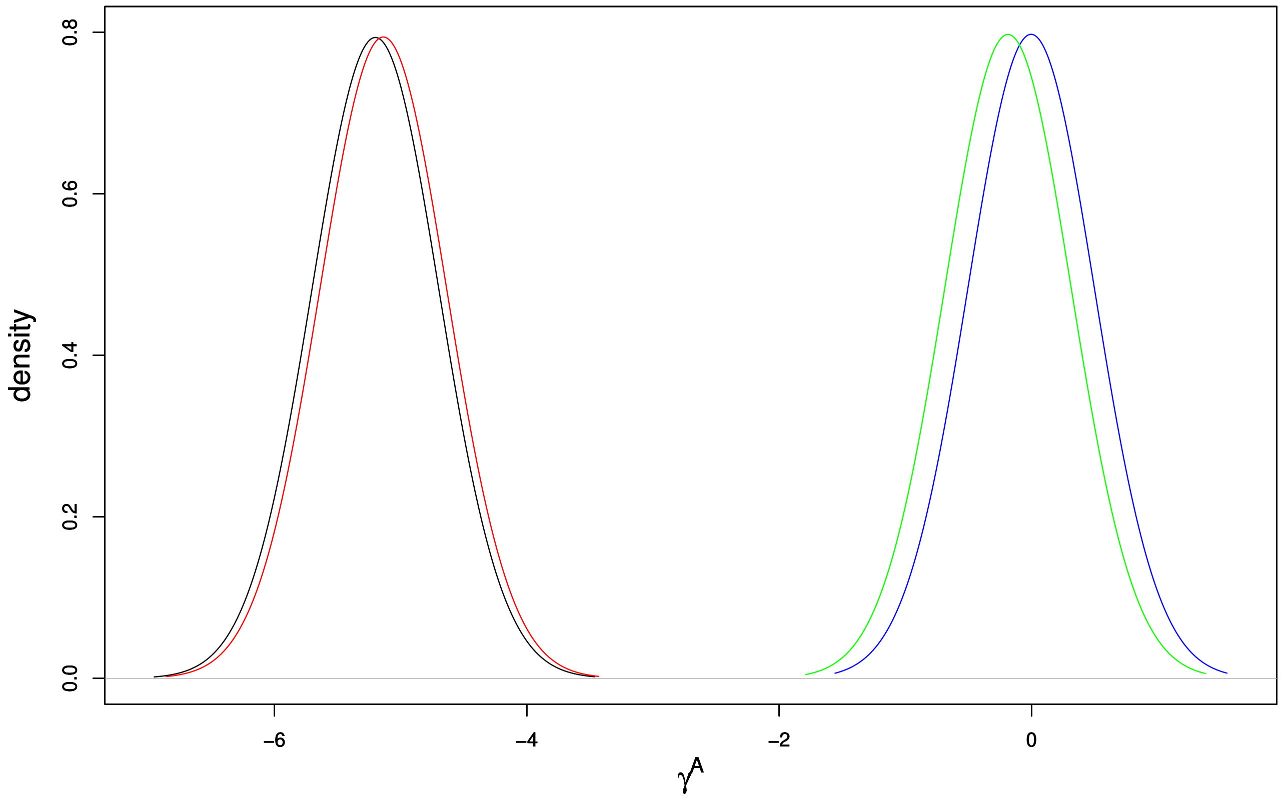

Figure S23: Posterior density of γ_1^A (black), γ_2^A (red), γ_5^A (blue) and γ_8^A (green).

S.4.6 Variation in cohort-specific trajectories

To investigate the between cohort variation and declines over the cohorts through time, we calculated and plotted cohort-specific trajectories, for ages 0 to 19 months, for multiple cohorts on the same time axis. This complements much of the material in Sections 5.2-5.5 of the main text regarding between year variation.

We chose November, the birth peak month, as the starting month for the cohorts for normal (1992), mild drought (1991, 1994), severe drought (1993), extreme drought (1999, 2000), and extremely wet (2001) years. These years were chosen to represent extreme, severe and moderate drought, normal and extremely wet years. Years with

annual rainfall totals falling within the percentiles 0-10, 11-25, 26-40, 41-75, 76-90, 91-95 and 96-100% of the frequency distribution of rainfall were classified, respectively, as extreme, severe or moderate drought years, normal or extremely wet years (Ogutu et al., 2008*a*).

We chose 3500 as the starting number of cohorts for all the seven years. We then estimated the birth-recruitment, survival rates for quarters, half-yearlings and adults from the MCMC simulations for 19 months starting from November of 1991, 1992, 1993, 1994, 1999, 2000 and 2001. These rates and predation risks are applied to all the seven cohorts each of size 3500. The predicted population sizes for each cohort were noted for each of the 19 consecutive months and plotted against time on the same time axis for all the seven cohorts.

On the right vertical axis of the same graph, we plotted the average percentage of the initial population lost each month.

The plot shows that for the initial few months, the population size of the cohorts decline precipitously due to low survival rates. However, over time the survival rates increase and after 19 months all the cohorts contribute comparable numbers of adult recruits to the population ragardless of the level of rainfall prevailing in the birth year (Figure S24). This suggests that some other factor than food availability and quality, indexed by rainfall, such as high predation rate, drive the remarkably low survival rates of the juveniles.

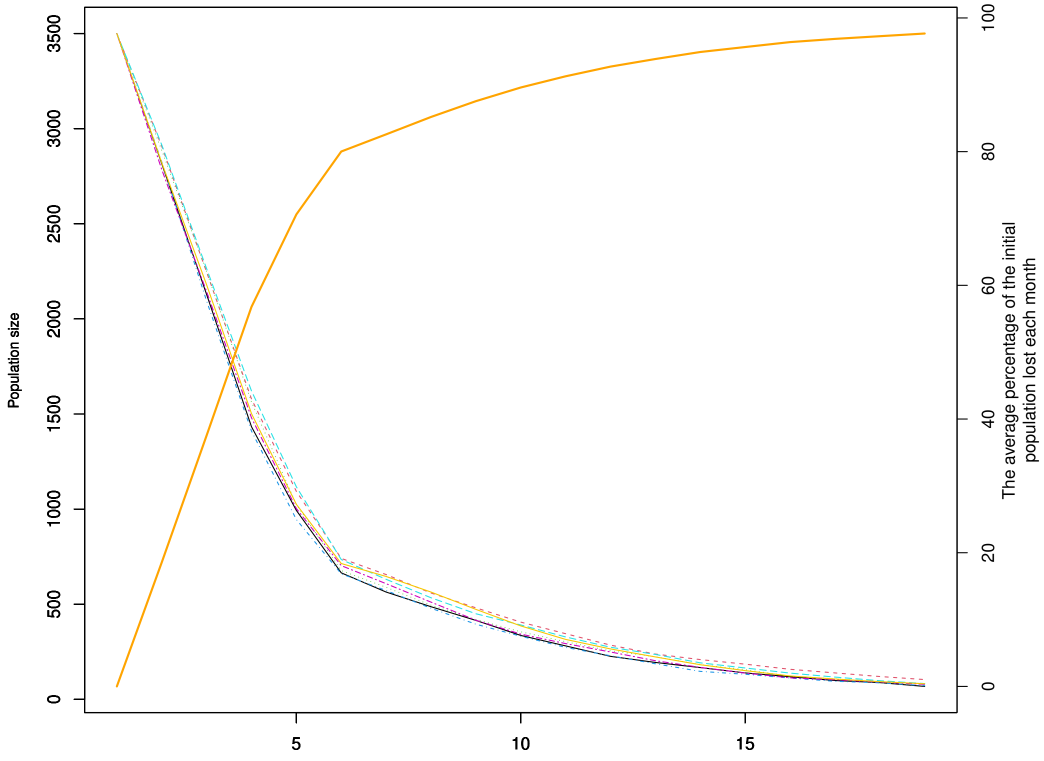

Figure S24: Predicted population size for each of the seven cohorts with a starting population size of 3500 newborn topi and starting month of November over the course of 19 consecutive months (Left vertical axis). The percentage of the initial population lost each month averaged over all the seven cohorts (Right vertical axis).

S.4.7 Population estimates for the four zones of the Mara Ecosystem

The predicted total topi population size for each of the four zones of the Mara Ecosystem, namely Masai Mara National Reserve (Mara Reserve), Mara Conservancies, Loita Plains and Siana are given in Figure S25.

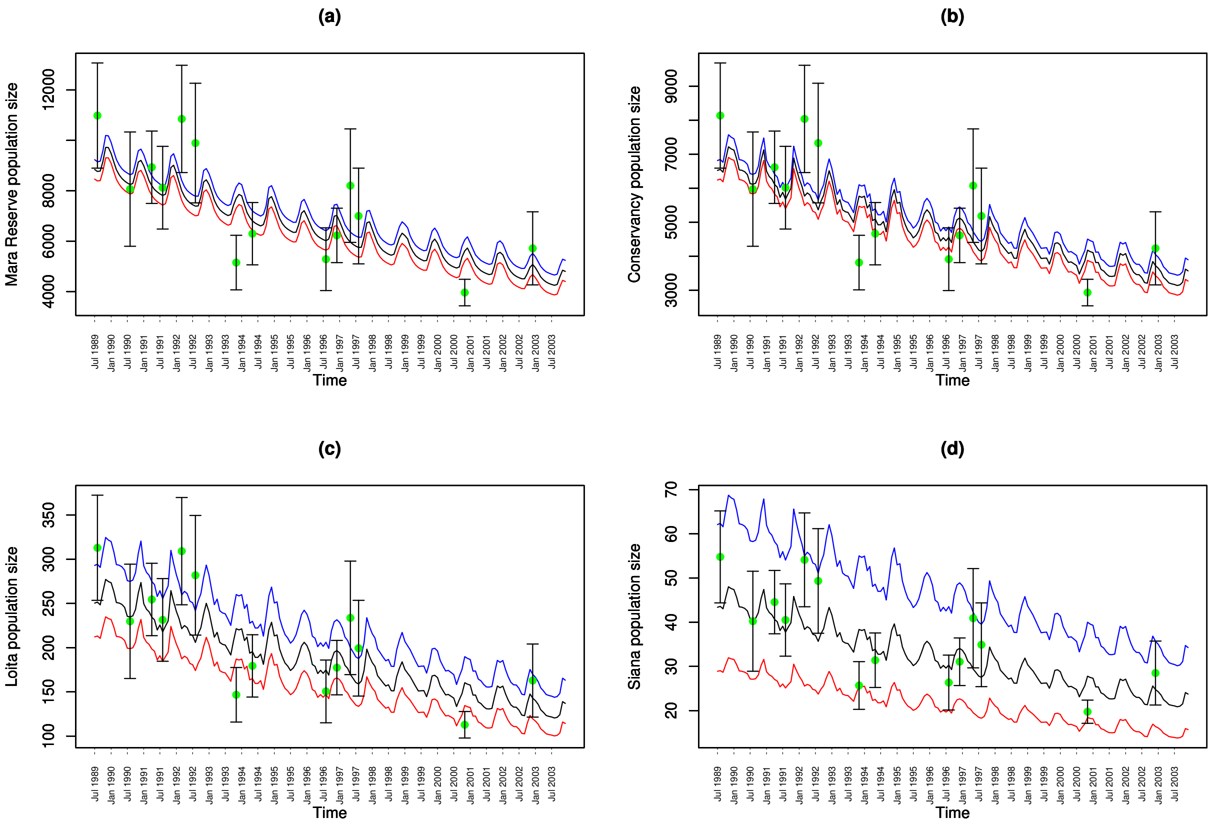

Figure S25: Predicted (black lines) population trajectories (posterior means) and the associated 95% lower (red line) and upper (blue line) credible limits for the total topi population size ($N(t)$+$Q(t)$+$H(t)$+$F(t)$+$M(t)$) for the a) Mara Reserve, b) Mara Conservancies, c) Loita Plains and d) Siana (Siana Plains, Naikara and Loita regions) overlaid with the total population size estimates (green filled circles) and their 95% confidence limits (vertical whiskers) derived from the DRSRS aerial surveys using Jolly's (1969) Method 2. The DRSRS population size estimates and their 95% confidence limits were multiplied by 1.33 to correct for sightability bias (Stelfox et al., 1986).

S.5 Leslie Matrix Formulation of the State Space Model

Here, we derive expressions for the topi population dynamics model as a Linear Gaussian State-Space Model (SSM) as an approximation. The Leslie matrix formulation (Caswell, 2000) makes the SSM's process structure clearer, and together with the use of a normal approximation to the SSM, allows the use of the Kalman filter to calculate the likelihood and making either frequentist or Bayesian inference.

We use matrix representations to derive the expected values for normal distributions and construct the variances using the variances from the binomial and multinomial distributions in the state process equations and the Poisson and Gamma distributions in the observation process equations (Table 2 in the main text) following Newman (1998) and Besbeas et al. (2002). We also use similar matrix mappings between the state vector (aggregated) and the ground survey observations. These complement the Figure 1 schematic diagram and the Table 2 listing of the process and observation model equations in the main text.

Using the notations defined in the main text, we can write the model at the initial stage as follows.

$$Y_t = \begin{bmatrix} N(t) \\ G(t,2) \\ \vdots \\ G(t,19) \\ F_a(t) \\ M_a(t) \\ P(t,1) \\ \vdots \\ P(t,12) \\ M(t) \end{bmatrix} \qquad W_t = \begin{bmatrix} N(t) \\ Q(t) \\ H(t) \\ F(t) \\ M(t) \end{bmatrix} \qquad Z_t = \begin{bmatrix} n(t) \\ q(t) \\ h(t) \\ f(t) \\ m(t) \end{bmatrix}$$

$$\begin{aligned} &E(Y_t \mid Y_{t-1}) \approx L_t Y_{t-1} \\ &W_t = A Y_t \\ &E(Z_t \mid W_t) \approx O_t Y_t, \end{aligned} \tag{36}$$

L_t is defined in Table S10, A is defined in Table S11, and O_t is specified in Table S12 below.

The specification of the distribution for Y_t is given in Table 2 in the main text. Using the mean and variance of the binomial distributions in Table 2 in the main text we specify the mean and variance of the normal distribution approximation to the SSM as follows.

$$\begin{aligned} &[Y_t \mid Y_{t-1}] \sim N(L_t Y_{t-1}, G_t^T L_t Y_{t-1}), \\ &W_t = A Y_t \\ &[Z_t \mid W_t] \sim N(W_t, \Sigma + \mathit{\Omega}(t)) \end{aligned} \tag{37}$$

where G_t is specified in Table S13 and Σ and $\mathit{\Omega}_t$ are specified in Tables S14 and S15, respectively.

Table S10: L_t matrix in equation 36. It is the Leslie matrix in terms of expectation of state vector component

	N(t)	G(t, 2)	G(t, 3)	…	G(t, 6)	G(t, 7)	G(t, 8)	…	G(t, 18)	G(t, 19)	$F_a(t)$	$M_a(t)$	P(t, 1)	P(t, 2)	P(t, 3)	…	P(t, 10)	P(t, 11)	P(t, 12)	M(t)
N(t)	-$s_a(t)$ r(t)	0	0	…	0	0	0	…	0	0	0	0	0	0	0	…	$s_a(t)$ r(t)	$s_a(t)$ r(t)	$s_a(t)$ r(t)	0
G(t, 2)	$s_q(t)$	0	0	…	0	0		…	0	0	0	0	0	0	0	…	0	0	0	0
G(t, 3)	0	$s_q(t)$	0	…	0	0		…	0	0	0	0	0	0	0	…	0	0	0	0
…	0	0	0	…	0	0		…	0	0	0	0	0	0	0	…	0	0	0	0
G(t, 6)	0	0	0	…	0	0		…	0	0	0	0	0	0	0	…	0	0	0	0
G(t, 7)	0	0	0	…	$s_q(t)$	0		…	0	0	0	0	0	0	0	…	0	0	0	0
G(t, 8)	0	0	0	…	0	$s_h(t)$		…	0	0	0	0	0	0	0	…	0	0	0	0
…	0	0	0	…	0	0		…	0	0	0	0	0	0	0	…	0	0	0	0
G(t, 17)	0	0	0	…	0	0		…	0	0	0	0	0	0	0	…	0	0	0	0
G(t, 18)	0	0	0	…	0	0		…	0	0	0	0	0	0	0	…	0	0	0	0
G(t, 19)	0	0	0	…	0	0		…	$s_h(t)$	0	0	0	0	0	0	…	0	0	0	0
$F_a(t)$	0	0	0	…	0	0		…	0	ϕ(t) $s_a(t)$	0	0	0	0	0	…	0	0	0	0
$M_a(t)$	0	0	0	…	0	0		…	0	(1-ϕ(t))$s_a(t)$	0	0	0	0	0	…	0	0	0	0
P(t,1)	$s_a(t)$	0	0	…	0	0		…	0	0	0	0	0	0	0	…	0	0	0	0
P(t,2)	0	0	0	…	0	0		…	0	0	0	0	$s_a(t)$	0	0	…	0	0	0	0
P(t,3)	0	0	0	…	0	0		…	0	0	0	0	0	$s_a(t)$	0	…	0	0	0	0
P(t,4)	0	0	0	…	0	0		…	0	0	$s_a(t)$	0	0	0	$s_a(t)$	…	0	0	0	0
…	0	0	0	…	0	0		…	0	0	0	0	0	0	0	…	0	0	0	0
P(t,10)	0	0	0	…	0	0		…	0	0	0	0	0	0	0	…	0	0	0	0
P(t,11)	0	0	0	…	0	0		…	0	0	0	0	0	0	0	…	$s_a(t)$	0	0	0
P(t,12)	-$s_a(t)$	0	0	…	0	0		…	0	0	0	0	0	0	0	…	0	$s_a(t)$	$s_a(t)$	0
M(t)	0	0	0	…	0	0		…	0	0	0	$s_a(t)$	0	0	0	…	0	0	0	$s_a(t)$

Table S11: The matrix A in equation 36 that aggregates over the state components

	N(t)	G(t, 2)	G(t, 3)	…	G(t, 6)	G(t, 7)	G(t, 8)	…	G(t, 17)	G(t, 18)	G(t, 19)	$F_a(t)$	$M_a(t)$	P(t, 1)	P(t, 2)	P(t, 3)	…	P(t, 10)	P(t, 11)	P(t, 12)	M
N(t)	1	0	0	0	0	0	0	0	0	0	0	0	0	0	0	0	0	0	0	0	0
Q(t)	0	1	1	1	1	0	0	0	0	0	0	0	0	0	0	0	0	0	0	0	0
H(t)	0	0	0	0	0	1	1	1	1	1	1	0	0	0	0	0	0	0	0	0	0
F(t)	0	0	0	0	0	0	0	0	0	0	0	1		1	1	1	1	1	1	1	0
M(t)	0	0	0	0	0	0	0	0	0	0	0	0	1	0	0	0	0	0	0	0	1

Table S12: O_t matrix in equation 36.

	N(t)	Q(t)	H(t)	F(t)	M(t)
n(t)	1	0	0	0	0
q(t)	0	1	0	0	0
h(t)	0	0	1	0	0
f(t)	0	0	0	1	0
m(t)	0	0	0	0	1

Table S13: Matrix for calculating the variance of $[Y_t \mid Y_{t-1}]$ in Equation 37.

	N(t)	G(t, 2)	G(t, 3)	…	G(t, 6)	G(t, 7)	G(t, 8)	…	G(t, 18)	G(t, 19)	$F_a(t)$	$M_a(t)$	P(t, 1)	P(t, 2)	P(t, 3)	…	P(t, 10)	P(t, 11)	P(t, 12)
N(t)	1-$s_a(t)$ r(t)	0	0	…	0	0	0	…	0	0	0	0	0	0	0	…	1-$s_a(t)$ r(t)	1-$s_a(t)$ r(t)	1-$s_a(t$
G(t, 2)	1-$s_q(t)$	0	0	…	0	0		…	0	0	0	0	0	0	0	…	0	0	0
G(t, 3)	0	1-$s_q(t)$	0	…	0	0		…	0	0	0	0	0	0	0	…	0	0	0
…	0	0	0	…	0	0		…	0	0	0	0	0	0	0	…	0	0	0
G(t, 6)	0	0	0	…	0	0		…	0	0	0	0	0	0	0	…	0	0	0
G(t, 7)	0	0	0	…	1-$s_q(t)$	0		…	0	0	0	0	0	0	0	…	0	0	0
G(t, 8)	0	0	0	…	0	1-$s_h(t)$		…	0	0	0	0	0	0	0	…	0	0	0
…	0	0	0	…	0	0		…	0	0	0	0	0	0	0	…	0	0	0
G(t, 17)	0	0	0	…	0	0		…	0	0	0	0	0	0	0	…	0	0	0
G(t, 18)	0	0	0	…	0	0		…	0	0	0	0	0	0	0	…	0	0	0
G(t, 19)	0	0	0	…	0	0		…	1-$s_h(t)$	0	0	0	0	0	0	…	0	0	0
F_a(t)	0	0	0	…	0	0		…	0	1-ϕ(t)$s_a(t)$	0	0	0	0	0	…	0	0	0
M_a(t)	0	0	0	…	0	0		…	0	1-(1-ϕ(t))$s_a(t)$	0	0	0	0	0	…	0	0	0
P(t,1)	1-$s_a(t)$	0	0	…	0	0		…	0	0	0	0	0	0	0	…	0	0	0
P(t,2)	0	0	0	…	0	0		…	0	0	0	0	1-$s_a(t)$	0	0	…	0	0	0
P(t,3)	0	0	0	…	0	0		…	0	0	0	0	0	1-$s_a(t)$	0	…	0	0	0
P(t,4)	0	0	0	…	0	0		…	0	0	1-$s_a(t)$	0	0	0	1-$s_a(t)$	…	0	0	0
…	0	0	0	…	0	0		…	0	0	0	0	0	0	0	…	0	0	0
P(t,10)	0	0	0	…	0	0		…	0	0	0	0	0	0	0	…	0	0	0
P(t,11)	0	0	0	…	0	0		…	0	0	0	0	0	0	0	…	1-$s_a(t)$	0	0
P(t,12)	1-$s_a(t)$	0	0	…	0	0		…	0	0	0	0	0	0	0	…	0	1-$s_a(t)$	1-$s_a(t$
M(t)	0	0	0	…	0	0		…	0	0	0	1-$s_a(t)$	0	0	0	…	0	0	0

Table S14: The matrix Σ in equation 37.

	N(t)	Q(t)	H(t)	F(t)	M(t)
n(t)	σ_1	0	0	0	0
q(t)	0	σ_2	0	0	0
h(t)	0	0	σ_3	0	0
f(t)	0	0	0	σ_4	0
m(t)	0	0	0	0	σ_5

Table S15: Ω_t matrix in equation 37.

	N(t)	Q(t)	H(t)	F(t)	M(t)
n(t)	N(t)	0	0	0	0
q(t)	0	Q(t)	0	0	0
h(t)	0	0	H(t)	0	0
f(t)	0	0	0	F(t)	0
m(t)	0	0	0	0	M(t)

S.6 SAS and R Codes

All the SAS (Supplementary Section-SAScodes.sas) and R (Supplementary Section-Rcodes1.R and Supplementary Section-Rcodes2.R) codes are included in separate files. We describe what each of the 56 R functions or subroutines does in the document called "description_of_R_functions.docx" included in the folder called "Data_dictionary" in Supplementary Materials. We also provide a set of simple instructions on how to run the R code in the document called "Instructions_on_running_the_R_codes.docx" included in the "Data_dictionary folder".

S.7 Extended Acknowledgements

The Masai Mara Ecological Monitoring Program was designed and supervised by Dr. Holly T. Dublin and executed by Messes Paul Chara, John Naiyoma, Charles Matankory and Alex Obara. The program also received financial, material or logistical support from WWF–US, WWF–Sweden, the Darwin Initiative (DICE), the University of British Columbia, United States Fish and Wildlife Service, Kenya Wildlife Service, Cottar's Camp, Kichwa Tembo, Keekorok Lodge/Balloon Safaris and Kerr and Downey Safaris.

WWF–EARPO, the Kenya Meteorological Department and Prof. Kay E. Holekamp, Michigan State University, provided the rainfall data. Chris Shitote helped with extracting and processing the Chirps rainfall and temperature data.

We thank the Director of the Directorate of Resource Surveys of Kenya (DRSRS), Dr. Patrick W. Wargute, for permission to use the aerial survey data. Gordon O. Ojwang, Lucy W. Njino, Rehema B. Rioba, Peter Solonka, Edward Juma, Mohamed Y. Said, Bella A. Omollo, Haron Tanui, Timothy Kavithi and Shem C. Kifugo and student interns at DRSRS, too many to name individually, heartily assisted with the verification, organization, processing and making the DRSRS aerial survey data available. Gordon Ojwang, Mohamed Said and Shem Kifugo helped us understand the structure of the DRSRS data and design of the DRSRS surveys.

We are very grateful to Gordon O. Ojwang, Jully S. Senteu, Stephen M. Ndambuki, Captain Goodwin Wachira and Cindy Obath for permission to use their photos to illustrate the DRSRS survey method. We gratefully thank Sandra van Dyke for collecting the GPS

waypoints of the road transects and Russell Kruska for producing the map of the counting blocks and road transects for the ground sample counts. We also thank Dickson Kaelo and Niels Mogensen for photos of topi in the Masai Mara Ecosystem.

The University of Hohenheim and the Planning for Resilience in East Africa through Policy, Adaptation, Research, and Economic Development (USAID PREPARED) Project, through CAMCO, also provided partial support to JOO during the preparation of this paper.

References

Auger-Méthé, M., Newman, K., Cole, D., Empacher, F., Gryba, R, ., King, A. A., Leos-Barajas, V., Mills Flemming, J., Nielsen, A., and Petris, G. (2021), "A guide to state-space modeling of ecological time series," *Ecological Monographs*, 91(4), e01470.

Bartzke, G. S., Ogutu, J. O., Mukhopadhyay, S., Mtui, D., Dublin, H. T., and Piepho, H. P. (2018), "Rainfall trends and variation in the Maasai Mara ecosystem and their implications for animal population and biodiversity dynamics," *PloS One*, 13(9), e0202814.

Besbeas, P., Freeman, S. N., Morgan, B. J. T., and Catchpole, E. A. (2002), "Integrating mark—recapture—recovery and census data to estimate animal abundance and demographic parameters," *Biometrics*, 58(3), 540–547.

Bhattacharya, S., and Haslett, H. (2007), "Importance re-sampling MCMC for cross-Validation in inverse problems," *Bayesian Analysis*, 2(2), 385–408.

Caswell, H. (2000), *Matrix population models, Volume 1*, MA: Sinauer Sunderland.

Duncan, P. (1975), Topi and their food supply, PhD dissertation, University of Nairobi, Kenya.

Duncan, P. (2014), *Damaliscus lunatus Topi / Tsessebe / Tiang / Korrigum. In: Kingdon, J. and Hoffman M (eds). Mammals of Africa: Volume VI: Hippopotamuses, Pigs, Deer, Giraffe and Bovids*, London: A&C Black.

Dutta, S., and Bhattacharya, S. (2014), "Markov chain Monte Carlo based on deterministic transformations," *Statistical Methodology*, 16, 100–116.

Georgiadis, N. J. (2011), "Introduction: Conserving Wildlife in Kenya's Ewaso Landscape," in *Conserving wildlife in African landscapes: Kenya's Ewaso ecosystem*, ed. N. J. Georgiadis Smithsonian contributions to Zoology; no. 632. Washington, DC: Smithsonian Institution Scholarly Press, pp. 1–10.

Jarman, P. J., and Sinclair, A. R. E. (1979), *Feeding strategy and the pattern of resource partitioning in ungulates*, Chicago: Serengeti: dynamics of an ecosystem, Chicago University Press, 130–163.

Jolly, G. M. (1969), "Sampling methods for aerial censuses of wildlife populations," *East African Agricultural and Forestry Journal*, 34, 46–49.

Nelder, J. A. (2000), "Functional marginality and response-surface fitting," *Journal of Applied Statistics*, 27(1), 109–112.

Newman, K. B. (1998), "State-space modeling of animal movement and mortality with application to salmon," *Biometrics*, 54(4), 1290–1314.

Newman, K., King, R., Elvira, V., de Valpine, P., McCrea, R. S., and Morgan, B. J. (2023), "State-space models for ecological time-series data: Practical model-fitting," *Methods in Ecology and Evolution*, 14(1), 26–42.

Ogutu, J. O., Owen-Smith, N., Piepho, H. P., and Dublin, H. T. (2015), "How rainfall variation influences reproductive patterns of African savanna ungulates in an equatorial region where photoperiod variation is absent," *PloS one*, 10(8), p.e0133744.

Ogutu, J. O., Piepho, H. P., and Dublin, H. T. (2014*a*), "Reproductive seasonality in African ungulates in relation to rainfall," *Wildlife Research*, 41, 323–342.

Ogutu, J. O., Piepho, H. P., Dublin, H. T., Bhola, N., and Reid, R. S. (2008*a*), "El Niño Southern Oscillation, rainfall, temperature and Normalized Difference Vegetation Index fluctuations in the Mara–Serengeti ecosystem," *African Journal of Ecology*, 46, 132–143.

Ogutu, J. O., Piepho, H. P., Dublin, H. T., Bhola, N., and Reid, R. S. (2008*b*), "Rainfall influences on ungulate population abundance in the Mara–Serengeti ecosystem," *Journal of Animal Ecology*, 77, 814–829.

Ogutu, J. O., Piepho, H.-P., Dublin, H. T., Bhola, N., and Reid, R. S. (2009), "Dynamics of Mara-Serengeti ungulates in relation to land use changes," *Journal of Zoology*, 278, 1–14.

Ogutu, J. O., Piepho, H. P., Said, M. Y., Ojwang, G. O., Njino, L. W., Kifugo, S. C., and Wargute, P. W. (2016), "Extreme wildlife declines and concurrent increase in livestock numbers in Kenya: What are the causes?," *PloS one*, 11(9), e0163249.

Ogutu, J., Piepho, H., and Dublin, H. (2014*b*), "Responses of phenology, synchrony and fecundity of breeding by African ungulates to interannual variation in rainfall," *Wildlife Research*, 40(8), 698–717.

Ottichilo, W. K., and Khaemba, W. M. (2001), "Validation of observer, aircraft calibration for aerial animal surveys: a case of the Department of Resource Surveys, Remote Sensing (DRSRS), Kenya," *African Journal of Ecology*, 39, 45–50.

Percival, A. (1924), *A Game Ranger's Note Book*, London: Nisbet.

Percival, A. (1928), *A game ranger on Safari*, London: Nisbet and Co Ltd.

Piepho, H. P., and Ogutu, J. O. (2007), "Simple state-space models in a mixed model framework," *The American Statistician*, 61, 224–232.

Robert, C. P. (2015), "The Metropolis–Hastings Algorithm," *Wiley StatsRef: Statistics Reference Online*, https://onlinelibrary.wiley.com/doi/10.1002/9781118445112.stat07834.

SAS Institute (2023), *System for Statistical Analysis, version 9.4*, SAS Institute Inc., Carey, North Carolina.

Scheel, D., and Packer, C. (1979), *Variation in predation by lions: tracking a movable feast*, Chicago: Serengeti II: Dynamics, management, and conservation of an ecosystem, Chicago University Press, 2–299.

Sidney, J. (1965), "The past and present distribution of some African ungulates," *Transactions of the Zoological Society of London*, 30, 1–36.

Stelfox, J. G., Peden, D. G., Epp, H., Hudson, R. J., Mbugua, S. W., Agatsiva, J. L., and Amuyunzu, C. L. (1986), "Herbivore dynamics in southern Narok, Kenya," *The Journal of wildlife management*, 50(2), 339–347.

Stewart, D. R. M., and Stewart, J. (1963), "The distribution of some large mammals in Kenya," *Journal of The East Africa Natural History Society*, 24(3), 1–52.

Sydney, J. (1965), "The past and present distribution of some African ungulates," *Transactions of the Zoological Society of London*, 30, 1–36.

Veldhuis, M. P., Ritchie, M. E., Ogutu, J. O., Morrison, T. A., Beale, C. M., Estes, A. B., and Wargute, P. W. (2019), "Cross-boundary human impacts compromise the Serengeti-Mara ecosystem," *Science*, 363(6434), 1424–1428.

Vesey-Fitz-Gerald, D. F. (1955), "The Topi Herd," *Oryx*, 3(1), 4–8.

Vesey-Fitz-Gerald, D. F. (1960), "Grazing succession among East African game animals," *Journal of Mammalogy*, 41(2), 161–172.

Western, D. (1979), "Size, life history and ecology in mammals," *African Journal of Ecology*, 17(4), 184–205.
